# Synthesis and p38 Inhibitory Activity of Some Novel Substituted *N*,*N*′-Diarylurea Derivatives

**DOI:** 10.3390/molecules21050677

**Published:** 2016-05-23

**Authors:** Dianxi Zhu, Qifeng Xing, Ruiyuan Cao, Dongmei Zhao, Wu Zhong

**Affiliations:** 1 Key Laboratory of Structure-Based Drug Design and Discovery of Ministry of Education, Shenyang Pharmaceutical University, Shenyang 110016, Liaoning, China; zhudianxi@126.com; 2 Laboratory of Computer-Aided Drug Design and Discovery, Beijing Institute of Pharmacology and Toxicology, 27 Taiping Rd., Beijing 100850, China; xingqf5522@163.com (Q.X.); 21cc@163.com (R.C.)

**Keywords:** P38 MAPK, BIRB-796, SPR, TNF-α

## Abstract

We have identified a novel series of substituted *N*,*N*′-diarylurea *p38*α inhibitors. The inhibitory activity of the target compounds against the enzyme *p38*α, MAPKAPK2 in BHK cells, TNF-α release in LPS-stimulated THP-1 cells and *p38*α binding experiments were tested. Among these compounds, **25a** inhibited the *p38*α enzyme with an IC_50_ value of 0.47 nM and a KD value of 1.54 × 10^−8^ and appears to be the most promising one in the series.

## 1. Introduction 

The mitogen-activated protein kinase *p38* (p38 MAPK) belongs to the serine/threonine kinase family and plays a major role in the regulation, biosynthesis and actions of key proinflammatory mediators like tumour necrosis factor-alpha (TNF-α), interleukin-1β (IL-1β), *p53*, and mitogen-activated protein-kinase-activated protein kinase 2 (MAPKAPK2), *etc.* [1]. According to the literature, there are four known distinct subunits of *p38* MAPK (*p38*α, β, γ and δ). Previous investigations have indicated that *p38*α plays an important role in serious diseases such as chronic obstructive pulmonary disease (COPD), rheumatoid arthritis (RA), psoriasis, and Crohn’s disease [2,3]. In recent years, it has been shown that other diseases such as cancer, neuropathic pain, periodontal diseases and lung inflammation caused by acute gas may also be treated and prevented using *p38*α inhibitors [3,4,5,6,7,8]. However, although some select candidates are in clinical trials, including losmapimod in phase III for treatment of acute coronary syndrome, LY2228820 in phase II for epithelial ovarian cancer and PH-797804 in phase III for treatment of COPD [9,10,11,12], no *p38*α inhibitors are currently available on the market. Therefore, there is continuing need to identify novel bioavailable small molecule inhibitors of *p38*α.

**Figure 1:**
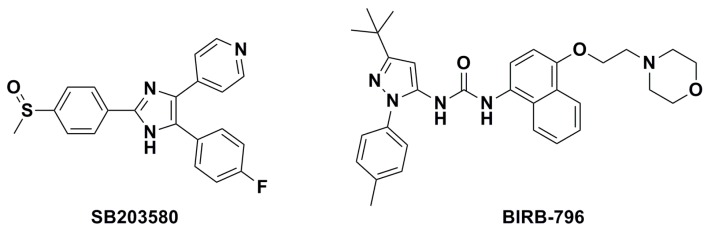
Structures of SB203580 and BIRB-796.

Based on their mode of action, *p38*α inhibitors can be divided into two categories: ATP-competitive inhibitors (e.g., SB-203580) and non-competitive inhibitors (e.g., BIRB-796) that trap the *p38*α in an Asp-Phe-Gly-out (DFG-out) conformation (shown in Figure 1) [13,14,15]. ATP-competitive inhibitors use the ATP binding pocket and inhibit the kinase by directly competing with the binding of ATP [16]. Non-competitive inhibitors typically make use of the ATP binding site, but they also form unique hydrogen bonding and hydrophobic interactions with *p38*α. The non-competitive compounds inhibit *p38*α by maintaining a kinase conformation that is not suitable for ATP binding [17]. Compared with ATP competitive inhibitors, non-competitive inhibitors do not compete directly with the binding of ATP, which could provide a kinetic advantage. In addition, the amino acid residues of the allosteric pocket have very low conservation compared with that of the ATP binding region, therefore, non-competitive inhibitors usually present higher kinase selectivity than ATP competitive inhibitors [18].

BIRB-796 is a classical allosteric *p*38 inhibitor with a *N*,*N*′-diarylurea scaffold. According to the X-ray single crystal structure of *p*38α/BIRB-796, BIRB-796 matched very well with the DFG-out conformation by forming several hydrogen bonding and hydrophobic interactions [19]. TNF-α secretion assays showed that this material inhibits LPS-induced TNF-α production with IC_50_ values of 18 nM and 780 nM for THP-1 cells in human whole blood and in mice *in vivo*, respectively (by 84%, 30 mg/kg p.o.) [20]. The major clinical problem of BIRB-796 is its hepatotoxicity, and the literature has suggested that a naphthalene epoxide intermediate might be the cause. Therefore, some structure modification and structure–activity relationship (SAR) investigations of BIRB-796 were carried out in order to discover novel *p38*α inhibitors.

According to the SAR of BIRB-796, the “urea linkage” occupied a hydrogen-bonding site and is important to maintain the activity [21]. Thus, this “urea linkage” is preserved in our compounds. The naphthyl ring of BIRB-796 pushes quite deeply into the hydrophobic gatekeeper pocket and hydrophobic interactions play a key role in ensuring high activity and selectivity for *p38*α inhibitors [22]. In order to increase the activity and selectivity and reduce or even eliminate the hepatotoxicity, the naphthalene ring was replaced with other aromatic hydrophobic scaffolds, such as phenyl, benzyl or chromene moieties, which allowed for synthetic flexibility and these structural modifications are also the correct size for the small gatekeeper. According to the SAR information for kinase inhibitors, a variety of functional groups are well tolerated by this adenine binding site, and introducing some rigid groups in the site could improve *p*38α inhibitory activity according to the literature [23,24]. We therefore attempted to either replace this morpholinoethoxy moiety with an indazole moiety or replace the morpholinoethoxy ring with a naphthalenol with the overall aim of increasing the binding affinity. BIRB-796 also has a unique allosteric pocket that is created when the activation loop adopts the “DFG-out” conformation. Two selectivity sites could be discovered in the allosteric pocket: one is occupied by the *t*-butyl group, and the other by the *p*-tolyl group. It has been reported that the *t*-butyl group offers a unique interaction with *p38*α that guarantees the inhibitory activity and kinase selectivity [21]. According to the above information, the *t*-butyl group was retained in our compounds, and we tried to replace the 4-tolyl group with other substituted phenyls to investigate the SAR surrounding the phenyl ring unit. According to the information mentioned above, we now report the design and synthesis of some series of new urea derivatives that were then evaluated for their inhibitory activities against MAPKAPK2, TNF-α, and *p*38α.

## 2. Results and Discussion

### 2.1. Chemistry

**Scheme 1:**
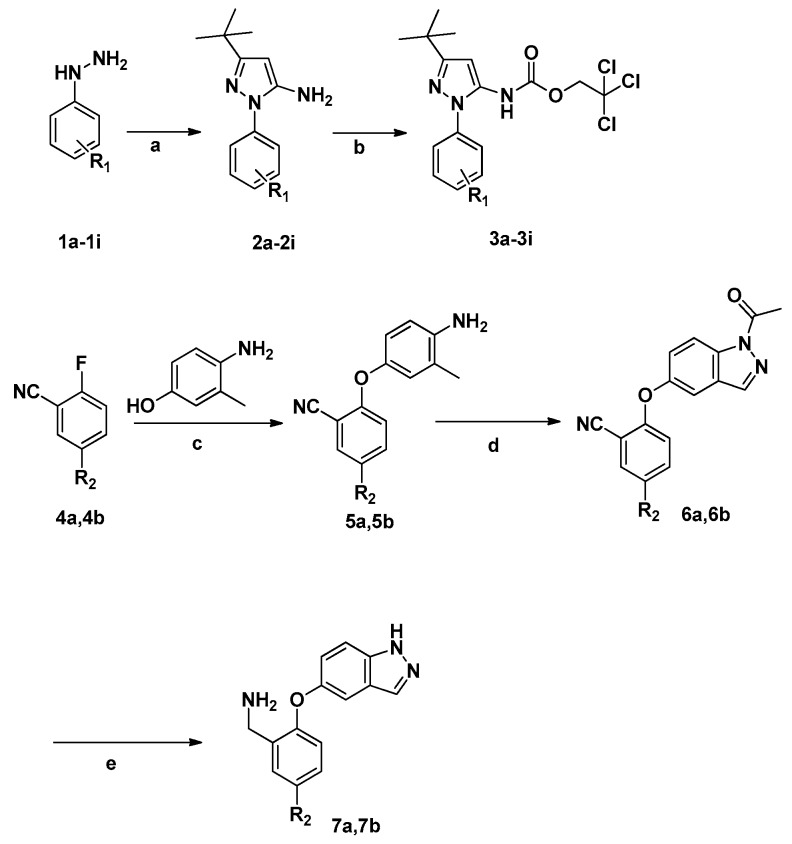
Synthesis of intermediates **2a**–2**i**, **3a**–3**i**, and **4**–**7**(**a,b**). *Reagents and conditions*: (**a**) ethanol, pivaloylacetonitrile, HCl, 80 °C, 6–8 h; (**b**) THF, 2,2,2-trichloroethyl carbonochloridate, NaHCO_3_, 0 °C, 12 h; (**c**) DMSO, 4-amino-3-methylphenol, K_2_CO_3_, 90 °C, 4 h; (**d**) toluene, acetic anhydride, potassium acetate, isopentyl nitrite, 80 °C, 8 h; (**e**) THF, LiAlH_4_, 65 °C, 30–40 min.

**Scheme 2:**
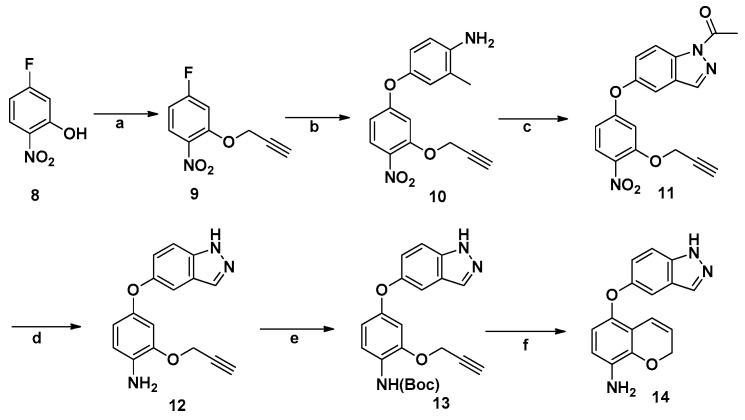
Syntheiss of compound **14**. *Reagents* *and conditions*: (**a**) DMF, 3-bromoprop-1-yne, K_2_CO_3_, rt, 12 h; (**b**) DMSO, 4-amino-3-methylphenol, K_2_CO_3_, 90 °C, 4 h; (**c**) toluene, acetic anhydride, potassium acetate, isopentyl nitrite, 80 °C, 8 h; (**d**) ethanol, SnCl_2_, 80 °C, 2 h; (**e**) DCM, (BOC)_2_O, Et_3_N, 0 °C, 48 h; (**f**) diphenyl ether, microwave, 220 °C, 25 min.

**Scheme 3:**
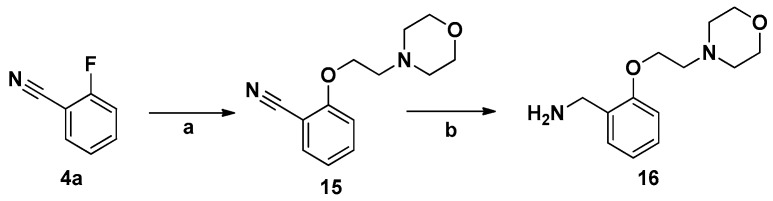
Synthesis of compound **16**. *Reagents and conditions*: (**a**) DMF, 2-morpholinoethanol, sodium hydride, −35 °C, 12 h; (**b**) THF, LiAlH_4_, 65 °C, 30–40 min.

**Scheme 4:**
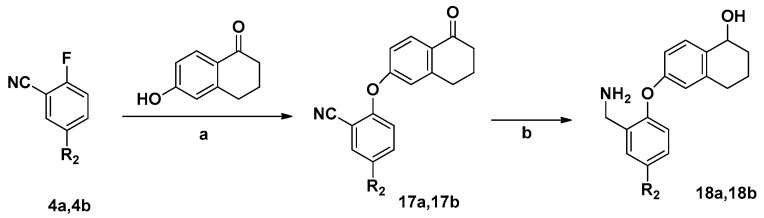
Synthesis of **18**(**a**,**b**). *Reagents and conditions*: (**a**) DMSO, 6-hydroxy-1-tetralone, 90 °C, 4 h; (**b**) THF, LiAlH_4_, 65 °C, 30–40 min.

**Scheme 5:**
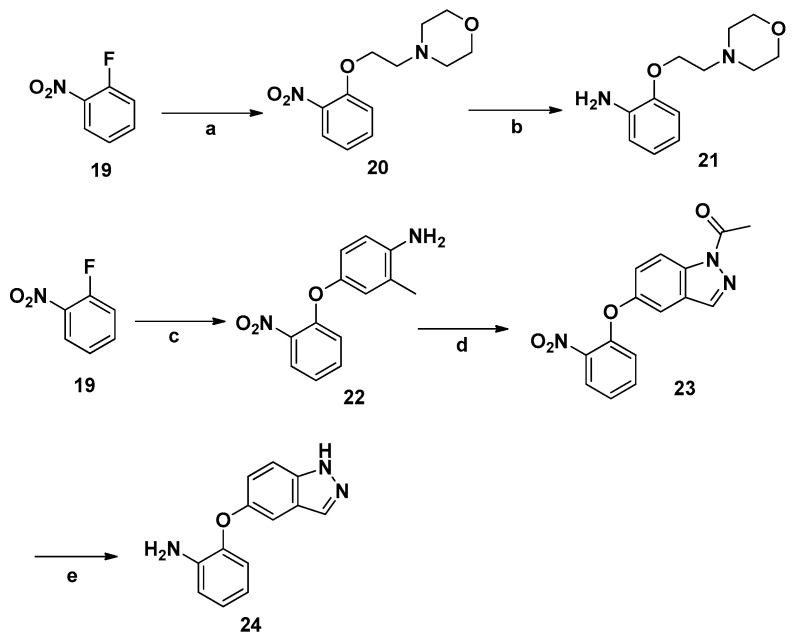
Synthesis of compounds **21** and **24**. *Reagents and conditions*: (**a**) DMF, 2-morpholinoethanol, sodium hydride, −35 °C, 12 h; (**b**) ethanol, SnCl_2_, 80 °C, 2 h; (**c**) DMSO, 4-amino-3-methylphenol, K_2_CO_3_, 90 °C, 4 h; (**d**) toluene, acetic anhydride, potassium acetate, isopentyl nitrite, 80 °C, 8 h; (**e**) ethanol, SnCl_2_, 80 °C, 2 h.

**Scheme 6:**
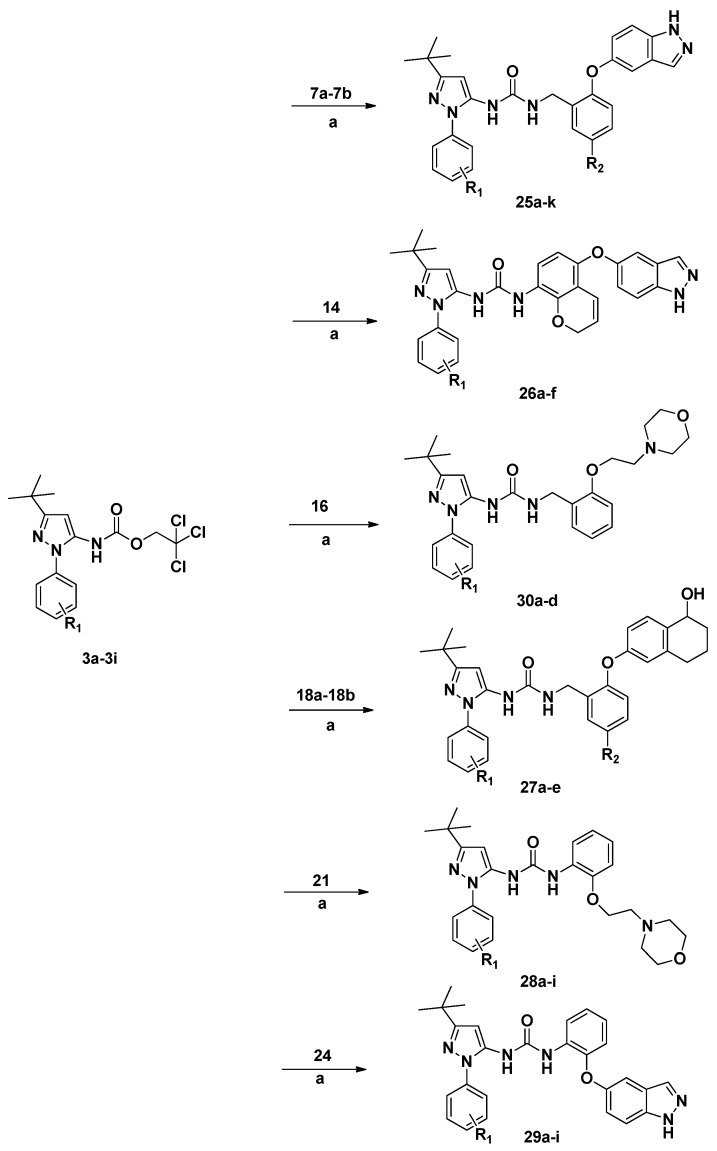
Synthesis of the target compounds **25a**–**25k**, **26a**–**26f**, **27a**–**27e**, **28a**–**28i**, **29a**–**291** and **30a**–**30d**. *Reagents and conditions*: (**a**) DMSO, Et_3_N, 90 °C, 2 h.

The synthetic methods used to prepare our target compounds are summarized in Scheme 1, Scheme 2, Scheme 3, Scheme 4, Scheme 5 and Scheme 6. Firstly, compounds **2a**–**2i** were synthesized using substituted phenylhydrazines **1a**–**1i** and pivaloylacetonitrile in the presence of diluted hydrochloric acid. Treating **2a**–**2i** with 2,2,2-trichloro-ethyl carbonochloridate in THF furnished intermediates **3a**–**3i** (Scheme 1), while the intermediates **7a**–**7b** were synthesized from substituted 2-fluorobenzonitriles **4a**–**4b** (Scheme 1). The intermediates **5a**–**5b** were prepared from **4a**–**4b** and 4-amino-3-methylphenol in the presence of K_2_CO_3_. Intermediates **5a**–**5b** were cyclized with isoamyl nitrite in the presence of potassium acetate and acetic anhydride to generate **6a**–**6b**. Finally, intermediates **7a**–**7b** were synthesized from **6a**–**6b** using LiAlH_4_ in THF at room temperature or H_2_ and Pd/C.

The chromene intermediate **14** was prepared from 2-nitro-5-fluorophenol (**8**, Scheme 2). Treating compound **8** with propargyl bromide in the presence of potassium carbonate gave compound **9**, which upon treatment with 4-amino-3-methylphenol in the presence of K_2_CO_3_ generated the intermediate **10**. Compound **10** was cyclized with isoamyl nitrite in the presence of potassium acetate and acetic anhydride to generate **11**. The intermediate **12** was synthesized by reduction with SnCl_2_ in EtOH or by catalytic hydrogenation under catalysis of Pd/C followed by (Boc)_2_O protection to obtain the compound **13**.

Finally, the chromene compound **14** was cyclized using microwave irradiation under the following conditions: temperature: 220 °C, pressure: 8.0 bar, time: 30 min, power: 250 W [25].

The morpholine intermediate **16** was prepared from 2-fluorobenzonitrile (**4a**, Scheme 3). Treating **4a** with 2-morpholinoethanol in the presence of sodium hydride generated the intermediate **15**. Finally, the intermediate **16** was prepared using LiAlH_4_ in THF at room temperature [26].

The key intermediates **18a**–**18b** were synthesized from the corresponding substituted 2-fluorobenzonitriles **4a**–**4b** by treatment with 6-hydroxy-1-tetralone to furnish the intermediates **17a**–**17b**, followed by LiAlH_4_ reduction (Scheme 4). 

The morpholine intermediate **21** was prepared from 1-fluoro-2-nitrobenzene (**19**, Scheme 5). Treating **19** with 2-morpholinoethanol in the presence of sodium hydride generated the intermediate **20**, which was reduced with LiAlH_4_ in THF to give the target compound. 

Another indazole intermediate **24** was also prepared from 1-fluoro-2-nitrobenzene (**19**, Scheme 5) by treating **19** with 4-amino-3-methylphenol in the presence of K_2_CO_3_ to generate the intermediate **22**, which was cyclized with isoamyl nitrite in the presence of potassium acetate and acetic anhydride to generate **23**. Finally, the intermediate **24** was prepared by LiAlH_4_ reduction of **23** in THF.

The products of interest **25a**–**25k**, **26a**–**26f**, **27a**–**27e**, **28a**–**28i**, **29a**–**29i**, **30a**–**30d** were obtained in quite good yields by the reaction of **3a**–**3i** with the intermediates **7**, **14**, **16**, **18**, **21** and **24** in the presence of DIEA in DMSO via a microwave-assisted method under the following conditions: temperature: 55–100 °C; power: 60–100 W; pressure: 8.0 bar; time: 10–20 min [27,28]. We chose the microwave-assisted pathway as a new method because it is easier and quicker and because yields are improved to a certain degree.

### 2.2. Biology

#### 2.2.1. MAPKAPK2 Inhibitory Activity 

In order to study *p38*α inhibition in a cell system, we investigated the ability of all compounds to inhibit the phosphorylation of mitogen-activated protein-kinase-activated protein kinase 2 (MAPKAPK2). Each compound was prepared at seven concentrations (1, 3, 10 and 30 nM, 0.1 and 0.3 μM), and the percentages of inhibition were calculated from the numbers of cells after treatment with the different compounds. The results are displayed in Table 1.

#### 2.2.2. *p38*α Assay

To assess the *p*38α inhibition in an enzyme system, we selected the compounds that showed good inhibitory activity against MAPKAPK2 and monitored their ability to inhibit the phosphorylation of activation transcription factor (2ATF-2) in the enzyme system in an *in vitro* assay. The results are shown in Table 1.

#### 2.2.3. Inhibition of TNF-α Production in THP-1 Cells 

To assess the inhibition of TNF-α release from THP-1 cells after LPS stimulation, we chose the compounds that showed good inhibitory activity against the *p38*α enzyme and MAPKAPK2 and monitored their ability to inhibit TNF-α release from THP-1 cells after LPS stimulation. The results are also shown in Table 1.

#### 2.2.4. Surface Plasmon Resonance (SPR)

**Figure 2:**
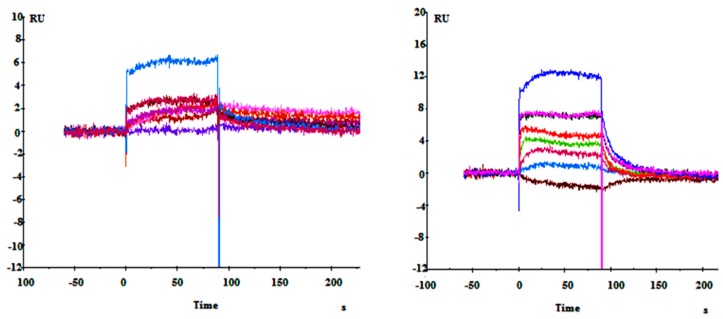
p38α affinity measurement and analysis chart for 25a (**left**); and SB203580 (**right**). Concentration series (0.003–1 μM) of inhibitor with different color lines enzyme kinetic characteristics were determined by surface plasmon resonance on the inactive form of the kinase using a Biacore instrument.

To assess the binding ability between the compounds and *p38*α protein, we used SPR for kinase inhibitor interaction studies. The results are shown in Table 2 and Figure 2.

### 2.3. Discussion

MAPKAPK2 is a well-known substrate of *p38*α. It forms an adduct with *p38*α, and when a stress signal is received, this protein complex is disrupted because of phosphorylation of *p38*α [29]. Thus, MAPKAPK2 could be used as an effector of *p38*α by phosphorylating further substrates. Certain compounds showed good activities, and compounds **25a**–**25k** and **27a**–**27e** were also profiled for their *p38*α inhibitory activity and ability to inhibit TNF-α release in the lipopolysaccharide (LPS)-stimulated human acute monocytic leukaemia cell line (THP-1). The activities and structures of the compounds revealed certain structure-activity relationship information. Overall, the R_1_ and R_2_ substituents have little effect on the activity of compounds, but the inhibitory activity was reduced when R_1_ was substituted with a sulphamoyl group compared with R_1_ substituted with another group, as in compounds **25a**–**25k**, **26a**–**26f**. The polarity of compounds with a sulphamoyl group at the R_1_ position is higher, therefore, the activities of **25k** and **26c** were lower than those of the other compounds owing to their low transport through the cell membrane. Second, the activities of compounds **25i**, **25j**, **27d** and **27e** were lower than those of **25h**, **25a**, **27b** and **27a**, probably because the fluorine atom can affect the ability to interact with the small gatekeeper of the *p38*α protein.

**Figure 3:**
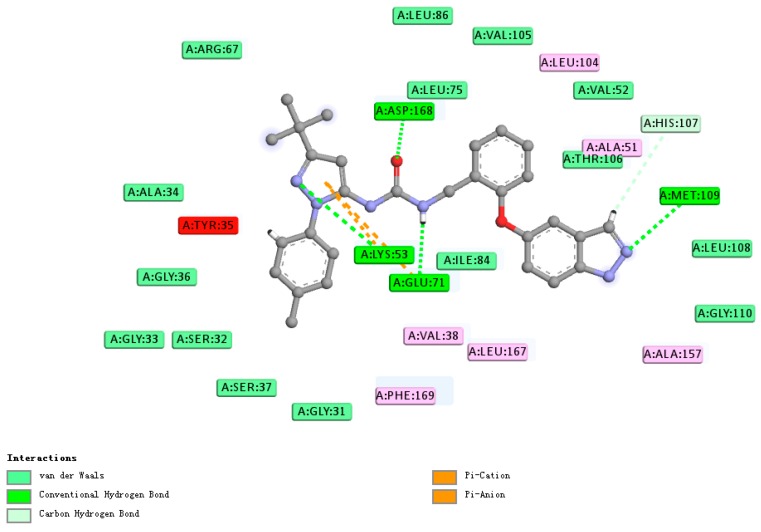
Structure of compound **25a** and a two-dimensional *p38* MAPKα/**25a** interaction map.

In a comparison of the activity of compounds **25a**–**25k** and BIRB-796 with those of **26a**–**26f**, **29a**–**29i** and **28a**–**28i**, **29a**–**29i** and **28a**–**28i** in particular showed almost no inhibitory activity. These results demonstrate that the naphthyl ring of BIRB-796 is important for maintaining the inhibitory activity. We consider that the length of the naphthyl ring and chromene affects the anti-*p38*α activity of the corresponding derivatives. However, the activity of chromene urea compounds was poor compared with that of the corresponding benzylurea analogues or BIRB-796, which indicates that the chromene ring could not replace the naphthyl ring without reducing activity. Perhaps the chromene ring does not offer a correct interaction with the *p38*α protein. Although the structures of compounds **25a**–**25k** and **29a**–**29i** are similar, compounds **29a**–**29i** showed almost no inhibitory activity compared with compounds **25a**–**25k**, and similar activity as **28a**–**28i**. We consider that the phenyl ring substituents in **28a**–**28i** and **29a**–**29i** cannot replace the hydrophobic interactions of the naphthyl ring. A docking model of the *p38*α/**25a** complex was built using Discovery Studio (PDB code: 1KV2) and is shown in Figure 3. Compound **25a** fitted very well in the small gatekeeper position (Thr106) in Figure 3, while the **29a**-linked phenyl ring system could not insert into hydrophobic region I and provide a complementary interaction surface. According to the previous SAR discussion, compounds **30a**–**30d** with a directly linked benzyl ring system should show good activity, but the results indicated that compounds **30a**–**30d** also showed poor activity, similar to compounds **28a**–**28i**. In analysing the structure activity relationship of all compounds, we considered that **30a**–**30d** could not bind with the *p38*α protein in the correct configuration like BIRB-796 because of the flexibility of the molecules (ethoxymorpholine and benzyl ring), while the rigidity of the indazole and 1,2,3,4-tetrahydronaphthalene moieties in compounds **25a**–**25k** and **27a**–**27e** ensured that the active configuration was the same as in BIRB-796. In summary, compounds substituted with a rigid indazole instead of a morpholinoethoxy could show good *p38*α inhibitory activity performance. The indazole also offers a hydrophobic interaction with *p38*α. The benzyl ring system instead of a naphthyl ring effectively occupied the small gatekeeper (Thr106) by forming hydrophobic interactions with the hydrophobic region I of the *p38*α protein, therefore, it was observed that compounds **25a**–**25k**, and especially **25a**, stood out due to their high activity.

According to the information in Figure 3, the structure of **25a** permits key interactions with *p38*α which are similar to those of BIRB-796. In addition, the important amino acids (Thr106, Lys53 and Met109) that bind with *p38*α also preserve the activity. First, the nitrogen atom of the indazole in **25a** directly interacts with Met109 of *p38*α the same as pexmetinib, which could explain the previously discussed high activity of **25a**. In addition, the *p38*α inhibitory activity of **25a** was better than that of pexmetinib, which suggests that the unsubstituted indazole offers better binding ability with *p38*α [30,31,32,33,34]. Second, **25a** occupied the small gatekeeper (Thr106) region by forming hydrophobic interactions with the hydrophobic region I of the *p38*α protein. (Figure 3). Finally, the π-cation interaction between the pyrazole ring and the side chains of Lys53 also plays a role on the activity. The above data also could explain why the binding capacity of **25a** (KD = 1.54 × 10^−8^) was better than that of SB203580. The binding affinity of compound **25a** is lower than literature value of the reference BIRB-796, however it is more potent than BIRB-796 in other assays. We consider that SPR is a rough binding assay method, as the data could only explain binding capacity with the protein instead of binding capacity with the active site.

## 3. Materials and Methods

### 3.1. General Procedures

All reagents and solvents were used as received from commercial sources. ^1^H- and ^13^C-NMR spectra were recorded at 400 MHz and 100 MHz, respectively, on a JNM-ECA-400 instrument (JEOL, Tokyo, Japan) in CDCl_3_ or DMSO-*d*_6_, respectively. Proton and carbon chemical shifts are expressed in parts per million (ppm) relative to internal tetramethylsilane (TMS), and coupling constants (J) are expressed in Hertz (Hz). The splitting pattern abbreviations are described as follows: multiplicity (s: singlet, d: doublet, dd: double doublet, ddd: double double doublet, dm: double multiplet, ds: double single, dt: double triplet, t: triplet, td triple doublet, tm, triple multiplet, tt: triple triplet, q: quartet, quint: quintuplet, m: multiplet, br: broad). The electronic information of compounds are available in the Supplementary Materials. Infrared (IR) spectra were recorded with a Perkin-Elmer 1330 infrared spectrophotometer (PerkinElmer, Waltham, MA, USA) using KBr as solid matrix. Low-resolution mass spectra were obtained using a 3000LC/MS instrument (API, Rockwell, MD, USA) equipped with an ESI source or an 620BTOF LC/MS equipped with an ESI source (Agilent Technologies, Santa Clara, CA, USA). Purities of the compounds were established by analytical HPLC, which was carried out on an Agilent HPLC system. HPLC analysis conditions were as follows: Diamonsil C18 column (250 × 4.6 mm, 5 μm); flow rate, 1.0 mL·min^−1^; DAD detection at 245 nm; mobile phase: 60% acetonitrile in water.

### 3.2. Synthesis

#### 3.2.1. General Method for the Synthesis of **2a**–**2i**

A solution of the corresponding substituted phenylhydrazine hydrochloride (1 equiv.) and pivaloylacetonitrile (1.2 equiv.) was stirred overnight at 80 °C in ethanol. The solution was extracted with EtOAc. The organic layer was washed with water and brine, dried (MgSO_4_), filtered, and evaporated to dryness.

*3-(tert-Butyl)-1-(p-tolyl)-1H-pyrazol-5-amine* (**2a**). From *p*-tolylhydrazine hydrochloride (1.59 g, 10 mmol) and pivaloylacetonitrile (1.5 g, 12 mmol), after work-up and without further purification, compound **2a** was obtained as a white solid (1.8 g, 80%). IR (KBr): 3345, 1598, 1578 cm^−1^; ^1^H-NMR (CDCl_3_) δ: 7.40 (d, 2H, *J* = 8.8 Hz), 7.25 (d, 2H, *J* = 8.8 Hz), 5.50 (s, 1H), 3.72 (brs, 2H, NH_2_), 2.37 (s, 3H), 1.32 (s, 9H). MS (ESI): *m*/*z* 252.16 [M + Na]^+^. Mp 121.3–123.4 °C.

*3-(tert-Butyl)-1-phenyl-1H-pyrazol-5-amine* (**2b**). Yellow solid (1.74 g, 81%). IR (KBr): 3340, 1601, 1575 cm^−1^; ^1^H-NMR (CDCl_3_) δ: 7.60–7.69 (m, 2H), 7.50–7.57 (m, 2H), 7.36–7.41 (m, 1H), 5.56 (s, 1H), 3.79 (brs, 2H, NH_2_), 1.30 (s, 9H). MS (ESI): *m*/*z* 238.14 [M + Na]^+^. Mp 118.1–120.0 °C.

*3-(tert-Butyl)-1-(m-tolyl)-1H-pyrazol-5-amine* (**2c**). White solid (2.06 g, 90%). IR (KBr): 3343, 1600, 1579 cm^−1^; ^1^H-NMR (CDCl_3_) δ: 7.60–7.68 (m, 1H), 7.40–7.48 (m, 1H), 7.35–7.38 (m, 1H), 7.29–7.33 (m, 1H), 5.56 (s, 1H), 3.75 (brs, 2H, NH_2_), 2.37 (s, 3H), 1.30 (s, 9H). MS (ESI): *m*/*z* 252.16 [M + Na]^+^. Mp 120.3–122.4 °C.

*3-(tert-Butyl)-1-(4-methoxyphenyl)-1H-pyrazol-5-amine* (**2d**). White solid (1.97 g, 80%). IR (KBr): 3341, 1593, 1574 cm^−1^; ^1^H-NMR (CDCl_3_) δ: 7.41 (d, 2H), 6.97 (d, 2H), 5.43 (s, 1H), 3.83 (s, 3H), 3.72 (brs, 2H, NH_2_), 1.32 (s, 9H). MS (ESI): *m*/*z* 268.11 [M + Na]^+^. Mp 109.1–111.3 °C.

*3-(tert-Butyl)-1-(4-chlorophenyl)-1H-pyrazol-5-amine* (**2e**). White solid (1.87 g, 75%). IR (KBr): 3340, 1597, 1581 cm^−1^; ^1^H-NMR (CDCl_3_) δ: 7.59 (d, 2H, *J* = 8.8 Hz), 7.10 (d, 2H, *J* = 8.8 Hz), 5.47 (s, 1H), 3.74 (brs, 2H, NH_2_), 1.32 (s, 9H). MS (ESI): *m*/*z* 272.10 [M + Na]^+^. Mp 107.3–109.5 °C.

*1-(4-Bromophenyl)-3-(tert-butyl)-1H-pyrazol-5-amine* (**2f**). White solid (2.50 g, 85%). IR (KBr): 3337, 1596, 1575 cm^−1^; ^1^H-NMR (CDCl_3_) δ: 7.54 (d, 2H, *J* = 8.4 Hz), 7.14 (d, 2H, *J* = 8.4 Hz), 5.42 (s, 1H), 3.71 (brs, 2H, NH_2_), 1.31 (s, 9H). MS (ESI): *m*/*z* 316.05 [M + Na]^+^. Mp 109.1–112.7 °C.

*3-(tert-Butyl)-1-(4-fluorophenyl)-1H-pyrazol-5-amine* (**2g**). *W*hite solid (1.86 g, 79%). IR (KBr): 3333, 1594, 1582 cm^−1^; ^1^H-NMR (CDCl_3_) δ: 7.59 (d, 2H, *J* = 8.8 Hz), 7.10 (d, 2H, *J* = 8.8 Hz), 5.58 (s, 1H), 3.62 (brs, 2H, NH_2_), 1.31 (s, 9H). MS (ESI): *m*/*z* 256.34 [M + Na]^+^. Mp 102.3–104.6 °C.

*3-(tert-Butyl)-1-(4-nitrophenyl)-1H-pyrazol-5-amine* (**2h**). White solid (1.69 g, 65%). IR (KBr): 3333, 1594, 1582 cm^−1^; ^1^H-NMR (CDCl_3_) δ: 8.28 (d, 2H, *J* = 8.8 Hz), 7.93 (d, 2H, *J* = 8.8 Hz), 5.55 (s, 1H), 3.69 (brs, 2H, NH_2_), 1.30 (s, 9H). MS (ESI): *m*/*z* 283.13 [M + Na]^+^. Mp 105.1–107.3 °C.

*4-(5-Amino-3-(tert-butyl)-1H-pyrazol-1-yl)benzenesulfonamide* (**2i**). White solid (2.35 g, 80%). IR (KBr): 3367, 3342, 1592, 1588 cm^−1^; ^1^H-NMR (DMSO-*d*_6_) δ: 8.11 (d, 2H, *J* = 8.8 Hz), 7.83 (d, 2H, *J* = 8.8 Hz), 7.47 (s, 2H), 5.51 (s, 1H), 3.73 (brs, 2H, NH_2_), 1.32 (s, 9H).MS (ESI): *m*/*z* 317.12 [M + Na]^+^. Mp 103.1–105.2 °C.

#### 3.2.2. General Method for the Synthesis of **3a**–**3i**

A solution of the corresponding **2a**–**2i** (1 equiv.) and 2,2,2-trichloroethyl carbonochloridate (1.1 equiv.) in THF was stirred 6 h at 0 °C, then the mixture was filtered and extracted three times with ethyl acetate. The solution was dried with MgSO_4_ and concentrated to dryness.

*2,2,2-Trichloroethyl(3-(tert-butyl)-1-(p-tolyl)-1H-pyrazol-5-yl)carbamate* (**3a**). From **2a** (2.30 g, 10 mmol) and 2,2,2-trichloroethyl carbonochloridate (2.3 g, 11 mmol), after work-up and without further purification, **3a** was obtained as a white solid (3.02 g, 75%). IR (KBr): 3169, 1597, 1580 cm^−1^; ^1^H-NMR (DMSO-*d*_6_) δ: 9.47 (s, 1H), 7.41 (d, 2H, *J* = 8.8 Hz), 7.23 (d, 2H, *J* = 8.8 Hz), 5.51 (s, 1H), 4.85(s, 2H), 1.32 (s, 9H). MS (ESI): *m*/*z* 426.06 [M + Na]^+^. Mp 153.3–155.7 °C.

*2,2,2-Trichloroethyl(3-(tert-butyl)-1-phenyl-1H-pyrazol-5-yl)carbamate* (**3b**). White solid (3.50 g, 90%). IR (KBr): 3170, 1593, 1577 cm^−1^; ^1^H-NMR (DMSO-*d*_6_) δ: 9.47(s, 1H), 7.65 (m, 2H), 7.57 (m, 2H), 7.40 (m, 1H), 5.52 (s, 1H), 4.85 (s, 2H), 1.32 (s, 9H). MS (ESI): *m*/*z* 412.11 [M + Na]^+^. Mp 151.1–153.2 °C.

*2,2,2-Trichloroethyl(3-(tert-butyl)-1-(m-tolyl)-1H-pyrazol-5-yl)carbamate* (**3c**). White solid (3.62 g, 90%). IR (KBr): 3168, 1601, 1582 cm^−1^; ^1^H-NMR (DMSO-*d*_6_) δ: 9.43 (s, 1H), 7.60–7.69 (m, 1H), 7.39–7.44 (m, 1H), 7.35–7.38 (m, 1H), 7.29–7.33 (m, 1H), 5.49 (s, 1H), 4.88 (s, 2H), 2.37 (s, 3H), 1.30 (s, 9H). MS (ESI): *m*/*z* 426.06 [M + Na]^+^. Mp 148.3–150.2 °C.

*2,2,2-Trichloroethyl(3-(tert-butyl)-1-(4-methoxyphenyl)-1H-pyrazol-5-yl)carbamate* (**3d**). White solid (3.56 g, 85%). IR (KBr): 3170, 1600, 1578 cm^−1^; ^1^H-NMR (DMSO-*d*_6_) δ: 9.40 (s, 1H), 7.41 (d, 2H, *J* = 8.8 Hz), 6.97 (d, 2H, *J* = 8.8 Hz), 5.44 (s, 1H), 4.81(s, 2H), 1.30 (s, 9H). MS (ESI): *m*/*z* 442.09 [M + Na]^+^. Mp 150.1–152.2 °C.

*2,2,2-Trichloroethyl(3-(tert-butyl)-1-(4-chlorophenyl)-1H-pyrazol-5-yl)carbamate* (**3e**). White solid (3.60 g, 85%). IR (KBr): 3166, 1600, 1577 cm^−1^; ^1^H-NMR (DMSO-*d*_6_) δ: 9.42 (s, 1H), 7.57 (d, 2H, *J* = 8.8 Hz), 7.12 (d, 2H, *J* = 8.8 Hz), 5.41 (s, 1H), 4.80 (s, 2H), 1.32 (s, 9H). MS (ESI): *m*/*z* 446.01 [M + Na]^+^. Mp 147.2–149.3 °C.

*2,2,2-Trichloroethyl(1-(4-bromophenyl)-3-(tert-butyl)-1H-pyrazol-5-yl)carbamate* (**3f**). White solid (3.54 g, 76%). IR (KBr): 3169, 1602, 1579 cm^−1^; ^1^H-NMR (DMSO-*d*_6_) δ: 9.42 (s, 1H), 7.54 (d, 2H, *J* = 8.8 Hz), 7.16 (d, 2H, *J* = 8.8 Hz), 5.37 (s, 1H), 4.78 (s, 2H), 1.30 (s, 9H). MS (ESI): *m*/*z* 490.00 [M + Na]^+^. Mp 145.1–147.6 °C.

*2,2,2-Trichloroethyl(3-(tert-butyl)-1-(4-fluorophenyl)-1H-pyrazol-5-yl)carbamate* (**3g**). White solid (3.25 g, 80%). IR (KBr): 3166, 1593, 1570 cm^−1^; ^1^H-NMR (DMSO-*d*_6_) δ: 9.40 (s, 1H), 7.48 (d, 2H, *J* = 8.8 Hz), 7.18 (d, 2H, *J* = 8.8 Hz), 5.33 (s, 1H), 4.64 (s, 2H), 1.29 (s, 9H). MS (ESI): *m*/*z* 430.04 [M + Na]^+^. Mp 148.7–150.1 °C.

*2,2,2-Trichloroethyl(3-(tert-butyl)-1-(4-nitrophenyl)-1H-pyrazol-5-yl)carbamate* (**3h**). Yellow solid (3.47 g, 80%). IR (KBr): 3165, 1595, 1573 cm^−1^; ^1^H-NMR (DMSO-*d*_6_) δ: 9.39 (s, 1H), 8.28 (d, 2H, *J* = 8.8 Hz), 7.93 (d, 2H, *J* = 8.8 Hz), 5.37 (s, 1H), 4.73 (s, 2H), 1.30 (s, 9H). MS (ESI): *m*/*z* 457.13 [M + Na]^+^. Mp 149.3–151.2 °C.

*2,2,2-Trichloroethyl(3-(tert-butyl)-1-(4-sulfamoylphenyl)-1H-pyrazol-5-yl)carbamate* (**3i**). White solid (3.27 g, 70%). IR (KBr): 3367, 3160, 1599, 1580 cm^−1^; ^1^H-NMR (DMSO-*d*_6_) δ: 9.39 (s, 1H), 8.15 (d, 2H, *J* = 8.8 Hz), 7.80 (d, 2H, *J* = 8.8 Hz), 7.45 (s, 2H), 5.35 (s, 1H), 4.70 (s, 2H), 1.29 (s, 9H); MS (ESI): *m*/*z* 491.02 [M + Na]^+^. Mp 143.4–145.6 °C.

#### 3.2.3. General Method for the Synthesis of **5a**–**5b**

A solution of the corresponding **4a**–**4b** (1 equiv.) and 4-amino-3-methylphenol (10 mmol) was stirred 2 h at 90 °C in the presence of K_2_CO_3_ (4.14g 30 mmol) in DMSO (50 mL). Then the solution was extracted with EtOAc. The organic layer was washed with water and brine, dried (MgSO_4_), filtered and evaporated to dryness. The product was separated by column chromatography using PE/EA (3:1) as eluent.

*2-(4-Amino-3-methylphenoxy)benzonitrile* (**5a**). From **4a** (1.21 g, 10 mmol), and 4-amino-3-methylphenol (1.23 g, 10 mmol), after work-up and purification, **5a** was obtained as a white solid. (1.56 g, 70%). IR (KBr): 3275, 2250, 1595, 1575 cm^−1^; ^1^H-NMR (DMSO-*d*_6_) δ: 8.10–8.16 (m, 1H), 8.00–8.06 (m, 1H), 7.77–7.81 (m, 1H), 7.39–7.42 (m, 1H), 7.29–7.32 (m, 1H), 7.22–7.27 (m, 1H), 7.09–7.11 (m, 1H), 4.39 (s, 2H, NH_2_), 2.55 (s, 3H); MS (ESI): *m*/*z* 447.09 [M + Na]^+^. Mp 191.3–193.7 °C.

*2-(4-Amino-3-methylphenoxy)-5-fluorobenzonitrile* (**5b**). White solid (1.81 g, 75%). IR (KBr): 3274, 2250, 1593, 1574 cm^−1^; ^1^H-NMR (DMSO-*d*_6_) δ: 8.10–8.16 (m, 1H), 8.00–8.06 (m, 1H), 7.74–7.78 (m, 1H), 7.39–7.42 (m, 1H), 7.19–7.24 (m, 1H), 7.10–7.14 (m, 1H), 4.38 (s, 2H, NH_2_), 2.54 (s, 3H); MS (ESI): *m*/*z* 465.25 [M + Na]^+^. Mp 191.3–193.7 °C.

#### 3.2.4. General Method for the Synthesis of **6a**–**6b**

A solution of the corresponding **5a**–**5b** (1 equiv.) and isoamyl nitrite (1.5 equiv.) was stirred 8 h at 90 °C in the presence of potassium acetate (0.98 g, 10 mmol) and acetic anhydride (1.75 g, 15 mmol) in toluene (50 mL). The solution was extracted with EtOAc. The organic layer was washed with water and brine, dried (MgSO_4_), filtered, and evaporated to dryness. The product was separated by column chromatography using PE/EA (8:1) as eluent.

*2-((1-Acetyl-1H-indazol-5-yl)oxy)benzonitrile* (**6a**). From **5a** (2.24 g, 10 mmol), acetic anhydride (3 g, 30 mmol), potassium acetate (0.98 g, 10 mmol) and isoamyl nitrite (1.75 g, 15 mmol), after work-up and purification, **6a** was obtained as a white solid. (1.66 g, 60%). IR (KBr): 2250, 1698, 1593, 1574 cm^−1^; ^1^H-NMR (DMSO-*d*_6_) δ: 8.46 (s, 1H), 8.34–8.39 (m, 1H), 7.90–7.95 (m, 1H), 7.66–7.69 (m, 1H), 7.61–7.65 (m, 1H), 7.44–7.51 (m, 1H), 7.27–7.32 (m, 1H), 6.92–6.99 (m, 1H), 2.74 (s, 3H); MS (ESI): *m*/*z* 310.09 [M + Na]^+^. Mp 201.7–203.1 °C.

*2-((1-Acetyl-1H-indazol-5-yl)oxy)-5-fluorobenzonitrile* (**6b**). White solid (1.62 g, 55%). IR (KBr): 2250, 1696, 1599, 1579 cm^−1^; ^1^H-NMR (DMSO-*d*_6_) δ: 8.49 (s, 1H), 8.29–8.36 (m, 1H), 7.87–7.92 (m, 1H), 7.60–7.65 (m, 1H), 7.50–7.57 (m, 1H), 7.40–7.46 (m, 1H), 6.91–6.99 (m, 1H), 2.76 (s, 3H); MS (ESI): *m*/*z* 318.22 [M + Na]^+^. Mp 205.1–207.3 °C.

#### 3.2.5. General Method for the Synthesis of **7a**–**7b**

A solution of the corresponding **6a** or **6b** (1 equiv.) and LiAlH_4_ (3 equiv.) in THF was stirred 1 h at 65 °C. Then the solution was extracted with EtOAc. The organic layer was washed with water and brine, dried (MgSO_4_), filtered and evaporated to dryness. Products were purified by column chromatography using DCM/MeOH (50:1) as eluent.

*(2-((1H-Indazol-5-yl)oxy)phenyl)methanamine* (**7a**). From **6a** (2.77 g, 10 mmol) and LiAlH_4_ (1.14 g, 15 mmol), after work-up and purification, **7a** was obtained as a solid a white solid. (1.20 g, 50%). IR (KBr): 3277, 1603, 1579 cm^−1^; ^1^H-NMR (DMSO-*d*_6_) δ: 13.12 (s, 1H), 8.08 (s, 1H), 7.84–7.91 (m, 1H), 7.60–7.65 (m, 3H), 7.23–7.28 (m, 2H), 6.81–6.87 (m, 1H), 4.23–4.25 (t, 2H), 3.79–3.83 (m, 2H); MS (ESI): *m*/*z* 262.11 [M + Na]^+^. Mp 178.3–180.5 °C.

*(2-((1H-Indazol-5-yl)oxy)-5-fluorophenyl)methanamine* (**7b**) White solid solid (1.41 g, 55%). IR (KBr): 3279, 1599, 1576 cm^−1^; ^1^H-NMR (DMSO-*d*_6_) δ: 13.11 (s, 1H), 8.08 (s, 1H), 7.84–7.89 (m, 1H), 7.59–7.65 (m, 2H), 7.21–7.25 (m, 2H), 6.81–6.88 (m, 1H), 4.25–4.27 (t, 2H), 3.80–3.86 (m, 2H); MS (ESI): *m*/*z* 280.26 [M + Na]^+^. Mp 180.1–182.7 °C.

#### 3.2.6. Synthesis of **14**

*4-Fluoro-1-nitro-2-(prop-2-yn-1-yloxy)benzene* (**9**). Compound **8** (6.7 g, 40 mmol) was dissolved in DMF (60 mL), and K_2_CO_3_ (8.3 g, 60 mmol) was added while stirring vigorously. 3-Bromoprop-1-yne (50 mmol, 5.9 g) was then added dropwise while the mixture was on ice, and the reaction was allowed to proceed at room temperature for 12 h. The mixture was poured into ice water and kept in fridge for a day. The yellow solid **9** that appeared was filtered off and dried (yield 97%). IR (KBr): 3309, 2160, 1593, 1573 cm^−1^; ^1^H-NMR (DMSO-*d*_6_) δ: 8.00–8.06 (m, 1H), 7.31–7.37 (m, 1H), 7.01–7.07 (m, 1H), 5.06 (s, 2H), 3.75 (s, 1H); MS (ESI): *m*/*z* 218.15 [M + Na]^+^. Mp 110.4–112.7 °C.

*2-Methyl-1-nitro-4-(4-nitro-3-(prop-2-yn-1-yloxy)phenoxy)benzene* (**10**). To DMF (40 mL) compound **9** (1.95 g, 10 mmol) and K_2_CO_3_ (4.0 g, 30 mmol) were added and the mixture was stirred at room temperature for 4 h, followed by the addition of 4-amino-3-methylphenol (1.23 g, 10 mmol). The suspension formed was stirred at 100 °C for 3 h. The reaction mixture was cooled, and poured into ice water (100 mL), then the product was briefly placed in a fridge whereby a white solid appeared. The product was recovered by suction filtration and the filter cake was dried to give 2.55 g (90%) of compound **10**. IR (KBr): 3309, 3267, 2160, 1593, 1573 cm^−1^; ^1^H-NMR (DMSO-*d*_6_) δ: 8.00–8.08 (m, 1H), 7.30–7.37 (m, 1H), 7.20–7.26 (m, 1H), 7.10–7.15 (m, 1H), 7.00–7.07 (m, 1H), 6.08–6.12 (m, 1H), 5.06 (s, 2H), 3.75 (s, 1H), 2.95 (s, 3H); MS (ESI): *m*/*z* 321.10 [M + Na]^+^. Mp 150.1–152.4 °C.

*1-(5-(3-(Ethynyloxy)-4-nitrophenoxy)-1H-indazol-1-yl)ethanone* (**11**). Compound **10** (0.8 g, 2.7 mmol) and acetic anhydride (1.14 g) were added to dry toluene (50 mL), followed by potassium acetate (0.3 g). The suspension was stirred at 80 °C and then isopentyl nitrite (0.6 g, 5 mmol) was added. The reaction was continued for 12 h, then the suspension was cooled down and filtered. Product **11** can be purified by column chromatography using DCM/MeOH (100: 1) as eluent, yield (75%). IR (KBr): 3309, 2160, 1670, 1593, 1573 cm^−1^; ^1^H-NMR (DMSO-*d*_6_) δ: 8.04 (s, 1H), 7.90–7.94 (m, 1H), 7.40–7.45 (m, 1H), 7.21–7.26 (m, 1H), 7.11–7.14 (m, 1H), 7.06–7.11 (m, 1H), 6.10–6.15 (m, 1H), 5.06 (s, 2H), 3.75 (s, 1H), 2.25 (s, 3H); MS (ESI): *m*/*z* 374.09 [M + Na]^+^. Mp 169.7–171.3 °C.

*4-((1H-Indazol-5-yl)oxy)-2-(ethynyloxy)aniline* (**12**). Compound **11** (2.0 g, 6 mmol) was placed in a 100 mL flask and then dissolved in ethyl acetate (40 mL). Ethanol (40 mL) was then added, followed by SnCl_2_ (4.5 g, 21 mmol). The mixture was heated to reflux for 6 h. Next saturated NaHCO_3_ solution was added until bubbles ceased to come out, and the mixture was filtered. The filtrate was extracted with ethyl acetate and then dried and then separated by column chromatography using DCM/MeOH (100:1) as eluent, yield (54%). IR (KBr): 3309, 3277, 2160, 1593, 1573 cm^−1^; ^1^H-NMR (DMSO-*d*_6_) δ: 13.01 (s, 1H), 8.05 (s, 1H), 7.90–7.99 (m, 1H), 7.40–7.45 (m, 1H), 7.21–7.24 (m, 1H), 7.11–7.15 (m, 1H), 7.02–7.12 (m, 1H), 6.11–6.14 (m, 1H), 5.04 (s, 2H), 3.77 (s, 1H), 3.28 (s, 2H); MS (ESI): *m*/*z* 302.09 [M + Na]^+^. Mp 192.1–194.7 °C.

*tert-Butyl(4-((1H-indazol-5-yl)oxy)-2-(ethynyloxy)phenyl)carbamate* (**13**). Compound **12** (2 g, 7 mmol) was dissolved in CH_2_Cl_2_ (25 mL) in a 100 mL flask. The flask was placed in a low temperature reaction tank and Et_3_N (3 mL) was added while the temperature was kept under −10 °C. Then (Boc)_2_O (3.9 g, 0.017 mol) was added to the flask in portions. The reaction was carried out for 48 h, then the solution was washed with water, dried, then separated by column chromatography using DCM/MeOH (50:1) as eluent, yield (63%). IR (KBr): 3309, 3250, 2160, 1690, 1593, 1573 cm^−1^; ^1^H-NMR (DMSO-*d*_6_) δ: 13.01 (s, 1H), 8.01 (s, 1H), 7.92–7.99 (m, 1H), 7.52–7.57 (m, 2H), 7.30–7.35 (m, 1H), 7.11–7.15 (m, 2H), 6.83–6.88 (m, 1H), 6.48–6.53 (m, 1H), 4.80 (s, 2H), 3.61 (s, 1H), 2.30 (s, 1H), 1.45 (s, 9H); MS (ESI): *m*/*z* 412.15 [M + Na]^+^. Mp 177.4–179.1 °C.

*5-((1H-Indazol-5-yl)oxy)-2H-chromen-8-amine* (**14**). Compound **13** (1.6 g, 0.004 mol) was dissolved in diphenyl ether (20 mL) in a 50 mL flask. The method using microwave was carried out as follows: temperature: 220 °C, pressure: 8.0 bar, time: 30 min, power: 250 w. Then the oxydibenzene was removed through vacuum distillation. Product **14** was then separated by column chromatography using DCM/MeOH (80:1) as eluent, yield (25%). IR (KBr): 3310, 3245, 1680, 1593, 1573 cm^−1^; ^1^H-NMR (DMSO-*d*_6_) δ: 12.99 (s, 1H), 7.92 (s, 1H), 7.48–7.52 (m, 1H), 7.05–7.09 (m, 1H), 6.98–7.02 (m, 1H), 6.45–6.49 (m, 1H), 6.40–6.43 (m, 1H), 6.30–6.34 (m, 1H), 5.80–5.83 (m, 1H), 4.73–4.76 (m, 2H), 4.56 (s, 2H); MS (ESI): *m*/*z* 312.15 [M + Na]^+^. Mp 181.3–183.7 °C.

#### 3.2.7. Synthesis of **16**

*2-(2-Morpholinoethoxy)benzonitrile* (**15**). 2-Morpholinoethanol (4.2 g) was added to DMF (50 mL) in a 250 mL flask, followed by addition of sodium hydride (3 g) at −35 °C. An amount of **4a** (3.6 g) was dissolved in DMF (15 mL). This solution was added dropwise to the flask and the mixture was stirred at −35 °C for 12 h. The mixture was poured into ice water (200 mL), and the solution was extracted with ethyl acetate. A gray oil was collected after drying and concentrating; yield 64%. IR (KBr): 2250, 1598, 1578 cm^−1^; ^1^H-NMR (DMSO-*d*_6_) δ: 7.22–7.25 (m, 1H), 7.13–7.16 (m, 1H), 6.85–6.88 (m, 1H), 6.70–6.74 (m, 1H), 4.07–4.09 (t, 2H, *J* = 5.6), 3.57–3.59 (t, 4H, *J* = 4.8), 2.72–2.74 (t, 2H, *J* = 5.6), 2.44–2.46 (t, 4H, *J* = 4.8); MS (ESI): *m*/*z* 255.12 [M + Na]^+^.

*(2-(2-Morpholinoethoxy)phenyl)methanamine* (**16**). LiAlH_4_ (1.5 g) was added to anhydrous THF (40 mL) in a 150 mL flask, which was kept in an ice bath. Compound **15** (2.32 g, 10 mmol) was dissolved in anhydrous THF (15 mL) and this solution was added dropwise to LiAlH_4_ suspension and the mixture then stirred at 60 °C for 2 h. Ethanol was added to the mixture until gas evolution stopped, then the suspension was filtered and concentrated. The product **16** was obtained as a solid by column chromatography (DCM/MeOH (100:1)), yield (64%). IR (KBr): 3270, 1597, 1577 cm^−1^; ^1^H-NMR (DMSO-*d*_6_) δ: 7.23–7.27 (m, 1H), 7.16–7.18 (m, 1H), 6.93–6.99 (m, 1H), 6.80–6.87 (m, 1H), 4.28–4.30 (t, 2H, *J* = 7.6), 4.08–4.10 (t, 2H, *J* = 5.6), 3.56–3.58 (t, 4H, *J* = 4.8), 2.70–2.72 (t, 2H, *J* = 5.6), 2.46–2.48 (t, 4H, *J* = 4.8); MS (ESI): *m*/*z* 259.12 [M + Na]^+^. Mp 181.3–183.7 °C.

#### 3.2.8. General Method for the Synthesis of **18a** and **18b**

*2-((5-Oxo-5,6,7,8-tetrahydronaphthalen-2-yl)oxy)benzonitrile* (**17a**). 6-Hydroxy-1-tetralone (5 g, 30.86 mmol) was placed in a 250 mL three-necked flask and dissolved in DMSO (100 mL). The flask was heated to 90 °C, and K_2_CO_3_ (12.78 g) was added under stirring. Compound **4a** (4.48 g, 37.03 mmol) was added dropwise into the flask and the reaction mixture was heated to 90 °C for 4 h. The mixture was poured into water (200 mL), and the water phase was extracted twice with ethyl acetate. The organic phases were combined, washed with water and brine, dried with Na_2_SO_4_, and the solvent removed under vacuum to yield the crude product, which was purified by column chromatography using petroleum ether/ethyl acetate (5:1) as eluent to give compound **17a** as a yellow solid (75.3%). IR (KBr): 2253, 1688, 1597, 1577 cm^−1^; ^1^H-NMR (CDCl_3_), δ: 8.32–8.35 (m, 1H), 7.66–7.69 (m, 1H), 7.59–7.61 (m, 1H), 7.35–7.38 (m, 3H), 7.00–7.04 (m, 1H), 2.90–2.94 (m, 2H), 2.71–2.74 (m, 2H), 2.00–2.03 (m, 2H). MS (ESI): *m*/*z* 286.34 [M + Na]^+^. Mp 178.3–180.5 °C.

*5-Fluoro-2-((5-oxo-5,6,7,8-tetrahydronaphthalen-2-yl)oxy)benzonitrile* (**17b**). White solid, yield 71.1%. IR (KBr): 2250, 1687, 1597, 1577 cm^−1^; ^1^H-NMR (CDCl_3_), δ: 8.40–8.43 (m, 1H), 7.59–7.62 (m, 1H), 7.35–7.39 (m, 2H), 7.19–7.24 (m, 1H), 7.07–7.11 (m, 1H), 2.93–2.96 (m, 2H), 2.73–2.77 (m, 2H), 2.01–2.05 (m, 2H). MS (ESI): *m*/*z* 304.22 [M + Na]^+^. Mp 171.7–173.2 °C.

*6-(2-(Aminomethyl)phenoxy)-1,2,3,4-tetrahydronaphthalen-1-ol* (**18a**). LiAlH_4_ (0.35 g, 9 mmol) was added to anhydrous THF (15 mL) in a 50 mL flask, which was placed in an ice bath. Compound **17a** (0.74 g, 3 mmol) was dissolved in anhydrous THF (15 mL) and this solution was added dropwise into the LiAlH_4_ suspension and the mixture then stirred at 65 °C for 30–40 min. Ethanol was added until bubble evolution ceased, then the suspension was filtered and concentrated. Compound **18a** was separated by column chromatography (dichloromethane/methanol 25:1), yield 77.7%. IR (KBr): 3350, 1597, 1577 cm^−1^; ^1^H-NMR (CDCl_3_), δ: 7.20–7.25 (m, 1H), 7.14–7.17 (m, 1H), 7.05–7.09 (m, 2H), 7.00–7.02 (m, 1H), 6.87–6.93 (m, 1H), 6.86–6.90 (m, 1H), 4.55–4.60 (m, 1H), 4.20–4.22 (t, 2H, *J* = 7.6 Hz), 2.90–2.94 (m, 2H), 2.73–2.76 (m, 2H), 2.01–2.04 (m, 2H). MS (ESI): *m*/*z* 292.01 [M + Na]^+^. Mp 165.2–167.1 °C.

*6-(2-(Aminomethyl)-4-fluorophenoxy)-1,2,3,4-tetrahydronaphthalen-1-ol* (**18b**). White solid, yield 77.7%. IR (KBr): 3347, 1595, 1575 cm^−1^; ^1^H-NMR (CDCl_3_), δ: 7.21–7.24 (m, 1H), 7.16–7.20 (m, 1H), 7.08–7.12 (m, 2H), 6.89–6.92 (m, 1H), 6.85–6.89 (m, 1H), 4.54–4.59 (m, 1H), 4.19–4.21 (t, 2H, *J* = 7.6 Hz), 2.95–2.99 (m, 2H), 2.75–2.80 (m, 2H), 2.04–2.09 (m, 2H). MS (ESI): *m*/*z* 310.13 [M + Na]^+^. Mp 166.6–168.3 °C.

#### 3.2.9. Synthesis of **21**

*4-(2-(2-Nitrophenoxy)ethyl)morpholine* (**20**). 2-Morpholinoethanol (1.3 g,10 mmol) was added to DMF (30 mL) in a 50-mL flask, followed by addition of sodium hydride (0.48 g, 20 mmol) at −35 °C. An amount of 1-fluoro-2-nitrobenzene (**19**, 3.26 g, 12 mmol) was dissolved in DMF (15 mL) and the solution was added dropwise to the flask and the reaction mixture stirred at −35 °C for 12 h. The mixture was poured into ice water (200 mL), and extracted with ethyl acetate. A gray oil was collected after drying and concentrating; yield 64%. IR (KBr): 1598, 1578 cm^−1^; ^1^H-NMR (DMSO-*d*_6_) δ: 8.29–8.32 (m, 1H), 7.87–7.89 (m, 1H), 7.57–7.61 (m, 1H), 7.43–7.48 (m, 1H), 4.07–4.09 (t, 2H, *J* = 5.6 Hz), 3.59–3.61 (t, 4H, *J* = 4.8 Hz), 2.76–2.78 (t, 2H, *J* = 5.6 Hz), 2.46–2.48 (t, 4H, *J* = 4.8 Hz); MS (ESI): *m*/*z* 275.12 [M + Na]^+^.

*2-(2-Morpholinoethoxy)aniline* (**21**). Compound **20** (2.52 g, 10 mmol) was added to EtOH (50 mL) in a 100-mL flask, followed by SnCl_2_·2H_2_O (11.28 g (50 mmol) and the mixture was heated at 80 °C for 2 h. The mixture was then poured into 10% aqueous NaOH solution (200 mL), and the solid was filtered. The water phase was extracted twice with ethyl acetate, and the organic phases were combined and washed with water and brine and dried with Na_2_SO_4_. The crude product was obtained after concentration; yield 60%. IR (KBr): 3267, 1597, 1577 cm^−1^; ^1^H-NMR (DMSO-*d*_6_) δ: 8.29–8.32 (m, 1H), 7.87–7.89 (m, 1H), 7.57–7.61 (m, 1H), 7.43–7.48 (m, 1H), 6.12 (s, 1H) 4.05–4.07 (t, 2H, *J* = 5.6 Hz), 3.58–3.60 (t, 4H, *J* = 4.8 Hz), 2.73–2.75 (t, 2H, *J* = 5.6 Hz), 2.49–2.51 (t, 4H, *J* = 4.8 Hz); MS (ESI): *m*/*z* 245.12 [M + Na]^+^. Mp 165.3–167.7 °C.

#### 3.2.10. Synthesis of **24**

*2-Methyl-4-(2-nitrophenoxy)aniline* (**22**). Compound **19** (1.41 g, 10 mmol) and 4-amino-3-methylphenol (1.23 g, 10 mmol) were stirred 2 h at 90 °C in the presence of K_2_CO_3_ (4.14g 30 mmol) in DMSO (50 mL). The mixture was poured into ice water (200 mL), and the solution was extracted with ethyl acetate. The organic layer was washed with water and brine, dried (MgSO_4_), filtered, and evaporated to dryness. After work-up and purification by column chromatography using PE/EA (3:1) as eluent, intermediate **20** was obtained as a white solid; yield 74%. IR (KBr): 3275, 1595, 1575 cm^−1^; ^1^H-NMR (DMSO-*d*_6_) δ: 8.17–8.21 (m, 1H), 8.01–8.04 (m, 1H), 7.72–7.76 (m, 1H), 7.44–7.47 (m, 1H), 7.33–7.36 (m, 1H), 7.27–7.31 (m, 1H), 7.12–7.15 (m, 1H), 4.41(s, 2H, NH_2_), 2.53(s, 3H); MS (ESI): *m*/*z* 267.08 [M + Na]^+^. Mp 153.1–155.5 °C.

*1-(5-(2-Nitrophenoxy)-1H-indazol-1-yl) ethanone* (**23**). Compound **22** (2.44 g, 10 mmol, and acetic anhydride (3.06 g, 30 mmol) were added to dry toluene (50 mL) followed by potassium acetate (0.98 g, 10 mmol). The suspension was stirred at 80 °C, and isopentyl nitrite (1.76 g, 15 mmol) was added. The reaction was continued for 18 h. The suspension was cooled and filtered. The product was separated by column chromatography using PE/EA (10:1) as an eluent; yield 60%. IR (KBr): 1696, 1594, 1573 cm^−1^; ^1^H-NMR (DMSO-*d*_6_) δ: 8.43–8.45 (s, 1H), 8.31–8.34 (m, 1H), 7.90–7.92 (m, 1H), 7.64–7.67 (m, 1H), 7.63–7.66 (m, 1H), 7.55–7.58 (m, 1H), 7.33–7.36 (m, 1H), 6.99 (m, 1H), 2.76 (s, 3H); MS (ESI): *m*/*z* 320.07 [M + Na]^+^. Mp 167.4–169.6 °C.

*2-((1H-Indazol-5-yl)oxy)aniline* (**24**). Compound **23** (2.97 g, 10 mmol) was added to EtOH (50 mL) in a 100-mL flask, followed by SnCl_2_·2H_2_O (11.30 g, 50 mmol) and the mixture was reacted at 80 °C for 2 h. The mixture was poured into 10% aqueous NaOH solution (200 mL), and the solid formed was filtered. The water phase was extracted twice with ethyl acetate, and the organic phases were combined, washed with water and brine, and dried with Na_2_SO_4_. A yellow solid was collected after concentration; yield 57%. IR (KBr): 3272, 1600, 1578 cm^−1^; ^1^H-NMR (DMSO-*d*_6_) δ: 13.11 (s, 1H), 8.04 (s, 1H), 7.84–7.89 (m, 1H), 7.63–7.66 (m, 3H), 7.20–7.24 (m, 2H), 6.86–6.89 (m, 1H), 3.80–3.82 (m, 2H); MS (ESI): *m*/*z* 248.09 [M + Na]^+^. Mp 180.1–182.4 °C.

#### 3.2.11. General Method for the Synthesis of **25a**–**25k**

A solution of the corresponding **3a**–**3i** (1.1 equiv.) and **7a**–**7b** (1 equiv.) was stirred for 2 h at 90 °C in the presence of Et_3_N (1 mL) in DMSO (50 mL). The mixture was poured into ice water, and the solution was extracted with EtOAc. The organic layer was washed with water and brine, dried (MgSO_4_), filtered and evaporated to dryness. The product was separated by column chromatography using DCM/MeOH (100:1) as an eluent.

*1-(2-((1H-Indazol-5-yl)oxy)benzyl)-3-(3-(tert-butyl)-1-(p-tolyl)-1H-pyrazol-5-yl)urea* (**25a**). From **3a** (4.03 g, 10 mmol), and **7a** (2.39 g, 10 mmol), after work-up and purification **25a** (3.46 g, 70%) was obtained as a white solid. HPLC purity 98.1% (*t*_R_ =11.97 min). IR (KBr): 3257, 1670, 1594, 1576 cm^−1^; ^1^H-NMR (DMSO-*d*_6_) δ: 13.13 (s, 1H), 8.22 (s, 1H), 8.01 (s, 1H), 7.58 (d, 1H, *J* = 8.8 Hz), 7.37–7.40 (m, 2H), 7.25–7.27 (m, 3H), 7.06–7.10 (m, 4H), 6.95–6.97 (t, 1H, *J* = 5.6 Hz), 6.75(d, 1H, *J* = 8.0 Hz), 6.23(s, 1H), 4.31(d, 2H, *J* = 5.6 Hz), 3.80 (s, 3H), 1.25(s, 9H). ^13^C-NMR (DMSO-*d*_6_) δ: 160.4 (1C, pyrazole), 159.2 (1C, pyrazole), 156.8 (1C, CO), 154.6 (1C, pyrazole), 150.8, 137.6, 136.9, 136.5, 136.3, 133.4, 133.0, 132.9, 129.6, 124.1, 123.2, 119.6, 119.3, 115.0, 111.7, 107.6 (18C, Ar-C), 95.8 (1C, pyrazole), 38.1 (1C, CH_2_), 32.0 (1C, CH_3_-C), 30.2 (3C, C-CH_3_), 20.6 (1C, Ar-CH_3_); MS (ESI): *m*/*z* 517.24 [M + Na]^+^. Mp 201.3–203.5 °C.

*1-(2-((1H-Indazol-5-yl)oxy)benzyl)-3-(3-(tert-butyl)-1-phenyl-1H-pyrazol-5-yl)urea* (**25b**). White solid (3.12 g, 65%). HPLC purity 97.2% (*t*_R_ = 11.13 min). IR (KBr): 3254, 1670, 1596, 1578 cm^−1^; ^1^H-NMR (DMSO-*d*_6_) δ: 13.09 (s, 1H), 8.30 (s, 1H), 7.99 (s, 1H), 7.57 (d, 1H, *J* = 8.8 Hz), 7.48–7.51 (m, 4H), 7.39–7.43 (m, 1H), 7.26–7.29 (m, 3H), 7.10–7.12 (m, 2H), 6.94–6.96 (t, 1H, *J* = 5.6 Hz), 6.75 (d, 2H, *J* = 8.0 Hz), 4.32 (d, 2H, *J* = 5.6 Hz), 1.27 (s, 9H). ^13^C-NMR (DMSO-*d*_6_) δ: 160.6 (1C, pyrazole), 159.4 (1C, pyrazole), 157.1 (1C, CO), 154.8 (1C, pyrazole), 151.0, 153.2, 150.9, 138.0, 136.9, 136.8, 133.8, 133.4, 130.0, 124.5, 121.0, 120.7, 117.4, 115.3, 115.0, 111.1 (18C, Ar-C), 96.2 (1C, pyrazole), 38.5 (1C, CH_2_), 32.6 (1C, CH_3_-C), 30.6 (3C, C-CH_3_); MS (ESI): *m*/*z* 503.14 [M + Na]^+^. Mp 197.1–199.2 °C.

*1-(2-((1H-Indazol-5-yl)oxy)benzyl)-3-(3-(tert-butyl)-1-(m-tolyl)-1H-pyrazol-5-yl)urea* (**25c**). White solid (3.70 g, 75%). HPLC purity 95.5% (*t*_R_ = 11.82 min). IR (KBr): 3255, 1670, 1599, 1574 cm^−1^; NMR (DMSO-*d*_6_) δ: 13.11 (s, 1H), 8.30 (s, 1H), 7.99 (s, 1H), 7.57 (d, 1H, *J* = 8.8 Hz), 7.36–7.39 (m, 1H), 7.25–7.28 (m, 6H), 7.11–7.14 (m, 2H), 6.97–6.99 (t, 1H, *J* = 5.6 Hz), 6.74 (d, 1H, *J* = 8.0 Hz), 6.25 (s, 1H), 4.31 (d, 2H, *J* = 5.6 Hz), 2.46 (s, 3H), 1.24 (s, 9H); ^13^C-NMR (DMSO-*d*_6_) δ: 160.4 (1C, pyrazole), 154.4 (1C, pyrazole), 153.7 (1C, CO), 152.7 (1C, pyrazole), 152.1, 149.5, 146.5, 137.7, 136.5, 136.3, 130.9, 129.6, 129.1, 128.6, 124.2, 124.0, 122.5, 120.0, 119.1, 105.3 (18C, Ar-C), 95.1 (1C, pyrazole), 38.6 (1C, CH_2_), 31.0 (1C, CH_3_-C), 30.2 (3C, C-CH_3_), 22.1 (1C, Ar-CH_3_); MS (ESI): *m*/*z* 517.24 [M + Na]^+^. Mp 200.4–201.9 °C.

*1-(2-((1H-Indazol-5-yl)oxy)benzyl)-3-(3-(tert-butyl)-1-(4-methoxyphenyl)-1H-pyrazol-5-yl)urea* (**25d**). White solid (3.16 g, 62%). HPLC purity 96.3% (*t*_R_ = 12.13 min). IR (KBr): 3259, 1670, 1600, 1579 cm^−1^; ^1^H-NMR (DMSO-*d*_6_) δ: 13.09 (s, 1H), 8.23 (s, 1H), 7.99 (s, 1H), 7.57 (d, 1H, *J* = 8.8 Hz), 7.34–7.37 (m, 3H), 7.25–7.27 (m, 4H), 7.10–7.14 (m, 2H), 6.93–6.95 (t, 1H, *J* = 5.6 Hz), 6.74 (d, 1H, *J* = 8.0 Hz), 6.23 (s, 1H), 4.31 (d, 2H, *J* = 5.6 Hz), 2.35 (s, 3H), 1.25 (s, 9H); ^13^C-NMR (DMSO-*d*_6_) δ: 161.6 (1C, pyrazole), 155.3 (1C, pyrazole), 154.5 (1C, CO), 150.3 (1C, pyrazole), 142.0, 141.3, 138.2, 137.1, 133.4, 130.0, 128.7, 128.4, 126.8, 123.6, 123.2, 123.0, 119.8, 117.4, 111.7, 108.4 (18C, Ar–C), 97.0 (1C, pyrazole), 59.8 (1C, O-CH_3_), 37.5 (1C, CH_2_), 31.0 (1C, CH_3_-C), 30.1 (3C, C-CH_3_); MS (ESI): *m*/*z* 533.23 [M + Na]^+^. Mp 198.0–200.7 °C.

*1-(2-((1H-Indazol-5-yl)oxy)benzyl)-3-(3-(tert-butyl)-1-(4-chlorophenyl)-1H-pyrazol-5-yl)urea* (**25e**). White solid (2.83 g, 55%). HPLC purity 97.7% (*t*_R_ = 12.18 min). IR (KBr): 3257, 1670, 1596, 1577 cm^−1^; ^1^H-NMR (DMSO-*d*_6_) δ: 13.10 (s, 1H), 8.34 (s, 1H), 7.99(s, 1H), 7.57 (d, 1H, *J* = 8.8 Hz), 7.51–7.54 (m, 4H), 7.25–7.28 (m, 3H), 7.10–7.13 (m, 2H), 6.91–6.93 (t, 1H, *J* = 5.6 Hz), 6.75 (d, 1H, *J* = 8.0 Hz), 6.25 (s, 1H), 4.31 (d, 2H, *J* = 5.6 Hz), 1.25 (s, 9H); ^13^C-NMR (DMSO-*d*_6_) δ: 160.6 (1C, pyrazole), 159.4 (1C, pyrazole), 157.0 (1C, CO), 154.8 (1C, pyrazole), 151.1, 137.7, 137.1, 136.6, 136.4, 133.6, 133.1, 133.0, 129.8, 124.3, 123.4, 119.8, 119.4, 115.2, 111.9, 107.7 (18C, Ar–C), 96.1 (1C, pyrazole), 38.3 (1C, CH_2_), 32.2 (1C, CH_3_-C), 30.4 (3C, C-CH_3_); MS (ESI): *m*/*z* 537.19 [M + Na]^+^. Mp 199.6–201.4 °C.

*1-(2-((1H-Indazol-5-yl)oxy)benzyl)-3-(1-(4-bromophenyl)-3-(tert-butyl)-1H-pyrazol-5-yl)urea* (**25f**). White solid (4.01 g, 72%). HPLC purity 97.5% (*t*_R_ = 12.01 min). IR (KBr): 3256, 1670, 1593, 1575 cm^−1^; ^1^H-NMR (DMSO-*d*_6_) δ: 13.13 (s, 1H), 8.38 (s, 1H), 8.00 (s, 1H), 7.66 (d, 2H, *J* = 8.4 Hz), 7.58 (d, 1H, *J* = 8.8), 7.25–7.28 (m, 3H), 7.11–7.13 (m, 2H), 6.94–6.96 (t, 1H, *J* = 5.6 Hz), 6.74 (d, 1H, *J* = 8.0 Hz), 6.27 (s, 1H), 4.31 (d, 2H, *J* = 5.6 Hz), 1.25 (s, 9H); ^13^C-NMR (DMSO-*d*_6_) δ: 161.6 (1C, pyrazole), 155.8 (1C, pyrazole), 155.0 (1C, CO), 150.8 (1C, pyrazole), 138.5, 138.2, 137.5, 133.8, 131.8, 130.5, 129.6, 129.3, 128.8, 126.1, 123.7, 123.4, 120.3, 117.9, 112.1, 108.9 (18C, Ar-C), 96.8 (1C, pyrazole), 38.8 (1C, CH_2_), 32.6 (1C, CH_3_-C), 30.6 (3C, C-CH_3_); MS (ESI): *m*/*z* 581.14 [M + Na]^+^. Mp 203.6–205.7 °C.

*1-(2-((1H-Indazol-5-yl)oxy)benzyl)-3-(3-(tert-butyl)-1-(4-fluorophenyl)-1H-pyrazol-5-yl)urea* (**25g**). White solid (3.23 g, 65%). HPLC purity 96.6% (*t*_R_ = 12.13 min). IR (KBr): 3258, 1670, 1597, 1577 cm^−1^; ^1^H-NMR (DMSO-*d*_6_) δ: 13.12 (s, 1H), 8.31 (s, 1H), 8.00 (s, 1H), 7.58 (d, 1H, *J* = 8.8 Hz), 7.51–7.54 (m, 2H), 7.25–7.28 (m, 5H), 7.10–7.13 (m, 2H), 6.93–6.95 (t, 1H, *J* = 5.6 Hz), 6.74 (d, 1H, *J* = 8.0 Hz), 6.25 (s, 1H), 4.31 (d, 2H, *J* = 5.6 Hz), 1.25 (s, 9H); ^13^C-NMR (DMSO-*d*_6_), δ: 160.7 (1C, pyrazole), 155.2 (1C, pyrazole), 154.4 (1C, CO), 150.2 (1C, pyrazole), 138.0, 135.1, 133.3, 129.9, 128.7, 128.3, 126.5, 126.4, 123.1, 122.9, 119.7, 117.3, 116.0, 115.8, 111.5, 108.0 (18C, Ar-C), 95.5 (1C, pyrazole), 38.2 (1C, CH_2_), 32.0 (1C, CH_3_-C), 30.1 (3C, C-CH_3_); MS (ESI): *m*/*z* 521.11 [M + Na]^+^. Mp 191.1–193.3 °C.

*1-(2-((1H-Indazol-5-yl)oxy)benzyl)-3-(3-(tert-butyl)-1-(4-nitrophenyl)-1H-pyrazol-5-yl)urea* (**25h**). White solid (4.20 g, 80%). HPLC purity 97.3% (*t*_R_ = 11.86 min). IR (KBr): 3256, 1670, 1595, 1578 cm^−1^; ^1^H-NMR (DMSO-*d*_6_) δ: 13.12 (s, 1H), 8.62 (s, 1H), 8.31 (d, 2H, *J* = 8.8 Hz), 8.00 (s, 1H), 7.83 (d, 2H, *J* = 8.8 Hz), 7.57 (d, 1H, *J* = 8.8 Hz), 7.26–7.30 (m, 3H), 7.08–7.11 (m, 3H), 6.74 (d, 1H, *J* = 8.0 Hz), 6.32 (s, 1H), 4.31 (d, 2H, *J* = 5.6 Hz), 1.26 (s, 9H); ^13^C-NMR (DMSO-*d*_6_), δ: 161.6 (1C, pyrazole), 155.8 (1C, pyrazole), 155.0 (1C, CO), 150.8 (1C, pyrazole), 138.6, 138.4, 137.5, 133.9, 132.5, 130.4, 129.3, 128.8, 126.4, 123.7, 123.4, 120.3, 120.1, 117.8, 112.1, 109.9 (18C, Ar-C), 96.9 (1C, pyrazole), 38.8 (1C, CH_2_), 32.6 (1C, CH_3_-C), 30.6 (3C, C-CH_3_); MS (ESI): *m*/*z* 548.21 [M + Na]^+^. Mp 177.3–179.9 °C.

*1-(2-((1H-Indazol-5-yl)oxy)-5-fluorobenzyl)-3-(3-(tert-butyl)-1-(4-nitrophenyl)-1H-pyrazol-5-yl)urea* (**25i**). White solid (3.80 g, 70%). HPLC purity 98.7% (*t*_R_ = 11.91 min). IR (KBr): 3260, 1670, 1599, 1578 cm^−1^; ^1^H-NMR (DMSO-*d*_6_) δ: 13.11 (s, 1H), 8.61 (s, 1H), 8.30 (d, 2H, *J* = 8.8 Hz), 8.01(s, 1H), 7.81 (d, 2H, *J* = 8.8 Hz), 7.51 (d, 1H, *J* = 8.8 Hz), 7.22–7.25 (m, 3H), 7.05–7.08 (m, 2H), 6.72 (d, 1H, *J* = 8.0 Hz), 6.31 (s, 1H), 4.30 (d, 2H, *J* = 5.6 Hz), 1.25 (s, 9H); ^13^C-NMR (DMSO-*d*_6_), δ: 162.7 (1C, pyrazole), 156.9 (1C, pyrazole), 156.1 (1C, CO), 152.0 (1C, pyrazole), 139.6, 139.3, 138.6, 135.0, 132.9, 131.6, 130.7, 130.4, 130.0, 127.2, 124.8, 124.5, 121.4, 119.0, 113.2, 110.0 (18C, Ar-C), 98.0 (1C, pyrazole), 39.9 (1C, CH_2_), 33.7 (1C, CH_3_-C), 31.5 (3C, C-CH_3_); MS (ESI): *m*/*z* 566.20 [M + Na]^+^. Mp 189.1–191.6 °C.

*1-(2-((1H-Indazol-5-yl)oxy)-5-fluorobenzyl)-3-(3-(tert-butyl)-1-(p-tolyl)-1H-pyrazol-5-yl)urea* (**25j**). White solid (3.84 g, 75%). HPLC purity 96.5% (*t*_R_ = 11.84 min). IR (KBr): 3257, 1670, 1596, 1576 cm^−1^; ^1^H-NMR (DMSO-*d*_6_) δ: 13.11 (s, 1H) ,8.20 (s, 1H), 8.00 (s, 1H), 7.53 (d, 1H, *J* = 8.8 Hz), 7.33–7.37 (m, 1H), 7.26–7.30 (m, 3H), 7.04–7.07 (m, 4H), 6.93–6.95 (t, 1H, *J* = 5.6 Hz), 6.71 (d, 1H, *J* = 8.0 Hz), 6.20 (s, 1H), 4.30 (d, 2H, *J* = 5.6 Hz), 3.77(s, 3H), 1.24 (s, 9H). ^13^C-NMR (DMSO-*d*_6_), δ: 160.4 (1C, pyrazole), 159.2 (1C, pyrazole), 156.8 (1C, CO), 154.6 (1C, pyrazole), 150.8, 137.6, 136.9, 136.5, 136.3, 133.4, 133.0, 129.9, 129.5, 124.1, 123.1, 119.6, 119.3, 115.0, 111.7, 107.6, 111.1 (18C, Ar-C), 95.8 (1C, pyrazole), 38.0 (1C, CH_2_), 32.0 (1C, CH_3_-C), 30.2 (3C, C-CH_3_), 20.6 (1c, Ar-CH_3_); MS (ESI): *m*/*z* 535.23 [M + Na]^+^. Mp 188.7–190.3 °C.

*4-(5-(3-(2-((1H-Indazol-5-yl)oxy)benzyl)ureido)-3-(tert-butyl)-1H-pyrazol-1-yl)benzenesulfonamide* (**25k**). White solid (2.96 g, 53%). HPLC purity 97.3% (*t*_R_ = 9.57 min). IR (KBr): 3257, 1670, 1595, 1575 cm^−1^; ^1^H-NMR (DMSO-*d*_6_) δ: 13.10 (s, 1H), 8.49 (s, 1H), 8.00 (s, 1H), 7.92 (d, 1H, *J* = 8.8 Hz), 7.71 (d, 2H, *J* = 8.8 Hz), 7.59 (d, 1H, *J* = 8.8 Hz), 7.47 (s, 2H), 7.29–7.31 (m, 3H), 7.13–7.16 (m, 2H), 6.99–7.02 (m, 1H), 6.77 (d, 1H, *J* = 6.4 Hz), 6.36 (s, 1H), 4.70 (s, 1H), 4.34 (d, 2H, *J* = 5.6 Hz), 1.27 (s, 9H); ^13^C-NMR (DMSO-*d*_6_) δ: 163.5 (1C, pyrazole), 157.7 (1C, pyrazole), 156.9 (1C, CO), 152.7 (1C, pyrazole), 140.5, 140.3, 139.4, 135.8, 134.4, 132.3, 131.2, 130.7, 128.3, 125.6, 125.3, 122.2, 122.0, 119.7, 114.0, 110.8 (18C, Ar-C), 96.3 (1C, pyrazole), 38.6 (1C, CH_2_), 34.8 (1C, CH_3_-C), 32.4 (1C, C-CH_3_); MS (ESI): *m*/*z* 582.20 [M + Na]^+^. Mp 194.7–196.8 °C.

#### 3.2.12. General Method for the Synthesis of **26a**–**26f**

A solution of the corresponding **3a**–**3i** (1.1 equiv.) and **14** (1 equiv.) was stirred 2 h at 90 °C in the presence of Et_3_N (1 mL) in DMSO (50 mL). The mixture was poured into ice water, and the solution was extracted with EtOAc. The organic layer was washed with water and brine, dried (MgSO_4_), filtered, and evaporated to dryness. The product was separated by column chromatography using DCM/MeOH (150:1) as eluent.

*1-(5-((1H-Indazol-5-yl)oxy)-2H-chromen-8-yl)-3-(3-(tert-butyl)-1-(p-tolyl)-1H-pyrazol-5-yl)urea* (**26a**). From **3a** (4.03 g, 10 mmol), and **14** (2.79 g, 10 mmol), after work-up and purification the title compound (3.73 g, 70%) was obtained as a white solid. HPLC purity 95.4% (*t*_R_ = 10.32 min). IR (KBr): 3256, 1670, 1640, 1595, 1578 cm^−1^; ^1^H-NMR (DMSO-*d*_6_) δ: 13.07 (s, 1H), 8.92 (s, 1H), 8.51 (s, 1H), 7.97 (s, 1H), 7.87 (d, 1H, *J* = 8.8 Hz), 7.54 (d, 2H, *J* = 8.8 Hz), 7.37–7.40 (m, 4H), 7.17 (d, 1H, *J* = 2.4 Hz), 7.11 (dd, 1H, *J* = 2.4, 8.8 Hz), 6.61–6.64 (m, 1H), 6.38(s, 1H), 6.34(d, 1H, *J* = 8.8 Hz), 5.93–5.95 (m, 1H), 4.85 (d, 2H, *J* = 1.6 Hz), 2.36 (s, 3H), 1.24 (s, 9H); ^13^C-NMR (DMSO-*d*_6_) δ: 160.4 (1C, pyrazole), 159.8 (1C, pyrazole), 157.5 (1C, CO), 154.6 (1C, pyrazole), 153.1, 149.5, 149.2, 146.5, 137.5, 136.5, 136.3, 134.7, 129.5, 124.0, 123.9, 122.5, 121.3, 120.1, 115.7, 115.0, 114.7, 104.5, 95.8 (1C, pyrazole), 73.5 (1C, O-CH_2_), 32.0 (1C, CH_3_-C), 30.2 (3C, C-CH_3_), 20.6 (1C, Ar-CH_3_); MS (ESI): *m*/*z* 557.20 [M + Na]^+^. Mp 210.3–212.7 °C.

*1-(5-((1H-Indazol-5-yl)oxy)-2H-chromen-8-yl)-3-(3-(tert-butyl)-1-phenyl-1H-pyrazol-5-yl)urea* (**26b**). White solid (2.86 g, 55%). HPLC purity 95.9% (*t*_R_ = 10.43 min). IR (KBr): 3259, 1670, 1644, 1599, 1579 cm^−1^; ^1^H-NMR (DMSO-*d*_6_) δ: 13.07 (s, 1H), 8.99 (s, 1H), 8.51 (s, 1H), 7.97 (s, 1H), 7.86 (d, 1H, *J* = 8.8 Hz), 7.53 (m, 5H), 7.41 (m, 1H), 7.16 (d, 1H, *J* = 2.4 Hz), 7.12 (dd, 1H, *J* = 2.4, 8.8 Hz), 6.61–6.64 (m, 1H), 6.39 (s, 1H), 6.36 (d, 1H, *J* = 8.8 Hz), 5.93–5.96 (m, 1H), 4.85 (d, 2H, *J* = 1.6 Hz), 1.24 (s, 9H); ^13^C-NMR (DMSO-*d*_6_) δ: 162.5 (1C, pyrazole), 161.2 (1C, pyrazole), 160.3 (1C, CO), 160.1 (1C, pyrazole), 157.9, 155.1, 153.5, 149.7, 149.3, 147.0, 138.2, 135.7, 134.4, 134.3, 126.8, 126.7, 123.1, 121.8, 116.5, 116.2, 115.4, 105.0, 96.6 (1C, pyrazole), 74.3 (1C, O-CH_2_), 32.4 (1C, CH_3_-C), 30.6 (3C, C-CH_3_); MS (ESI): *m*/*z* 543.22 [M + Na]^+^. Mp 209.9–212.1 °C.

*4-(5-(3-(5-((1H-Indazol-5-yl)oxy)-2H-chromen-8-yl)ureido)-3-(tert-butyl)-1H-pyrazol-1-yl)benzenesulfonamide* (**26c**). White solid (2.99 g, 50%). HPLC purity 96.6% (*t*_R_ = 8.33 min). IR (KBr): 3253, 1670, 1641, 1595, 1575 cm^−1^; _1_H-NMR (DMSO-*d*_6_) δ: 13.08 (s, 1H), 9.14 (s, 1H), 8.52 (s, 1H), 7.99–8.01 (m, 3H), 7.86–7.89 (d, 1H, *J* = 8.8 Hz), 7.73–7.75 (d, 2H, *J* = 8.8 Hz), 7.54–7.57 (m, 3H), 7.17 (s, 1H), 7.10 (d, 1H, *J* = 8.8 Hz), 6.62 (d, 1H, *J* = 10.0 Hz), 6.41–6.44 (m, 2H), 5.93–5.95 (m, 1H), 4.87 (d, 2H, *J* = 1.6 Hz), 1.24 (s, 9H); ^13^C-NMR (DMSO-*d*_6_) δ: 162.5 (1C, pyrazole), 161.2 (1C, pyrazole), 160.3 (1C, CO), 160.1 (1C, pyrazole), 158.0, 155.1, 153.6, 150.4, 149.6, 147.1, 138.2, 135.7, 134.5, 134.4, 126.8, 126.7, 124.6, 123.2, 121.9, 116.5, 116.2, 104.9, 96.7 (1C, pyrazole), 74.9 (1C, O-CH_2_), 32.5 (1C, CH_3_-C), 30.6 (3C, C-CH_3_); MS (ESI): *m*/*z* 622.20 [M + Na]^+^. Mp 213.4–215.2 °C.

*1-(5-((1H-Indazol-5-yl)oxy)-2H-chromen-8-yl)-3-(3-(tert-butyl)-1-(4-methoxyphenyl)-1H-pyrazol-5-yl)urea* (**26d**). White solid (2.47 g, 45%). HPLC purity 96.2% (*t*_R_ = 10.04 min). IR (KBr): 3256, 1670, 1640, 1593, 1574 cm^−1^; ^1^H-NMR (DMSO-*d*_6_) δ: 13.06 (s, 1H), 8.22 (s, 1H), 7.96 (s, 1H), 7.86 (d, 2H, *J* = 8.8 Hz), 7.71 (s, 1H), 7.53 (d, 1H, *J* = 8.8 Hz), 7.33 (d, 2H, *J* = 8.0 Hz), 7.25 (d, 3H, *J* = 8.0 Hz), 6.58–6.61 (m, 1H), 6.38 (d, 1H, *J* = 8.8 Hz), 5.88–5.93 (m, 1H), 4.84 (d, 2H, *J* = 1.6 Hz), 2.41 (s, 3H), 1.24 (s, 9H). ^13^C-NMR (DMSO-*d*_6_) δ: 163.2 (1C, pyrazole), 161.9 (1C, pyrazole), 161.1 (1C, CO), 160.8 (1C, pyrazole), 158.7, 155.8, 154.2, 150.4, 150.0, 147.6, 138.9, 136.4, 135.1, 135.0, 127.5, 127.4, 123.8, 122.5, 117.2, 117.0, 116.1, 105.7, 97.4 (1C, pyrazole), 71.2 (1C, O-CH_2_), 51.2 (1C, O-CH_3_), 33.1 (1C, CH_3_-C), 31.3 (3C, C-CH_3_); MS (ESI): *m*/*z* 573.23 [M + Na]^+^. Mp 215.1–217.1 °C.

*1-(5-((1H-Indazol-5-yl)oxy)-2H-chromen-8-yl)-3-(3-(tert-butyl)-1-(4-fluorophenyl)-1H-pyrazol-5-yl)urea* (**26e**). White solid (2.69 g, 45%). HPLC purity 98.3% (*t*_R_ = 10.65 min). IR (KBr): 3253, 1670, 1637, 1599, 1579 cm^−1^; ^1^H-NMR (DMSO-*d*_6_) δ: 13.07 (s, 1H), 8.95 (s, 1H), 8.46 (s, 1H), 7.97 (s, 1H), 7.86 (d, 1H, *J* = 8.8 Hz), 7.53–7.56 (m, 3H), 7.38–7.41 (m, 2H), 7.17 (d, 1H, *J* = 2.4 Hz), 7.11 (dd, 1H, *J* = 2.4, 8.8 Hz), 6.61–6.65 (m, 1H), 6.39 (s, 1H), 6.35 (d, 1H, *J* = 8.8 Hz), 5.92–5.95 (m, 1H), 4.85 (d, 2H, *J* = 1.6 Hz), 1.24 (s, 9H); ^13^C-NMR (DMSO-*d*_6_) δ: 162.8 (1C, pyrazole), 161.5 (1C, pyrazole), 160.6 (1C, CO), 160.4 (1C, pyrazole), 158.2, 155.4, 153.8, 150.0, 149.6, 147.2, 138.5, 136.0, 134.7, 134.6, 127.1, 127.0, 123.4, 122.1, 116.7, 116.5, 115.7, 105.3, 96.9 (1C, pyrazole), 74.6 (1C, O-CH_2_), 32.8 (3C, CH_3_-C), 32.3 (1C, C-CH_3_); MS (ESI): *m*/*z* 561.21 [M + Na]^+^. Mp 197.3–199.7 °C.

*1-(5-((1H-Indazol-5-yl)oxy)-2H-chromen-8-yl)-3-(3-(tert-butyl)-1-(4-nitrophenyl)-1H-pyrazol-5-yl)urea* (**26f**). Yellow solid (3.50 g, 62%). HPLC purity 97.6% (*t*_R_ = 10.54 min). IR (KBr): 3254, 1670, 1633, 1601, 1581 cm^−1^; ^1^H-NMR (DMSO-*d*_6_) δ: 13.03 (s, 1H), 9.15 (s, 1H), 8.43 (s, 1H), 8.35 (dd, 1H, *J* = 2.0, 8.8 Hz), 7.93 (s, 1H), 7.81–7.84 (m, 3H), 7.50–7.53 (d, 1H, *J* = 8.8 Hz), 7.12 (d, 1H, *J* = 2.0 Hz), 7.06 (d, 1H, *J* = 2.0, 8.8 Hz), 6.58 (m, 1H), 6.40 (s, 1H), 6.34 (d, 1H, *J* = 8.8 Hz), 5.90–5.95 (m, 1H), 4.84 (d, 2H, *J* = 1.6 Hz), 1.24 (s, 9H); ^13^C-NMR (DMSO-*d*_6_) δ: 162.0 (1C, pyrazole), 154.5 (1C, pyrazole), 153.0 (1C, CO), 149.2 (1C, pyrazole), 148.0, 146.2, 137.7, 136.9, 135.1, 133.8, 126.2, 126.1, 123.8, 122.1, 121.7, 121.1, 120.0, 115.9, 115.7, 115.4, 115.1, 104.5, 96.1 (1C, pyrazole), 74.4 (1C, O-CH_2_), 31.3 (1C, CH_3_-C), 30.1 (3C, C-CH_3_); 588.21 [M + Na]^+^. Mp 207.1–209.6 °C.

#### 3.2.13. General Method for the Synthesis of **27a**–**27e**

A solution of the corresponding **3a**–**3i** (1.1 equiv.) and **18a**–**18b** (1 equiv.) was stirred 2 h at 90 °C in the presence of Et_3_N (1 mL) in DMSO (50 mL). Then the mixture was poured into ice water and the solution was extracted with EtOAc. The organic layer was washed with water and brine, dried (MgSO_4_), filtered and evaporated to dryness. The product was separated by column chromatography using DCM/MeOH (80:1) as eluent.

*1-(3-(tert-Butyl)-1-(p-tolyl)-1H-pyrazol-5-yl)-3-(2-((5-hydroxy-5,6,7,8-tetrahydronaphthalen-2-yl)oxy)-benzyl)urea* (**27a**). From 3a (4.03 g, 10 mmol), and **18a** (2.69 g, 10 mmol), after work-up and purification, 27a (3.66 g, 70%) was obtained as a white solid. HPLC purity 96.6% (*t*_R_ = 8.46 min). IR (KBr): 3252, 1670, 1595, 1575 cm^−1^; ^1^H-NMR (DMSO-*d*_6_) δ: 8.25 (s, 1H), 7.38–7.41 (m, 2H), 7.35–7.38 (m, 2H), 7.11–7.15 (m, 3H), 7.15–7.18 (m, 1H), 6.91–6.93 (t, 1H, *J* = 8.0 Hz), 6.86–6.89 (m, 1H), 6.74–6.77 (m, 1H), 6.61 (d, 1H, *J* = 8.8 Hz), 6.24 (s, 1H), 5.09–5.12 (m, 1H), 4.54 (s, 1H), 4.28 (d, 2H, *J* = 5.6 Hz), 2.65–2.68 (m, 2H), 2.37 (s, 3H), 1.87–1.91 (m, 2H), 1.63–1.67 (m, 2H), 1.29 (s, 9H). ^13^C-NMR (DMSO-*d*_6_) δ: 160.9 (1C, pyrazole), 156.0 (1C, CO), 154.9 (1C, pyrazole), 154.5, 139.0, 138.3, 137.0, 136.7, 135.9, 131.3, 130.7, 130.1, 129.3, 128.9, 124.7, 124.1, 119.2, 117.6, 116.0 (18C, Ar-C), 95.8 (1C, pyrazole), 66.4 (1C, HO-C), 38.6 (1C, N-CH_2_), 32.9 (1C, CH_2_), 32.5 (1C, C), 31.5, (1C, CH_2_), 30.7 (3C, CH_3_), 21.3 (1C, Ar–CH_3_), 19.4 (1C, CH_2_). MS (ESI): *m*/*z* 547.20 [M + Na]^+^. Mp 201.6–203.7 °C.

*1-(3-(tert-Butyl)-1-(4-nitrophenyl)-1H-pyrazol-5-yl)-3-(2-((5-hydroxy-5,6,7,8-tetrahydronaphthalen-2-yl)-oxy)benzyl)urea* (**27b**). White solid (2.78 g, 50%). HPLC purity 96.7% (*t*_R_ = 8.39 min). IR (KBr): 3251, 1667, 1593, 1573 cm^−1^; ^1^H-NMR (DMSO-*d*_6_) δ: 8.59 (s, 1H), 8.33 (s, 2H), 7.83 (d, 2H, *J* = 8.8 Hz), 7.38 (d, 1H, *J* = 8.8 Hz), 7.25–7.28 (m, 2H), 7.09–7.11 (m, 1H), 6.96–6.98 (t, 1H, *J* = 8.0 Hz), 6.81–6.85 (m, 1H), 6.74–6.77 (m, 1H), 6.61 (d, 1H, *J* = 8.8 Hz), 6.33 (s, 1H), 5.08–5.12 (m, 1H), 4.53 (s, 1H), 4.24 (d, 2H, *J* = 5.6 Hz), 2.64–2.67 (m, 2H), 1.87–1.90 (m, 2H), 1.63–1.66 (m, 2H), 1.27 (s, 9H); ^13^C-NMR (DMSO-*d*_6_) δ: 162.9 (1C, pyrazole), 155.9 (1C, CO), 155.1 (1C, pyrazole), 154.4, 145.3, 144.8, 139.1, 139.0, 135.9, 131.2, 130.7, 129.2, 128.9, 125.3, 124.0, 123.6, 119.1, 117.6, 116.0 (18C, Ar-C), 99.1 (1C, pyrazole), 66.4 (1C, HO-C), 38.7 (1C, N-CH_2_), 32.8 (1C, CH_2_), 32.7 (1C, C), 30.5 (3C, CH_3_), 29.5 (1C, CH_2_), 19.4 (1C, CH_2_); MS (ESI): *m*/*z* 578.05 [M + Na]^+^. Mp 202.1–204.3 °C.

*1-(3-(tert-Butyl)-1-phenyl-1H-pyrazol-5-yl)-3-(2-((5-hydroxy-5,6,7,8-tetrahydronaphthalen-2-yl)oxy)-benzyl)urea* (**27c**). White solid (2.14 g, 42%). HPLC purity 98.8% (*t*_R_ = 8.41 min). IR (KBr): 3250, 1667, 1593, 1573 cm^−1^; ^1^H-NMR (DMSO-*d*_6_) δ: 8.31 (s, 1H), 7.49–7.52 (m, 4H), 7.39–7.42 (m, 2H), 7.26–7.28 (m, 2H), 7.11–7.14 (m, 1H), 6.92–6.94 (t, 1H, *J* = 8.0 Hz), 6.83–6.86 (m, 1H), 6.72–6.75 (m, 1H), 6.61 (d, 1H), 6.26 (s, 1H), 5.08–6.12 (m, 1H), 4.53 (s, 1H), 4.24 (d, 2H, *J* = 5.6 Hz), 2.64–2.68 (m, 2H), 1.87–1.91 (m, 2H), 1.67–1.71 (m, 2H), 1.25–1.29 (s, 9H); ^13^C-NMR (DMSO-*d*_6_) δ: 160.7 (1C, pyrazole), 155.5 (1C, CO), 154.5 (1C, pyrazole), 153.9, 138.8, 138.5, 137.8, 135.4, 130.8, 130.2, 129.2, 128.8, 128.4, 127.1, 124.2, 123.7, 118.7, 117.1, 115.5 (18C, Ar-C), 95.7 (1C, pyrazole), 65.9 (1C, HO-C), 38.1 (1C, N-CH_2_), 32.4 (1C, CH_2_), 32.0 (1C, C), 30.2 (3C, CH_3_), 29.0 (1C, CH_2_), 18.9 (1C, CH_2_); MS (ESI): *m*/*z* 533.07 [M + Na]^+^. Mp 202.5–204.8 °C.

*1-(3-(tert-Butyl)-1-(4-nitrophenyl)-1H-pyrazol-5-yl)-3-(5-fluoro-2-((5-hydroxy-5,6,7,8-tetrahydro-naphthalen-2-yl)oxy)benzyl)urea* (**27d**). White solid (3.72 g, 65%). HPLC purity 97.4% (t_R_ = 8.51 min). IR (KBr): 3251, 1668, 1593, 1573 cm^−1^; ^1^H-NMR (DMSO-*d*_6_) δ: 8.59 (s, 1H), 8.33–8.36 (m, 2H), 7.83–7.88 (m, 2H), 7.38–7.41 (m, 1H), 7.25–7.29 (m, 1H), 7.09–7.13 (m, 1H), 6.96–6.98 (t, 1H, *J* = 8.0 Hz), 6.81–6.85 (m, 1H), 6.72–6.76 (m, 1H), 6.61 (d, 1H, *J* = 8.8 Hz), 6.31 (s, 1H), 5.05–5.07 (m, 1H), 4.53 (s, 1H), 4.23 (d, 2H, *J* = 5.6 Hz), 2.64–2.68 (m, 2H), 1.86–1.89 (m, 2H), 1.63–1.66 (m, 2H), 1.25 (s, 9H). ^13^C-NMR (DMSO-*d*_6_) δ: 162.8 (1C, pyrazole), 160.0 (1C, CO), 157.7 (1C, pyrazole), 156.2, 155.3, 150.0, 145.3, 144.8, 139.0, 138.8, 135.8, 134.2, 130.7, 125.2, 123.5, 121.4, 116.9, 115.3, 115.0 (18C, Ar-C), 99.8 (1C, pyrazole), 66.4 (1C, HO-C), 38.7 (1C, N-CH_2_), 32.8 (1C, CH_2_), 32.7 (1C, C), 30.4 (3C, CH_3_), 29.5 (1C, CH_2_), 19.4 (1C, CH_2_); MS (ESI): *m*/*z* 596.04 [M + Na]^+^. Mp 191.4–193.9 °C.

*1-(3-(tert-Butyl)-1-(p-tolyl)-1H-pyrazol-5-yl)-3-(5-fluoro-2-((5-hydroxy-5,6,7,8-tetrahydronaphthalen-2-yl)-oxy)benzyl)urea* (**27e**). White solid (2.81 g, 52%). HPLC purity 97.3% (t_R_ = 8.43 min). IR (KBr): 3251, 1667, 1597, 1576 cm^−1^; ^1^H-NMR (DMSO-*d*_6_) δ: 8.29 (s, 1H), 7.37–7.40 (m, 2H), 7.35–7.39 (m, 2H), 7.11–7.14 (m, 2H), 6.95–6.97 (t, 1H, *J* = 8.0 Hz), 6.86–6.91 (m, 1H), 6.98–7.01 (m, 1H), 6.70–6.77 (m, 1H), 6.58 (d, 1H, *J* = 8.8 Hz), 6.23 (s, 1H), 5.07–5.09 (m, 2H), 4.52 (s, 1H), 4.18 (d, 2H, *J* = 5.6 Hz), 2.61–2.65 (m, 2H), 2.35 (s, 3H), 1.84–1.87 (m, 2H), 1.63–1.66 (m, 2H), 1.21 (s, 9H). ^13^C-NMR (DMSO-*d*_6_) δ: 160.9 (1C, pyrazole), 160.1 (1C, CO), 157.7 (1C, pyrazole), 156.3, 155.0, 150.0, 139.0, 138.0, 136.8, 135.8, 130.7, 130.1, 124.6, 121.5, 121.4, 117.0, 115.5, 115.4, 115.3 (18C, Ar-C), 96.2 (1C, pyrazole), 66.4 (1C, HO-C), 38.4 (1C, N-CH_2_), 32.9 (1C, CH_2_), 32.5 (1C, C), 30.7 (3C, CH_3_), 29.5 (1C, CH_2_), 21.1 (1C, Ar-CH_3_), 19.4 (1C, CH_2_).MS (ESI): *m*/*z* 565.09 [M + Na]^+^. Mp 200.1–203.8 °C.

#### 3.2.14. General Method for the Synthesis of **28a**–**28i**

A solution of the corresponding **3a**–**3i** (1.1 equiv.) and **21** (1 equiv.) was stirred 2 h at 90 °C in the presence of Et_3_N (1 mL) in DMSO (50 mL). Then the mixture was poured into ice water and the solution was extracted with EtOAc. The organic layer was washed with water and brine, dried (MgSO_4_), filtered and evaporated to dryness. The product was separated by column chromatography using DCM/MeOH (100:1) as eluent.

*1-(3-(tert-Butyl)-1-(p-tolyl)-1H-pyrazol-5-yl)-3-(2-(2-morpholinoethoxy)phenyl)urea* (**28a**). From 3a (4.03 g, 10 mmol), and **21** (2.22 g, 10 mmol), **28a** (3.43 g, 72%) was obtained as a white solid. HPLC purity 95.9% (*t*_R_ = 8.02 min). IR (KBr): 3247, 1666, 1593, 1573cm^−1^; ^1^H-NMR (DMSO-*d*_6_) δ: 9.04 (s, 1H), 8.29 (d, 1H, *J* = 8.0 Hz), 7.38–7.41 (m, 4H), 7.02 (t, 1H), 6.93–6.96 (m, 2H), 6.34 (s, 1H), 4.13–4.15 (t, 2H, *J* = 5.6 Hz), 3.54–3.57 (m, 4H), 2.70–2.76 (m, 2H), 2.44–2.48 (m, 4H), 2.36 (s, 3H), 1.25 (s, 9H); MS (ESI): *m*/*z* 500.27 [M + Na]^+^. Mp 166.3–168.2 °C.

*1-(3-(tert-Butyl)-1-phenyl-1H-pyrazol-5-yl)-3-(2-(2-morpholinoethoxy)phenyl)urea* (**28b**). White solid (2.31 g, 50%). HPLC purity 98.9% (*t*_R_ = 8.11 min). IR (KBr) 3244, 1663, 1598, 1577 cm^−1^; ^1^H-NMR (DMSO-*d*_6_) δ: 9.06 (s, 1H), 8.23 (d, 1H, *J* = 8.0 Hz), 7.35–7.39 (m, 4H), 7.00–7.04 (m, 2H), 6.91–6.95 (m, 2H), 6.30 (s, 1H), 4.13–4.15 (t, 2H, *J* = 5.6 Hz), 3.55–3.58 (m, 4H), 2.73–2.76 (m, 2H), 2.45–2.48 (m, 4H), 1.26 (s, 9H); MS (ESI): *m*/*z* 486.26 [M + Na]^+^. Mp 179.2–182.3 °C.

*1-(3-(tert-Butyl)-1-(m-tolyl)-1H-pyrazol-5-yl)-3-(2-(2-morpholinoethoxy)phenyl)urea* (**28c**). White solid (3.00 g, 63%). HPLC purity 97.7% (*t*_R_ = 8.05 min). IR (KBr): 3245, 1664, 1591, 1571 cm^−1^; ^1^H-NMR (DMSO-*d*_6_) δ: 9.05 (s, 1H), 8.23 (d, 1H, *J* = 8.0 Hz), 7.36–7.40 (m, 4H), 7.03–7.07 (m, 1H), 6.91–6.94 (m, 2H), 6.36 (s, 1H), 4.13–4.15 (t, 2H, *J* = 5.6 Hz), 3.55–3.59 (m, 4H), 2.72–2.76 (m, 2H), 2.42–2.46 (m, 4H), 2.33 (s, 3H), 1.26 (s, 9H); MS (ESI): *m*/*z* 500.27 [M + Na]^+^. Mp 191.6–193.7 °C.

*1-(3-(tert-Butyl)-1-(4-methoxyphenyl)-1H-pyrazol-5-yl)-3-(2-(2-morpholinoethoxy)phenyl)urea* (**28d**). White solid (2.71 g, 55%). HPLC purity 96.6% (*t*_R_ = 8.13 min). IR (KBr): 3246, 1659, 1594, 1575 cm^−1^; ^1^H-NMR (DMSO-*d*_6_) δ: 9.06 (s, 1H), 8.25 (d, 1H, *J* = 8.0 Hz), 7.34–7.37 (m, 4H), 7.05–7.08 (m, 1H), 6.93–6.97 (m, 2H), 6.35 (s, 1H), 4.13–4.15 (t, 2H, *J* = 5.6 Hz), 3.84 (3H, O–CH_3_), 3.57–3.61 (m, 4H), 2.72–2.76 (m, 2H), 2.42–2.48 (m, 4H), 1.25 (s, 9H); MS (ESI): *m*/*z* 516.24 [M + Na]^+^. Mp 154.2–156.4 °C.

*1-(3-(tert-Butyl)-1-(4-chlorophenyl)-1H-pyrazol-5-yl)-3-(2-(2-morpholinoethoxy)phenyl)urea* (**28e**). White solid (2.23 g, 45%). HPLC purity 99.1% (*t*_R_ = 8.15 min). IR (KBr): 3243, 1657, 1597, 1577 cm^−1^; ^1^H-NMR (DMSO-*d*_6_) δ: 9.12 (s, 1H), 8.23 (d, 1H, *J* = 8.0), 7.33–7.36 (m, 4H), 7.07–7.11 (m, 1H), 6.94–6.99 (m, 2H), 6.37 (s, 1H), 4.13–4.15 (t, 2H, *J* = 5.6), 3.59–3.62 (m, 4H), 2.74–2.78 (m, 2H), 2.44–2.48 (m, 4H), 1.26 (s, 9H); MS (ESI): *m*/*z* 520.22 [M + Na]^+^. Mp 171.3–173.2 °C.

*1-(1-(4-Bromophenyl)-3-(tert-butyl)-1H-pyrazol-5-yl)-3-(2-(2-morpholinoethoxy)phenyl)urea* (**28f**). White solid (3.78 g, 70%). HPLC purity 96.4% (*t*_R_ = 8.12 min). IR (KBr): 3243, 1657, 1597, 1577 cm^−1^; ^1^H-NMR (DMSO-*d*_6_) δ: 9.13 (s, 1H), 8.20 (d, 1H, *J* = 8.0 Hz), 7.30–7.33 (m, 4H), 7.05–7.09 (m, 1H), 6.95–7.00 (m, 2H), 6.35 (s, 1H), 4.13–4.15 (t, 2H, *J* = 5.6 Hz), 3.57–4.02 (m, 4H), 2.76–2.81 (m, 2H), 2.42–2.45 (m, 4H), 1.24 (s, 9H); MS (ESI): *m*/*z* 564.17 [M + Na]^+^. Mp 189.6–191.3 °C.

*1-(3-(tert-Butyl)-1-(4-fluorophenyl)-1H-pyrazol-5-yl)-3-(2-(2-morpholinoethoxy)phenyl)urea* (**28g**). White solid (2.84 g, yield 69%). HPLC purity 97.2% (*t*_R_ = 8.13 min). IR (KBr): 3246, 1655, 1594, 1575 cm^−1^; ^1^H-NMR (DMSO-*d*_6_), δ: 9.13 (s, 1H), 8.23 (d, 1H, *J* = 8.0 Hz), 7.35–7.39 (m, 4H), 7.08–7.12 (m, 1H), 6.97–7.02 (m, 2H), 6.38 (s, 1H),4.16–4.18 (t, 2H, *J* = 5.6 Hz), 3.60–3.65 (m, 4H), 2.77–2.81 (m, 2H), 2.44–2.49 (m, 4H), 1.25 (s, 9H); MS (ESI): *m*/*z* 504.17 [M + Na]^+^. Mp 170.3–172.0 °C.

*1-(3-(tert-Butyl)-1-(4-nitrophenyl)-1H-pyrazol-5-yl)-3-(2-(2-morpholinoethoxy)phenyl)urea* (**28h**). White solid (2.54 g, 50%). HPLC purity 98.8% (*t*_R_ =8.10 min). IR (KBr): 3244, 1656, 1600, 1578 cm^−1^; ^1^H-NMR (DMSO-*d*_6_), δ: 9.10 (s, 1H), 8.21 (d, 1H, *J* = 8.0 Hz), 7.36–7.42 (m, 4H), 7.09–7.12 (m, 1H), 6.98–7.02 (m, 2H), 6.39 (s, 1H), 4.17–4.19 (t, 2H, *J* = 5.6 Hz), 3.61–3.66 (m, 4H), 2.78–2.82 (m, 2H), 2.46–2.51 (m, 4H), 1.26 (s, 9H); MS (ESI): *m*/*z* 531.24 [M + Na]^+^. Mp 163.3–165.6 °C.

*4-(3-(tert-Butyl)-5-(3-(2-(2-morpholinoethoxy)phenyl)ureido)-1H-pyrazol-1-yl)benzenesulfonamide* (**28i**). White solid (2.81 g, 52%). HPLC purity 98.4% (*t*_R_ = 7.53 min). IR (KBr): 3246, 1657, 1601, 1579 cm^−1^; ^1^H-NMR (DMSO-*d*_6_) δ: 9.09 (s, 1H), 8.23 (d, 1H, *J* = 8.0 Hz), 7.35–7.41 (m, 4H), 7.08–7.12 (m, 1H), 6.97–7.02 (m, 2H), 6.37 (s, 1H), 4.19–4.21 (t, 2H, *J* = 5.6 Hz), 3.63–3.67 (m, 4H), 2.75–2.77 (m, 2H), 2.43–2.48 (m, 4H), 1.25 (s, 9H); MS (ESI): *m*/*z* 565.23 [M + Na]^+^. Mp 172.2–174.1 °C.

#### 3.2.15. General Method for the Synthesis of **29a**–**29i**

A solution of the corresponding **3a**–**3i** (1.1 equiv.) and **24** (1 equiv.) was stirred 2 h at 90 °C in the presence of Et_3_N (1 mL) in DMSO (50 mL). Then the mixture was poured into ice water and the solution was extracted with EtOAc. The organic layer was washed with water and brine, dried (MgSO_4_), filtered and evaporated to dryness. The product was separated by column chromatography using DCM/MeOH (100:1) as eluent.

*1-(2-((1H-Indazol-5-yl)oxy)phenyl)-3-(3-(tert-butyl)-1-(p-tolyl)-1H-pyrazol-5-yl)urea* (**29a**). From **3a** (4.03 g, 10 mmol), and **24** (2.25 g, 10 mmol), after work-up and purification **29a** (2.88 g, 60%) was obtained as a white solid. HPLC purity 96.6% (*t*_R_ = 11.32 min). IR (KBr): 3247, 1656, 1600, 1578 cm^−1^; ^1^H-NMR (DMSO-*d*_6_) δ: 13.16 (s, 1H), 9.07 (s, 1H), 8.87 (s, 1H), 8.20 (d, 1H, *J* = 8.8 Hz), 8.04 (s, 1H), 7.53–7.59 (m, 3H), 7.40–7.44 (m, 3H), 7.37–7.39 (m, 1H), 7.35–7.37 (m, 1H), 7.30–7.34 (m, 1H), 7.13–7.16 (m, 1H), 6.39 (s, 1H), 2.36 (s, 3H), 1.25 (s, 9H); MS (ESI): *m*/*z* 503.13 [M + Na]^+^. Mp 201.1–203.4 °C.

*1-(2-((1H-Indazol-5-yl)oxy)phenyl)-3-(3-(tert-butyl)-1-phenyl-1H-pyrazol-5-yl)urea* (**29b**). White solid (1.95 g, 42%). HPLC purity 98.1% (*t*_R_ = 11.41 min). IR (KBr): 3245, 1657, 1601, 1579 cm^−1^; ^1^H-NMR (DMSO-*d*_6_) δ: 13.07 (s, 1H), 8.32 (s, 1H), 7.97 (s, 1H), 7.59 (d, 1H, *J* = 8.8 Hz), 7.49–7.55 (m, 4H), 7.37–7.42 (m, 1H), 7.27–7.32 (m, 3H), 7.12–7.15 (m, 2H), 6.95–6.97 (t, 1H, *J* = 5.6 Hz), 6.76 (d, 2H, *J* = 8.0 Hz), 1.25 (s, 9H). MS (ESI): *m*/*z* 489.13 [M + Na]^+^. Mp 188.2–190.3 °C.

*1-(2-((1H-Indazol-5-yl)oxy)phenyl)-3-(3-(tert-butyl)-1-(m-tolyl)-1H-pyrazol-5-yl)urea* (**29c**). White solid (2.64 g, 55%). HPLC purity 98.8% (*t*_R_ =11.37 min). IR (KBr): 3246, 1652, 1594, 1574 cm^−1^; ^1^H-NMR (DMSO-*d*_6_) δ: 13.08 (s, 1H), 8.31 (s, 1H), 7.97 (s, 1H), 7.54 (d, 1H, *J* = 8.8 Hz), 7.37–7.42 (m, 1H), 7.24–7.29 (m, 6H), 7.13–7.16 (m, 2H), 6.95–6.97 (t, 1H, *J* = 5.6 Hz), 6.73 (d, 1H, *J* = 8.0 Hz), 6.23 (s, 1H), 2.45 (s, 3H), 1.26 (s, 9H); MS (ESI): *m*/*z* 503.56 [M + Na]^+^. Mp 194.3–196.5 °C.

*1-(2-((1H-Indazol-5-yl)oxy)phenyl)-3-(3-(tert-butyl)-1-(4-methoxyphenyl)-1H-pyrazol-5-yl)urea* (**29d**). White solid (2.13 g, 43%). HPLC purity 97.3% (*t*_R_ = 11.11 min). IR (KBr): 3243, 1650, 1591, 1574 cm^−1^; ^1^H-NMR (DMSO-*d*_6_) δ:13.07 (s, 1H), 8.235 (s, 1H), 7.97 (s, 1H), 7.59 (d, 1H, *J* = 8.8 Hz), 7.36–7.42 (m, 3H), 7.27–7.31 (m, 4H), 7.13–7.16 (m, 2H), 6.95–6.97 (t, 1H, *J* = 5.6 Hz), 6.76 (d, 1H, *J* = 8.0 Hz), 6.25 (s, 1H), 2.37(s, 3H), 1.27 (s, 9H); MS (ESI): *m*/*z* 519.23 [M + Na]^+^. Mp 197.1–199.6 °C.

*1-(2-((1H-Indazol-5-yl)oxy)phenyl)-3-(3-(tert-butyl)-1-(4-chlorophenyl)-1H-pyrazol-5-yl)urea* (**29e**). White solid (3.20 g, 63%). HPLC purity 95.9% (*t*_R_ = 11.24 min). IR (KBr): 3252, 1671, 1600, 1580 cm^−1^; ^1^H-NMR (DMSO-*d*_6_) δ: 13.08 (s, 1H), 8.33 (s, 1H), 7.97 (s, 1H), 7.53 (d, 1H, *J* = 8.8 Hz), 7.47–7.50 (m, 4H), 7.26–7.31 (m, 3H), 7.12–7.16 (m, 2H), 6.92–6.94 (t, 1H, *J* = 5.6 Hz), 6.76 (d, 1H, *J* = 8.0 Hz), 6.23 (s, 1H), 1.26 (s, 9H); MS (ESI): *m*/*z* 523.17 [M + Na]^+^.

*1-(2-((1H-Indazol-5-yl)oxy)phenyl)-3-(1-(4-bromophenyl)-3-(tert-butyl)-1H-pyrazol-5-yl)urea* (**29f**). White solid (2.61 g, 48%). HPLC purity 97.7% (*t*_R_ = 11.08 min). IR (KBr): 3250, 1667, 1602, 1579 cm^−1^; ^1^H-NMR (DMSO-*d*_6_) δ: 13.06 (s, 1H), 8.34 (s, 1H), 7.94 (s, 1H), 7.54 (d, 1H, *J* = 8.8 Hz), 7.46–7.52 (m, 4H), 7.24–7.27 (m, 3H), 7.13–7.16 (m, 2H), 6.93–6.95 (t, 1H, *J* = 5.6 Hz), 6.74 (d, 1H, *J* = 8.0 Hz), 6.24 (s, 1H), 1.25 (s, 9H); MS (ESI): *m*/*z* 567.17 [M + Na]^+^. Mp 200.8.6–202.3 °C.

*1-(2-((1H-Indazol-5-yl)oxy)phenyl)-3-(3-(tert-butyl)-1-(4-fluorophenyl)-1H-pyrazol-5-yl)urea* (**29g**). White solid (2.66 g, yield 55%). HPLC purity 96.6% (*t*_R_ = 11.14 min). IR (KBr): 3250, 1675, 1595, 1581 cm^−1^; ^1^H-NMR (DMSO-*d*_6_) δ: 13.05 (s, 1H), 8.35 (s, 1H), 7.99 (s, 1H), 7.54 (d, 1H, *J* = 8.8 Hz), 7.48–7.52 (m, 4H), 7.27–7.32 (m, 3H), 7.13–7.16 (m, 2H), 6.95–6.97 (t, 1H, *J* = 5.6 Hz), 6.74 (d, 1H, *J* = 8.0 Hz), 6.25 (s, 1H), 1.25(s, 9H); MS (ESI): *m*/*z* 507.20 [M + Na]^+^. Mp 189.6–191.7 °C.

*1-(2-((1H-Indazol-5-yl)oxy)phenyl)-3-(3-(tert-butyl)-1-(4-nitrophenyl)-1H-pyrazol-5-yl)urea* (**29h**). White solid (3.42 g, 67%). HPLC purity 98.9% (*t*_R_ = 11.40 min). IR (KBr): 3247, 1673, 1591, 1579 cm^−1^; ^1^H-NMR (DMSO-*d*_6_) δ: 13.09 (s, 1H), 8.60 (s, 1H), 8.33 (d, 2H, *J* = 8.8 Hz), 8.03 (s, 1H), 7.85 (d, 2H, *J* = 8.8 Hz), 7.56 (d, 1H, *J* = 8.8 Hz), 7.24–7.28 (m, 3H), 7.09–7.12 (m, 3H), 6.75 (d, 1H, *J* = 8.0 Hz), 6.33 (s, 1H), 1.24 (s, 9H); MS (ESI): *m*/*z* 534.09 [M + Na]^+^. Mp 193.2–195.5 °C.

*4-(5-(3-(2-((1H-Indazol-5-yl)oxy)phenyl)ureido)-3-(tert-butyl)-1H-pyrazol-1-yl)benzenesulfonamide* (**29i**). White solid (3.59 g, yield 66%). HPLC purity 97.3% (*t*_R_ = 9.96 min). IR (KBr): 3255, 1668, 1593, 1577 cm^−1^; ^1^H-NMR (DMSO-*d*_6_) δ: 13.07 (s, 1H), 8.44 (s, 1H), 8.01 (s, 1H), 7.93 (d, 1H, *J* = 8.8 Hz), 7.73 (d, 2H, *J* = 8.8 Hz), 7.54 (d, 1H, *J* = 8.8 Hz), 7.48 (s, 2H), 7.28–7.31 (m, 3H), 7.15–7.18 (m, 2H), 6.96–7.01 (m, 1H), 6.75(d, 1H, *J* = 6.4 Hz), 6.34(s, 1H), 4.78 (s, 1H), 1.25(s, 9H); MS (ESI): *m*/*z* 568.18 [M + Na]^+^. Mp 199.2–201.4 °C.

#### 3.2.16. General Method for the Synthesis of **30a**–**30d**

A solution of the corresponding **3a**–**3i** (1.1 equiv.) and **16** (1 equiv.) was stirred 2 h at 90 °C in the presence of Et_3_N (1 mL) and DMSO (50 mL). Then the mixture was poured into ice water and the solution was extracted with EtOAc. The organic layer was washed with water and brine, dried (MgSO_4_), filtered and evaporated to dryness. The product was separated by column chromatography using DCM/MeOH (80:1) as eluent.

*1-(3-(tert-Butyl)-1-(4-chlorophenyl)-1H-pyrazol-5-yl)-3-(2-(2-morpholinoethoxy)benzyl)urea* (**30a**) From **3e** (4.23 g, 10 mmol), and **16** (2.36 g, 10 mmol), after work-up and purification **30a** (3.83 g, 75%) was obtained as a white solid. HPLC purity 98.4% (*t*_R_ = 8.27 min). IR (KBr) 3254, 1669, 1596, 1578 cm^−1^; ^1^H-NMR (DMSO-*d*_6_) δ: 8.32 (s, 1H), 7.53–7.56 (m, 4H), 7.21–7.24 (m, 1H), 7.10 (dd, 1H, *J* = 1.6, 8.0 Hz), 6.98 (d, 1H, *J* = 8.0 Hz), 6.89–6.93 (m, 1H), 6.73–6.75 (t, 1H, *J* = 5.6 Hz), 6.27 (s, 1H), 4.19 (d, 2H, *J* = 5.6), 4.09–4.11 (t, 2H, *J* = 5.6 Hz), 3.55–3.57 (t, 4H, *J* = 4.8 Hz), 2.70–2.72 (t, 2H, *J* = 5.6 Hz), 2.50–2.52 (t, 4H, *J* = 4.0 Hz), 1.25 (s, 9H); MS (ESI): *m*/*z* 534.24 [M + Na]^+^. Mp 163.6–165.7 °C.

*1-(3-(tert-Butyl)-1-(p-tolyl)-1H-pyrazol-5-yl)-3-(2-(2-morpholinoethoxy)benzyl)urea* (**30b**). White solid (2.94 g, 60%). HPLC purity 95.6% (*t*_R_ = 8.16 min). IR (KBr): 3257, 1668, 1595, 1575 cm^−1^; ^1^H-NMR (DMSO-*d*_6_) δ: 8.17 (s, 1H), 7.35 (d, 2H, *J* = 8.8 Hz), 7.21–7.26 (m, 1H), 7.11 (d, 1H, *J* = 8.0 Hz), 7.03 (d, 2H, *J* = 8.8 Hz), 6.98 (d, 1H, *J* = 8.0 Hz), 6.89–6.91 (t, 1H, *J* = 7.6 Hz), 6.74–6.76 (t, 1H, *J* = 5.6 Hz), 6.24 (s, 1H), 4.20 (d, 2H, *J* = 5.6 Hz), 4.09–4.11 (t, 2H, *J* = 5.6 Hz), 3.80 (s, 3H), 3.55–3.57 (t, 4H, *J* = 4.8 Hz), 2.71–2.73 (t, 2H, *J* = 5.6 Hz), 2.50–2.52 (t, 4H, *J* = 5.6 Hz), 1.24 (s, 9H). MS (ESI): *m*/*z* 514.24 [M + Na]^+^. Mp 171.1–173.7 °C.

*4-(3-(tert-Butyl)-5-(3-(2-(2-morpholinoethoxy)benzyl)ureido)-1H-pyrazol-1-yl)benzenesulfonamide* (**30c**). White solid (3.05 g, 55%). HPLC purity 97.2% (*t*_R_ = 7.49 min). IR (KBr) 3259, 1670, 1591, 1571 cm^−1^; ^1^H-NMR (DMSO-*d*_6_) δ: 8.46 (s, 1H), 7.90 (d, 2H, *J* = 8.0 Hz), 7.69 (d, 2H, *J* = 8.0 Hz), 7.48 (s, 2H), 7.22–7.25 (m, 1H), 7.12 (d, 1H, *J* = 6.4 Hz), 6.98 (d, 1H, *J* = 8.0 Hz), 6.91–6.93 (t, 1H, *J* = 8.0 Hz), 6.78–6.80 (t, 1H, *J* = 5.6 Hz), 4.20 (d, 2H, *J* = 5.6 Hz), 4.10 (t, 2H, *J* = 5.6 Hz), 3.55–3.57 (t, 4H, *J* = 4.8 Hz), 2.71–2.73(t, 2H, *J* = 5.6 Hz), 2.50–2.52 (t, 4H, *J* = 5.6 Hz), 1.26 (s, 9H); MS (ESI): *m*/*z* 579.25 [M + Na]^+^. Mp 159.5–161.9 °C.

*1-(3-(tert-Butyl)-1-phenyl-1H-pyrazol-5-yl)-3-(2-(2-morpholinoethoxy)benzyl)urea* (**30d**). White solid (2.48 g, 52%). HPLC purity 97.2% (*t*_R_ = 8.11 min). IR (KBr): 3261, 1671, 1593, 1574 cm^−1^; ^1^H-NMR (DMSO-*d*_6_) δ: 8.29 (s, 1H), 7.48–7.52 (m, 4H), 7.40–7.44 (m, 1H), 7.21–7.25 (m, 1H), 6.98 (d, 1H, *J* = 8.0 Hz), 6.89–6.92 (t, 1H, *J* = 7.6 Hz), 6.76–6.79 (t, 1H, *J* = 5.6 Hz), 6.28 (s, 1H), 4.20–4.22 (d, 2H, *J* = 5.6 Hz), 4.09–4.11 (t, 2H, *J* = 5.6 Hz), 3.55–3.57 (t, 4H, *J* = 4.8 Hz), 2.70–2.72 (t, 2H, *J* = 5.6 Hz), 2.50–2.52 (t, 4H, *J* = 5.6 Hz), 1.25 (s, 9H); MS (ESI): *m*/*z* 510.27 [M + Na]^+^. Mp 160.0–162.2 °C.

### 3.3. IC_50_ Determination of Inhibition of MAPKAPK2 Release from the GFP-MAPKAPk2_BHK Cell Model

The GFP-MAPKAPk2_BHK cell model can differentially express the MAPKAPK2 molecule and GFP information system so the activity can be derived from the contrast of the compound BIRB-796 and the obtained compounds. GFP-MAPKAPk2_BHK cells were cultured in 96-well plates (2.0 × 10^4^/100 μL/well) containing 10% heat-inactivated foetal bovine serum (FBS) and 1 mg/mL G418 at 37 °C in humidified air containing 5% CO_2_ for 18–24 h. The cells were washed with nutrient solution (100 μL/well) and changed to the compound liquid (100 μL/well). Experiments were set up with the control, and the compound was repeated at each concentration in three wells. The cells were cultured in 96-well plates at 37 °C in humidified air containing 5% CO_2_ for 90 min. The cells were washed with the dye solution (20 μL/well), and the dyed cells were held for 1 hour at room temperature in the dye liquid. Analysis of nuclear translocation and calculation of the ability of the compounds to inhibit nuclear translocation was performed using the IN Cell Analyzer 1000 Nuclear Trafficking Analysis Module System (GE, Madison, WI, USA). The cultured cells were treated with the obtained compounds in seven concentration grads (1, 3, 10 and 30 nM, 0.1 and 0.3 μM). The inhibitory percentage can be calculated using the amounts of cells after the disposition of the different compounds.

### 3.4. IC_50_ Determination of Inhibition of TNF-α Release from THP-1 Cells after LPS Stimulation

THP-1 cells were cultured in RPMI medium 1640 with 10% (*v*/*v*) foetal bovine serum (FBS), 100 U/mL penicillin, 100 μg/mL streptomycin, and 50 μM 2-mercaptoethanol at 37 °C and 5% CO_2_. LPS stock was prepared at 1 mg/mL by adding 1 mL sterile endotoxin-free water to 1 mg LPS powder. The LPS stock was aliquoted and stored at −20 °C. Test medium: RPMI Medium 1640 with 0.5% (*v*/*v*) foetal bovine serum, 100 U/mL penicillin, 100 μg/mL streptomycin, 50 μM 2-mercaptoethanol. An amount of 100 K THP-1 cells were seeded into a 96-well cell culture plate in 100 μL of test medium. The compounds were diluted at 1:3.5 in DMSO over 10 points (9 + 0) with a starting concentration of 333 μM. An amount of 3.6 μL of diluted compound was transferred to 96.4 μL test medium, with a starting concentration of 12 μM. A total of 10 μL compound was added to the plate with cells, and the cells were incubated for 1 hour at 37 °C. An amount of 10 μL/well LPS was added to stimulate the cells. The final LPS concentration was 300 ng/mL, and the DMSO concentration was 0.3%. Final compound concentrations: 1000.00, 285.71, 81.63, 23.32, 6.67, 1.90, 0.54, 0.16, 0.04 and 0 nM. Two replicates were used for each dose. The plate was incubated for 5 hours at 37 °C. The TNF-α standard curve samples were prepared with final concentrations of 2500, 1250, 625, 312, 156, 78, 39, 19.5, 9.75 and 0 pg/mL. Three replicates were used for each dose. At the 5th hour, the TNF-α in the supernatants and standard curve samples was determined using the HTRF kit. The cells were centrifuged at 2000 rpm for 5 min, and 10 μL supernatant was added to a ProxiPlate-384 Plus assay plate (PerkinElmer). Amounts of 5 μL Anti-TNF-α-Cryptate (Invitrogen, Carlsbad, CA, USA) and 5 μL Anti-TNFα-XL665 (Invitrogen) were added, and the plate was incubated at RT for 15 h. The plate was read on an Envision instrument (PerkinElmer) and the IC_50_ was calculated.

### 3.5. p38α MAP Kinase Activity Assessment Based on the Rate of Phosphorylation of ATF-2 (Activation Transcription Factor 2) in an in Vitro Assay

Kinase reaction buffer was composed of 50 mM HEPES (pH 7.5), 0.01% BRIJ-35, 10 mM MgCl_2_ and 1 mM EGTA. Prepared MAPK14/*p*38α in 1× kinase buffer with concentration at 500 ng/mL. A 2-fold serial dilution was performed using 1× kinase buffer from 500 ng/mL using 16 dose points. The serially diluted MAPK14/*p*38α (5 μL) was added into the 384-well plate in triplicate and 0.8 μM substrate GFP-ATF2 (19–96) and 180 μM ATP in 1× kinase reaction buffer were prepared (1 mL). The reaction was started by adding 5 μL of the GFP-ATF2(19–96) substrate and ATP solution into each well of the assay plate. The final starting concentration of MAPK14/*p*38α was 250ng/ml, and the final GFP-ATF2 and ATP concentrations were 0.4 μM and 90 μM, respectively. The assay plate was sealed and incubated for 1 hour at room temperature (RT). Antibody solution (1 mL) was prepared from 20 mM EDTA and 4 nM Tb-antipATF2 (pThr71) antibody in TR-FRET dilution buffer. Antibody solution (10 μL) was added into each well of the assay plate and mixed gently. The final EDTA concentration was 10 mM, and the final Tb-antipATF2 (pThr71) concentration was 2 nM. The assay plate was sealed and incubated for 30 minutes at RT. Finally, the TR-FRET signal was readed on the Envision 2104 plate reader.

Inhibitor in 0.5% DMSO (2 μL/well) at 5-fold the final assay concentration was added into a 384-well assay plate. For the first inhibitor screening cycle, the final concentrations of inhibitors were 3333, 1111, 370, 123, 41, 13.7, 4.57, 1.52, 0.51, 0.17 and 0.056 nM (3-fold dilution, 11 dose points, two replicates for each dose). The inhibitor concentration was then adjusted according to the first cycle result. MAPK14/*p38*α (4 μL/well) was added to each well of the 384-well assay plate and incubated for 15 min at RT. To start the reaction, substrate GFP-ATF2 (19–96) and ATP (4 μL/well) were added into kinase reaction buffer. The TR-FRET signal was read on the Envision 2104 plate reader. The resulting TR-FRET emission ratio was plotted against the concentration of inhibitor, and the data fitted to a sigmoidal dose-response curve with a variable slope. The IC_50_ concentration was calculated from the curve.

### 3.6. Binding Experiments

The Biacore instrument uses a technique based on surface plasmon resonance to follow the real-time binding of compounds to immobilized proteins on a carboxymethyl dextran surface in a liquid milieu. Compounds were stored as 10 mM stock solutions in 100% dimethyl sulfoxide (DMSO). All light-sensitive compounds were handled under yellow protective light. After dilution to 1 mM with 100% DMSO, samples were mixed with 1.03- to 1.05-fold concentrated assay buffer and DMSO to yield 10 µM compound in a final buffer composition of 50 mM Tris (pH 7.5), 150 mM NaCl, 10 mM MgCl_2_, 1 mM MnCl_2_, and 3% or 5% DMSO (assay buffer), which was also used as the instrument running buffer and sample dilution buffer. ATP was dissolved directly in running buffer to make a 10 mM solution. The run was started with five start-up cycles in which running buffer was injected (instead of a sample) followed by sample injection cycles. Zero concentration samples were used as blanks. A typical analysis cycle consisted of a 60 s sample injection (30 μL/min), 120–200 s of buffer flow (dissociation phase), followed by a needle and tubing wash with 50% DMSO and buffer, and finally, a 30 s buffer injection to check for sample carry over. The flow cell temperature was 25 °C. Between sample series, a solvent correction cycle was run according to the instrument manual to adjust for referencing errors due to refractive index mismatches between running buffer and samples [35]. These data were used to calculate the KD for compounds.

## 4. Conclusions

In summary, we have designed and synthesized a novel series of substituted *N*,*N*′-diarylurea compounds as potential *p38*α inhibitors and showed that some compounds possessed good inhibitory potencies. In order to obtain these target compounds, we designed a number of routes to synthesize the targets. The target compounds were evaluated for the inhibitory activity against *p38*α, MAPKAPK2 in BHK cells, TNF-α release in LPS-stimulated THP-1 cells and *p38*α binding experiments. A promising overall profile led to the nomination of compound **25a** as a *p38*α inhibitor development candidate. Further information related to the research and development of compound **25a** will be reported in due course.

## Figures and Tables

**Table 1:** MAPKAPK2, *p38*α and TNF-α activity data for small molecule inhibitors.

Compounds	R_1_	R_2_	MAPKAPK2 IC_50_ (nM)	P38α IC_50_ (nM)	TNF-α IC_50_ (nM)
BIRB-796	—		18.01	13.74	n.d.^a^
SB203580	—	—	n.d.	6.96 ± 1.14	800
**25a**	4-methyl	H	1.36 ± 0.31	0.47 ± 0.02	0.73 ± 0.13
**25b**	H	H	2.45 ± 0.59	0.80 ± 0.03	1.00 ± 0.09
**25c**	3-methyl	H	6.60 ± 0.66	0.77 ± 0.08	1.06 ± 0.05
**25d**	4-methoxy	H	5.45 ± 0.43	1.22 ± 0.32	1.43 ± 0.07
**25e**	4-chloro	H	9.92 ± 0.65	n.d.	3.48 ± 0.46
**25f**	4-bromo	H	7.40 ± 0.87	0.73 ± 0.01	1.82 ± 0.15
**25g**	4-fluoro	H	3.16 ± 1.31	0.66 ± 0.09	2.00 ± 0.01
**25h**	4-nitro	H	2.64 ± 0.56	0.82 ± 0.01	1.79 ± 0.48
**25i**	4-nitro	F	3.83 ± 1.12	1.43 ± 0.08	n.d.
**25j**	4-methyl	F	3.61 ± 2.43	1.53 ± 0.07	n.d.
**25k**	4-sulfamoyl	H	38.98 ± 5.76	n.d.	8.69 ± 0.01
**26a**	4-methyl	—	60.72 ± 2.31	n.d.	n.d.
**26b**	H	—	22.32 ± 1.88	5.14 ± 0.33	n.d.
**26c**	4-sulfamoyl	—	245.68 ± 2.09	n.d.	n.d.
**26d**	4-methoxy	—	69.83 ± 2.08	n.d.	n.d.
**26e**	4-fluoro	—	37.99 ± 1.24	5.96 ± 0.01	n.d.
**26f**	4-nitro	—	41.20 ± 1.46	n.d.	n.d.
**27a**	4-methyl	H	133.41 ± 0.07	3.59 ± 0.21	15.31 ± 2.56
**27b**	4-nitro	H	98.52 ± 5.89	3.87 ± 0.43	27.54 ± 1.21
**27c**	H	H	124.37 ± 6.64	3.73 ± 0.32	18.37 ± 4.35
**27d**	4-nitro	F	n.d.	5.76 ± 0.24	130.35 ± 3.33
**27e**	4-methyl	F	n.d.	5.00 ± 0.53	182.37 ± 4.35
**28a**	4-methyl	—	>10 µM	>10 µM	n.d.
**28b**	H	—	>10 µM	>10 µM	n.d.
**28c**	3-methyl	—	>10 µM	n.d.	n.d.
**28d**	4-methoxy	—	>10 µM	n.d.	n.d.
**28e**	4-chloro	—	>10 µM	n.d.	n.d.
**28f**	4-bromo	—	>10 µM	n.d.	n.d.
**28g**	4-fluoro	—	>10 µM	n.d.	n.d.
**28h**	4-nitro	—	>10 µM	n.d.	n.d.
**28i**	4-sulfamoyl	—	>10 µM	n.d.	n.d.
**29a**	4-methyl	—	>10 µM	>10 µM	n.d.
**29b**	H	—	>10 µM	>10 µM	n.d.
**29c**	3-methyl	—	>10 µM	n.d.	n.d.
**29d**	4-methoxy	—	>10 µM	n.d.	n.d.
**29e**	4-chloro	—	>10 µM	n.d.	n.d.
**29f**	4-bromo	—	>10 µM	n.d.	n.d.
**29g**	4-fluoro	—	>10 µM	n.d.	n.d.
**29h**	4-nitro	—	>10 µM	n.d.	n.d.
**29i**	4-sulfamoyl	—	>10 µM	n.d.	n.d.
**30a**	4-chloro	—	>3 µM	n.d.	n.d.
**30b**	4-methyl	—	>3 µM	n.d.	n.d.
**30c**	4-sulfamoyl	—	>3 µM	n.d.	n.d.
**30d**	H	—	>3 µM	n.d.	n.d.

^a^ n.d.: not determined.

**Table 2:** Detailed binding analysis of small molecule inhibitors with *p38*α mitogen-activated protein kinase.

Compounds	KD (mol/L)
BIRB-796	0.1 × 10^−9^
SB203580	6.15 × 10^−8^
**25a**	1.54 × 10^−8^
**25b**	3.82 × 10^−7^
**25c**	4.21 × 10^−7^
**25h**	2.21 × 10^−7^

## Supplementary Materials

Supplementary materials can be accessed at: http://www.mdpi.com/1420-3049/21/5/677/s1.

## References

[1] Lee J.C., Laydon J.T., McDonnell P.C., Gallagher T.F., Kumar S., Green D., McNulty D., Blumenthal M.J., Heys J.R., Landvatter S.W. (1994). A protein kinase involved in the regulation of inflammatory cytokine biosynthesis. Nature.

[2] Rutgeerts P., D’Haens G., Targan S., Vasiliauskas E., Hanauer S.B., Present D.H., Mayer L., van Hogezand R.A., Braakman T., DeWoody K.L. (1999). Efficacy and safety of retreatment with anti-tumor necrosis factor antibody (infliximab) to maintain remission in crohn’s disease. Gastroenterology.

[3] Foster M.L., Halley F., Souness J.E. (2000). Potential of p38 inhibitors in the treatment of rheumatoid arthritis. Drug News Perspect..

[4] Kumar S., Boehm J., Lee J.C. (2003). P38 map kinases: Key signalling molecules as therapeutic targets for inflammatory diseases. Nat. Rev. Drug Discov..

[5] Wagner E.F., Nebreda A.R. (2009). Signal integration by jnk and p38 mapk pathways in cancer development. Nat. Rev. Cancer.

[6] Chung K.F. (2011). P38 mitogen-activated protein kinase pathways in asthma and copd. Chest.

[7] Zhong W., Zhu H., Sheng F., Tian Y., Zhou J., Chen Y., Li S., Lin J. (2014). Activation of the mapk11/12/13/14 (p38 mapk) pathway regulates the transcription of autophagy genes in response to oxidative stress induced by a novel copper complex in hela cells. Autophagy.

[8] Brando Lima A.C., Machado A.L., Simon P., Cavalcante M.M., Rezende D.C., Sperandio da Silva G.M., Nascimento P.G.B.D., Quintas L.E.M., Cunha F.Q., Barreiro E.J. (2011). Anti-inflammatory effects of lassbio-998, a new drug candidate designed to be a p38 mapk inhibitor, in an experimental model of acute lung inflammation. Pharmacol. Rep..

[9] Campbell R.M., Anderson B.D., Brooks N.A., Brooks H.B., Chan E.M., de Dios A., Gilmour R., Graff J.R., Jambrina E., Mader M. (2013). Characterization of ly2228820 dimesylate, a potent and selective inhibitor of p38 mapk with antitumor activity. Mol. Cancer Therap..

[10] MacNee W., Allan R.J., Jones I., de Salvo M.C., Tan L.F. (2013). Efficacy and safety of the oral p38 inhibitor ph-797804 in chronic obstructive pulmonary disease: A randomised clinical trial. Thorax.

[11] Kragholm K., Newby L.K., Melloni C. (2015). Emerging treatment options to improve cardiovascular outcomes in patients with acute coronary syndrome: Focus on losmapimod. Drug Design Dev. Ther..

[12] Newby L.K., Marber M.S., Melloni C., Sarov-Blat L., Aberle L.H., Aylward P.E., Cai G., de Winter R.J., Hamm C.W., Heitner J.F. (2014). Losmapimod, a novel p38 mitogen-activated protein kinase inhibitor, in non-st-segment elevation myocardial infarction: A randomised phase 2 trial. Lancet.

[13] Cirillo P.F., Pargellis C., Regan J. (2002). The non-diaryl heterocycle classes of p38 map kinase inhibitors. Curr. Top. Med. Chem..

[14] Wrobleski S.T., Doweyko A.M. (2005). Structural comparison of p38 inhibitor-protein complexes: A review of recent p38 inhibitors having unique binding interactions. Curr. Top. Med. Chem..

[15] Liu Y., Gray N.S. (2006). Rational design of inhibitors that bind to inactive kinase conformations. Nat. Chem. Biol..

[16] Backes A., Zech B., Felber B., Klebl B., Muller G. (2008). Small-molecule inhibitors binding to protein kinases. Part I: Exceptions from the traditional pharmacophore approach of type I inhibition. Expert Opin. Drug Discov..

[17] Backes A., Zech B., Felber B., Klebl B., Muller G. (2008). Small-molecule inhibitors binding to protein kinase. Part II: The novel pharmacophore approach of type II and type III inhibition. Expert Opin. Drug Discov..

[18] Traxler P., Furet P. (1999). Strategies toward the design of novel and selective protein tyrosine kinase inhibitors. Pharmacol. Therap..

[19] Regan J., Breitfelder S., Cirillo P., Gilmore T., Graham A.G., Hickey E., Klaus B., Madwed J., Moriak M., Moss N. (2002). Pyrazole urea-based inhibitors of p38 map kinase: From lead compound to clinical candidate. J. Med. Chem..

[20] Pargellis C., Tong L., Churchill L., Cirillo P.F., Gilmore T., Graham A.G., Grob P.M., Hickey E.R., Moss N., Pav S. (2002). Inhibition of p38 map kinase by utilizing a novel allosteric binding site. Nat. Struct. Biol..

[21] Regan J., Capolino A., Cirillo P.F., Gilmore T., Graham A.G., Hickey E., Kroe R.R., Madwed J., Moriak M., Nelson R. (2003). Structure-activity relationships of the p38alpha map kinase inhibitor 1-(5-tert-butyl-2-*p*-tolyl-2h-pyrazol-3-yl)-3-[4-(2-morpholin-4-yl-ethoxy)naph-thalen-1-yl]urea (birb 796). J. Med. Chem..

[22] Karcher S.C., Laufer S.A. (2009). Successful structure-based design of recent p38 map kinase inhibitors. Curr. Top. Med. Chem..

[23] Millan D.S., Bunnage M.E., Burrows J.L., Butcher K.J., Dodd P.G., Evans T.J., Fairman D.A., Hughes S.J., Kilty I.C., Lemaitre A. (2011). Design and synthesis of inhaled p38 inhibitors for the treatment of chronic obstructive pulmonary disease. J. Med. Chem..

[24] Zhu D., Li X., Zhong W., Zhao D. (2015). Design, synthesis and biological evaluation of novel substituted *N*,*N*′-diaryl ureas as potent p38 inhibitors. Molecules.

[25] Li X., Zhou X., Zheng Z., Zhong W., Xiao J., Li S. (2009). Short synthesis of 1-(3-tert-butyl-1-phenyl-1*H*-pyrazol-5-yl)-3-(5-(2-morpholinoethoxy)-2*H*-chromen-8-yl) urea derivatives. Synth. Commun..

[26] Vilches-Herrera M., Werkmeister S., Junge K., Börner A., Beller M. (2014). Selective catalytic transfer hydrogenation of nitriles to primary amines using pd/c. Catal. Sci. Technol..

[27] Arai T., Ohno M., Inoue H., Hayashi S., Aoki T., Hirokawa H., Meguro H., Koga Y., Oshida K., Kainoh M. (2012). Design and synthesis of novel p38alpha map kinase inhibitors: Discovery of pyrazole-benzyl ureas bearing 2-molpholinopyrimidine moiety. Bioorg. Med. Chem. Lett..

[28] Bagley M.C., Davis T., Dix M.C., Widdowson C.S., Kipling D. (2006). Microwave-assisted synthesis of *n*-pyrazole ureas and the p38alpha inhibitor birb 796 for study into accelerated cell ageing. Organ. Biomol. Chem..

[29] Getlik M., Grutter C., Simard J.R., Nguyen H.D., Robubi A., Aust B., van Otterlo W.A., Rauh D. (2012). Structure-based design, synthesis and biological evaluation of n-pyrazole, n'-thiazole urea inhibitors of map kinase p38alpha. Eur. J. Med. Chem..

[30] Garcia-Manero G., Khoury H.J., Jabbour E., Lancet J., Winski S.L., Cable L., Rush S., Maloney L., Hogeland G., Ptaszynski M. (2015). A phase i study of oral arry-614, a p38 mapk/tie2 dual inhibitor, in patients with low or intermediate-1 risk myelodysplastic syndromes. Clinical Cancer Res..

[31] Eroglu Z., Stein C.A., Pal S.K. (2013). Targeting angiopoietin-2 signaling in cancer therapy. Expert Opin. Investig. Drugs.

[32] Bachegowda L., Gligich O., Mantzaris I., Schinke C., Wyville D., Carrillo T., Braunschweig I., Steidl U., Verma A. (2013). Signal transduction inhibitors in treatment of myelodysplastic syndromes. J. Hematol. Oncol..

[33] Goldstein D.M., Kuglstatter A., Lou Y., Soth M.J. (2010). Selective p38alpha inhibitors clinically evaluated for the treatment of chronic inflammatory disorders. J. Med. Chem..

[34] Rothman A., Davidson S., Wiencek R.G., Evans W.N., Restrepo H., Sarukhanov V., Ruoslahti E., Williams R., Mann D. (2013). Vascular histomolecular analysis by sequential endoarterial biopsy in a shunt model of pulmonary hypertension. Pulm. Circ..

[35] Nordin H., Jungnelius M., Karlsson R., Karlsson O.P. (2005). Kinetic studies of small molecule interactions with protein kinases using biosensor technology. Anal. Biochem..

