# Supplementary material for: Synthesis and p38 Inhibitory Activity of Some Novel Substituted N,N′-Diarylurea Derivatives
Source: Molecules. 2016 May 23;21(5):677. doi: 10.3390/molecules21050677 (PMC6272846; doi:10.3390/molecules21050677)
Supplement: Supplementary file 1 [file molecules-21-00677-s001.pdf]

# Supplementary Materials: Synthesis and p38 Inhibitory Activity of Some Novel Substituted *N,N'*-Diarylurea Derivatives

**Dianxi Zhu, Qifeng Xing, Ruiyuan Cao, Dongmei Zhao and Wu Zhong**

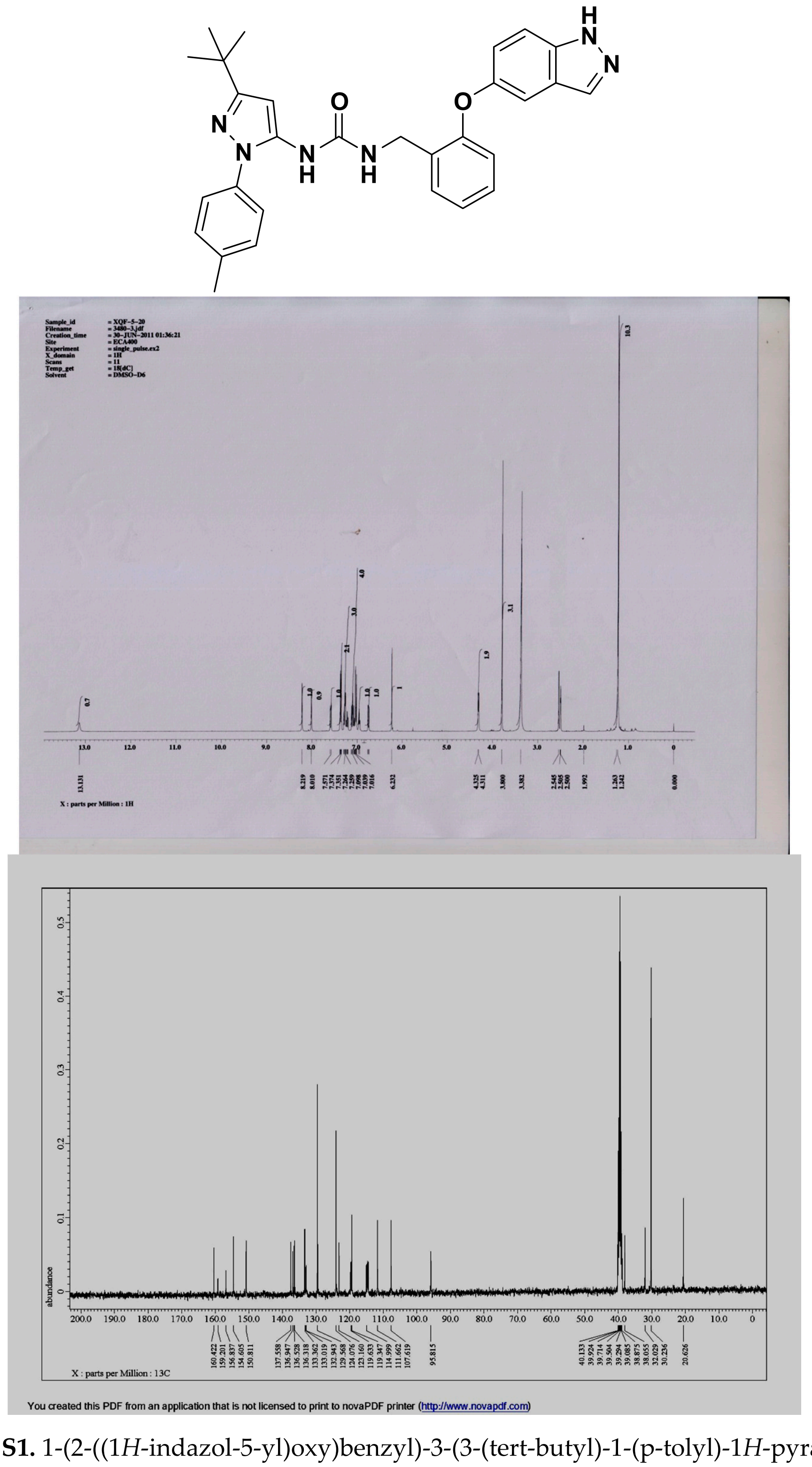


**Figure S1.** 1-(2-((1*H*-indazol-5-yl)oxy)benzyl)-3-(3-(tert-butyl)-1-(p-tolyl)-1*H*-pyrazol-5-yl)urea (**25a**).

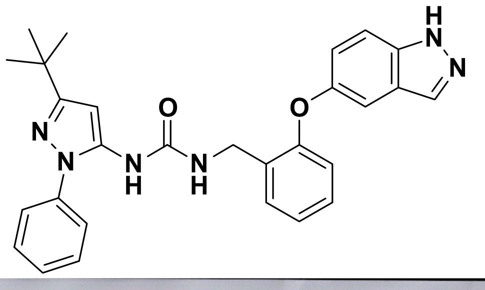

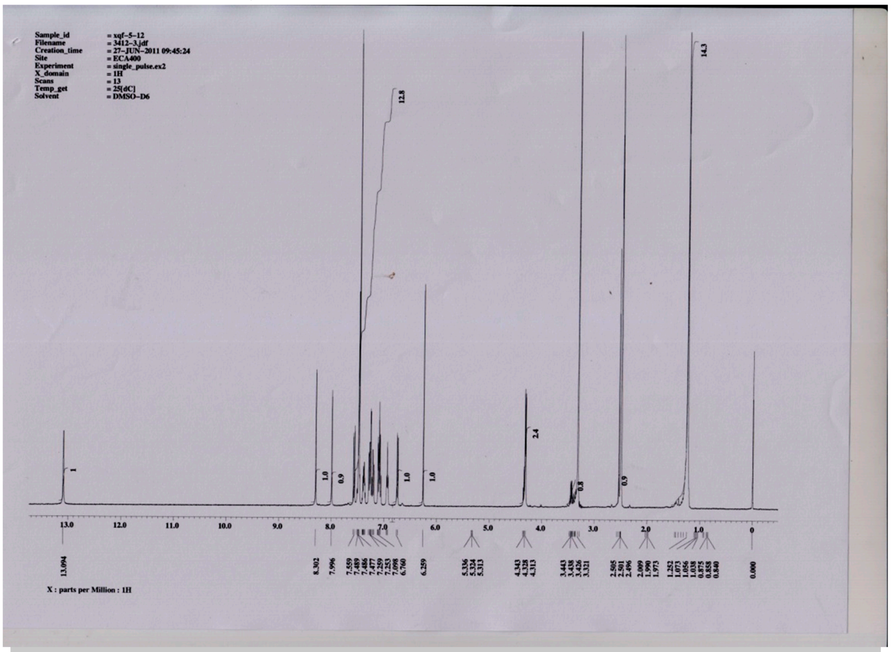

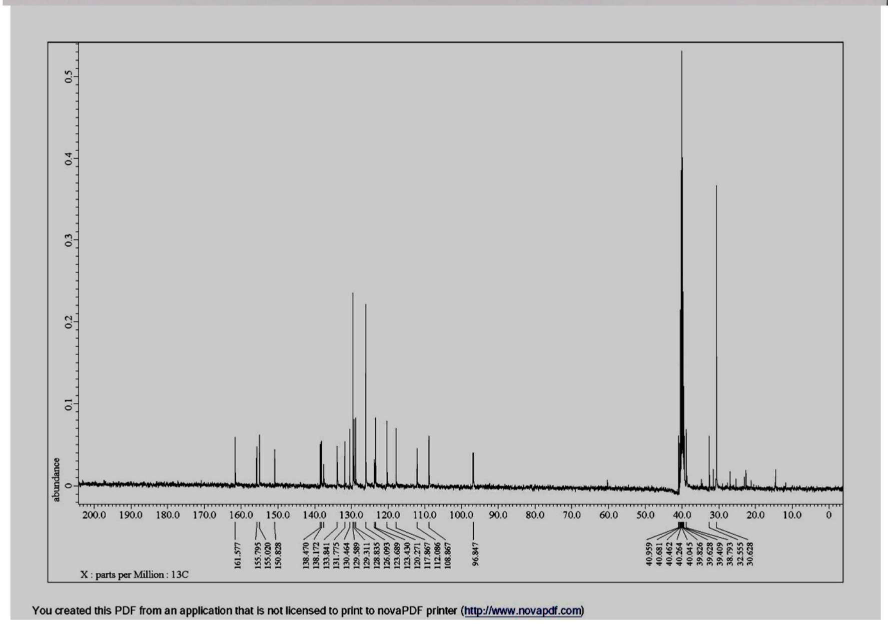


**Figure S2**. 1-(2-((1*H*-indazol-5-yl)oxy)benzyl)-3-(3-(tert-butyl)-1-phenyl-1*H*-pyrazol-5-yl)urea (**25b**).

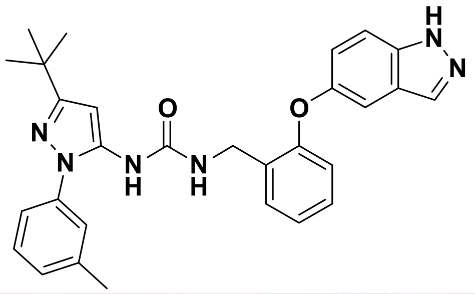

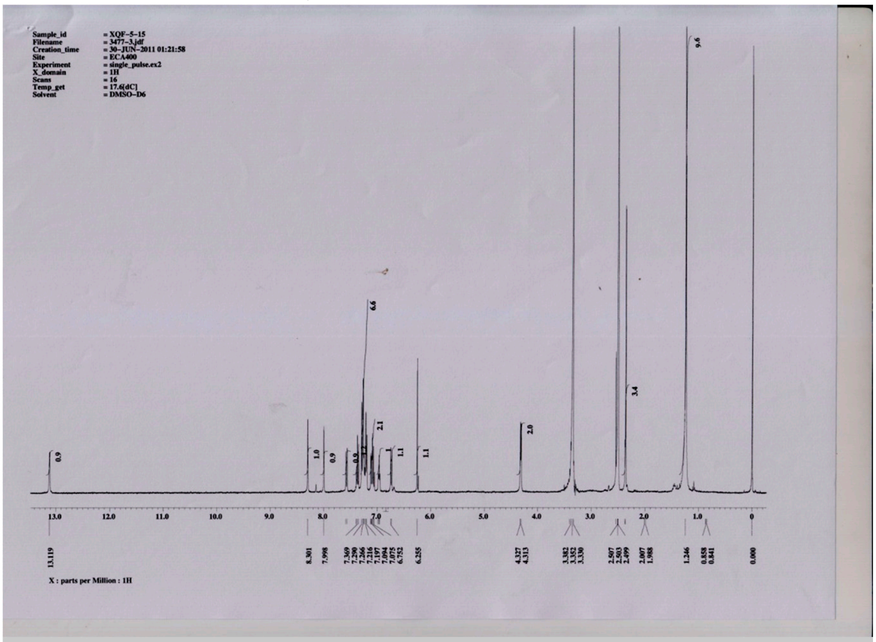


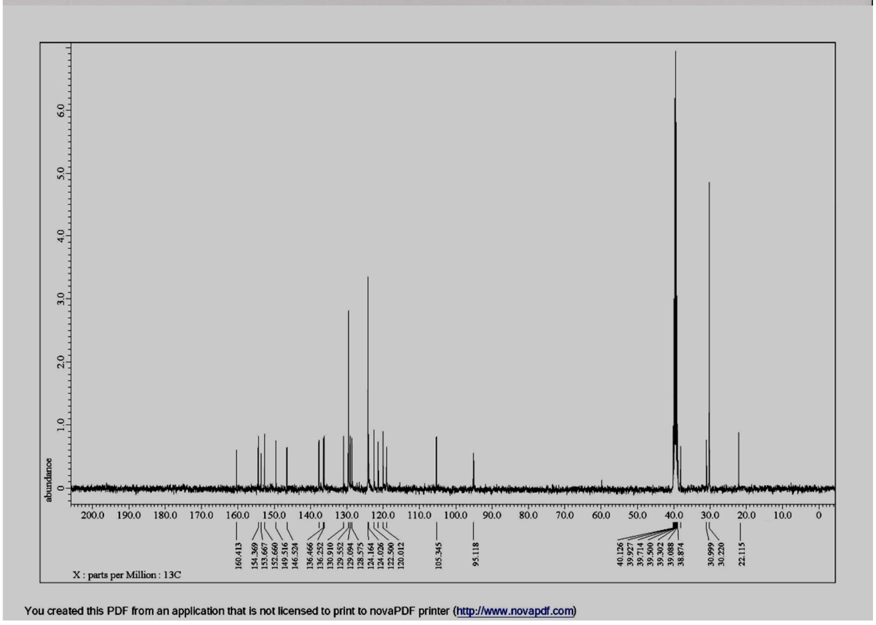


You created this PDF from an application that is not licensed to print to novaPDF printer (http://www.novapdf.com)

**Figure S3.** 1-(2-((1*H*-indazol-5-yl)oxy)benzyl)-3-(3-(tert-butyl)-1-(m-tolyl)-1*H*-pyrazol-5-yl)urea (**25c**).

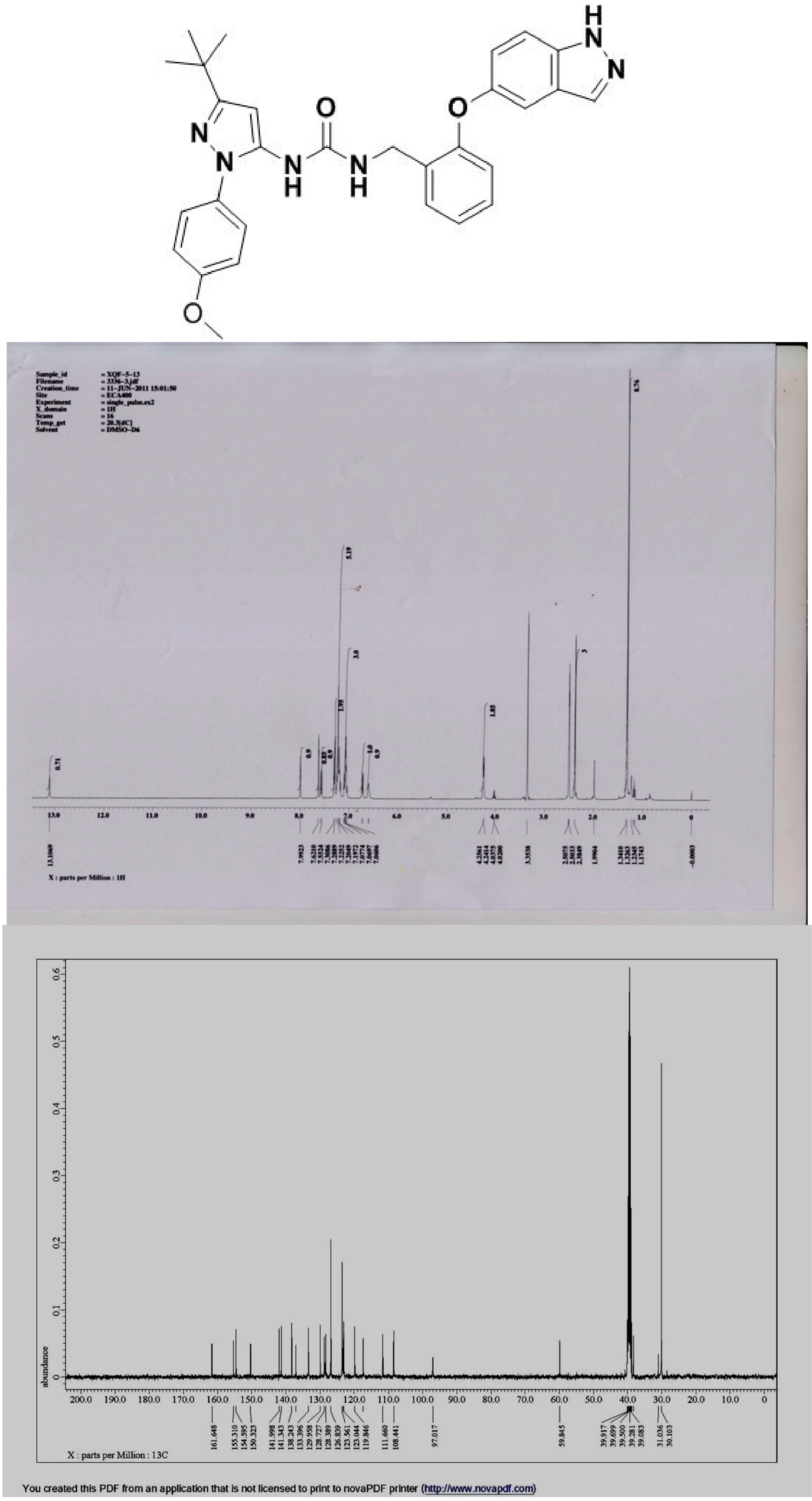


**Figure S4.** 1-(2-((1*H*-indazol-5-yl)oxy)benzyl)-3-(3-(tert-butyl)-1-(4-methoxyphenyl) -1*H*-pyrazol-5-yl)urea (**25d**).

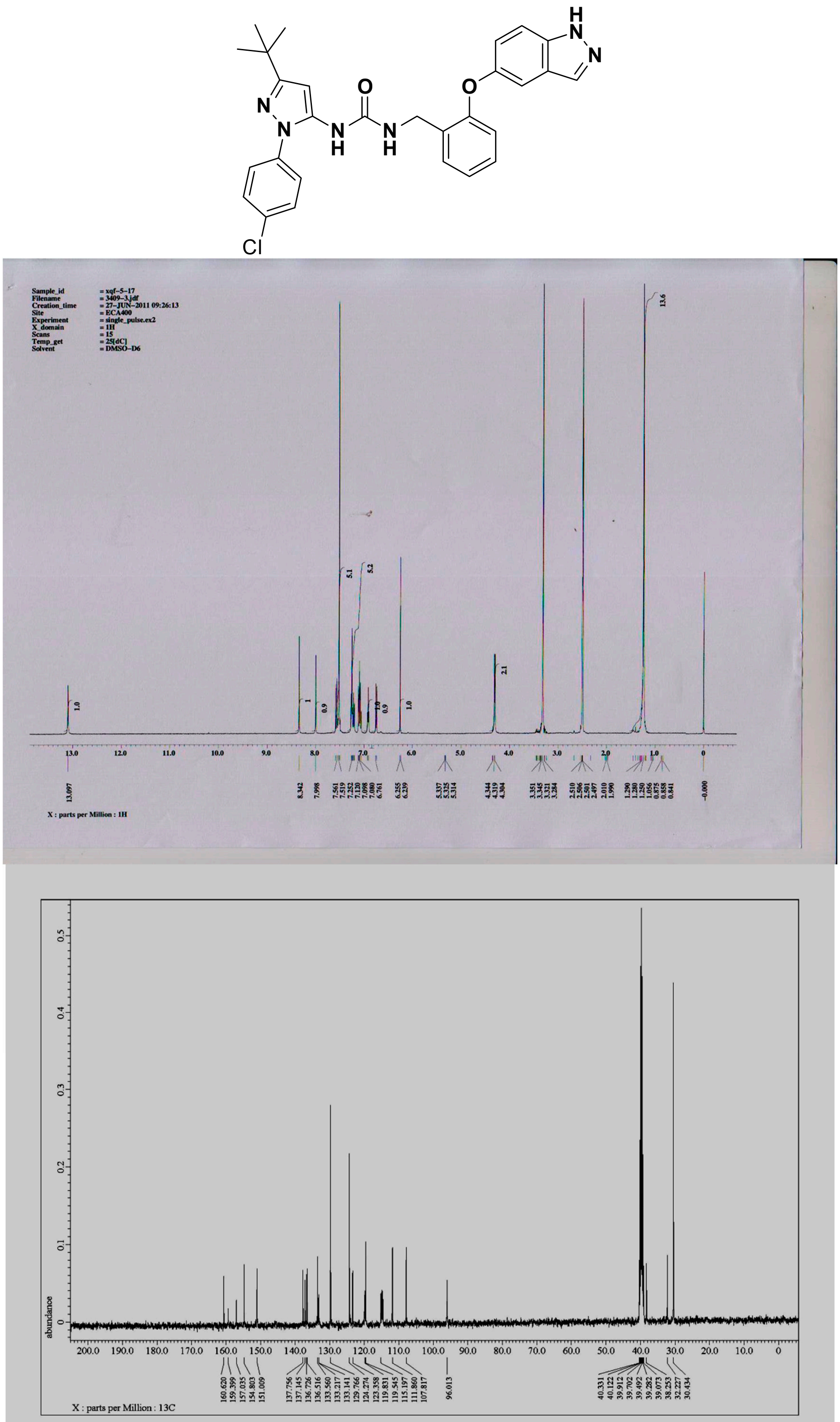


You created this PDF from an application that is not licensed to print to novaPDF printer (http://www.novapdf.com)

**Figure S5.** 1-(2-((1*H*-indazol-5-yl)oxy)benzyl)-3-(3-(tert-butyl)-1-(4-chlorophenyl)-1*H*-pyrazol-5-yl)urea (**25e**).

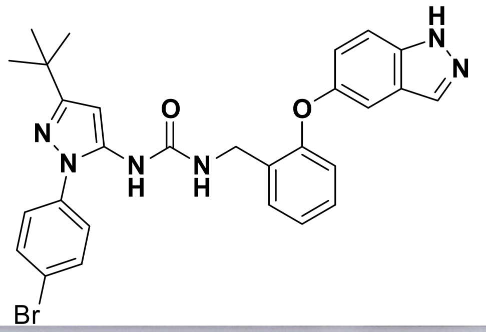


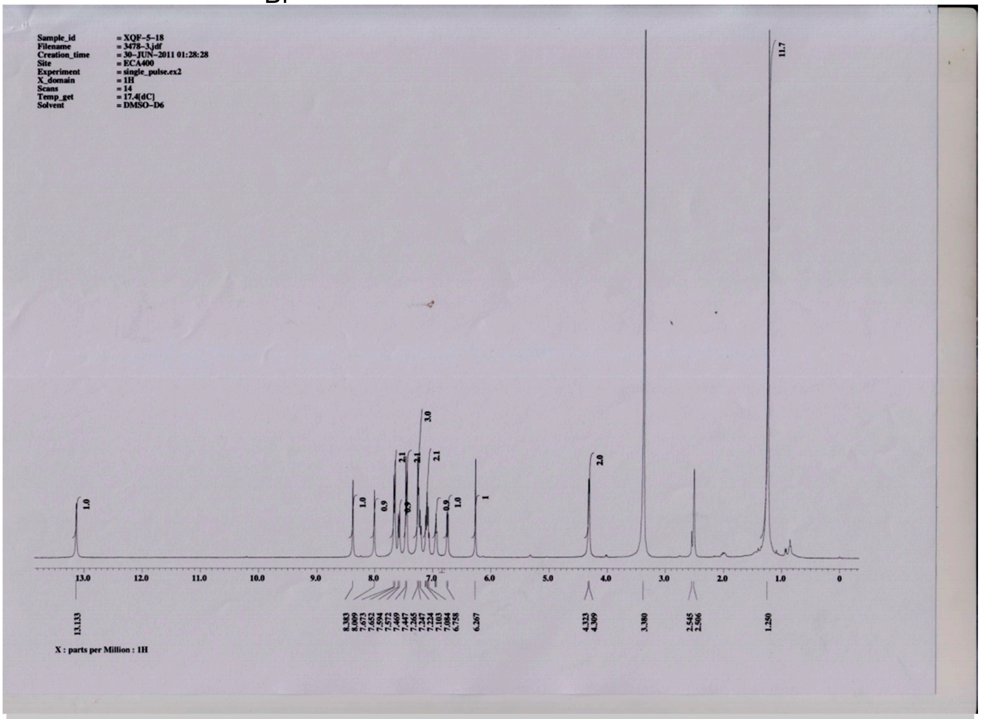


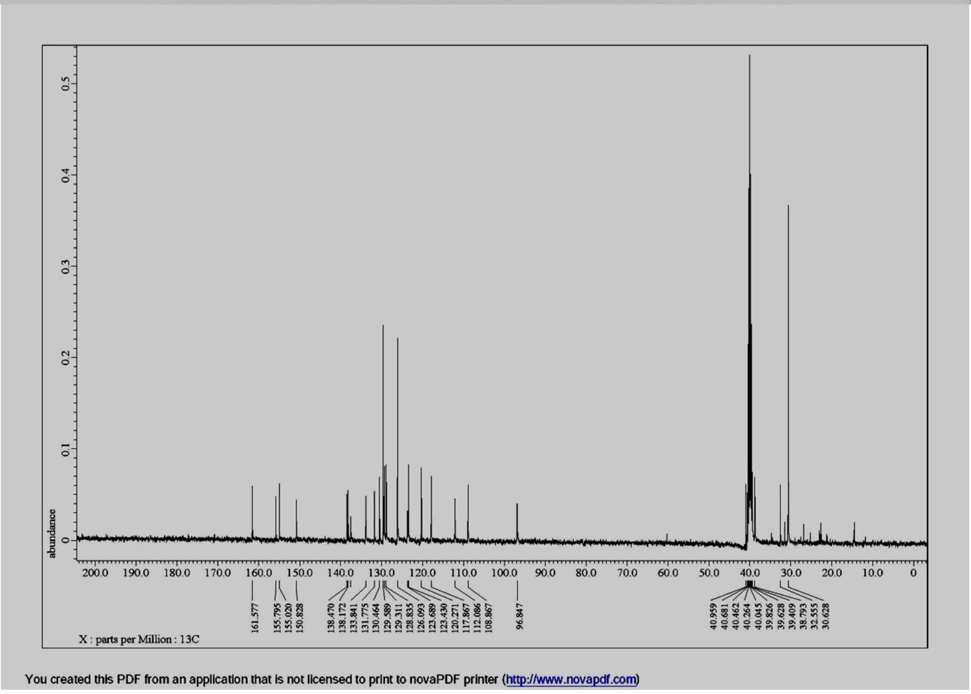


**Figure S6**. 1-(2-((1*H*-indazol-5-yl)oxy)benzyl)-3-(1-(4-bromophenyl)-3-(tert-butyl)-1*H*-pyrazol-5-yl)urea (**25f**).

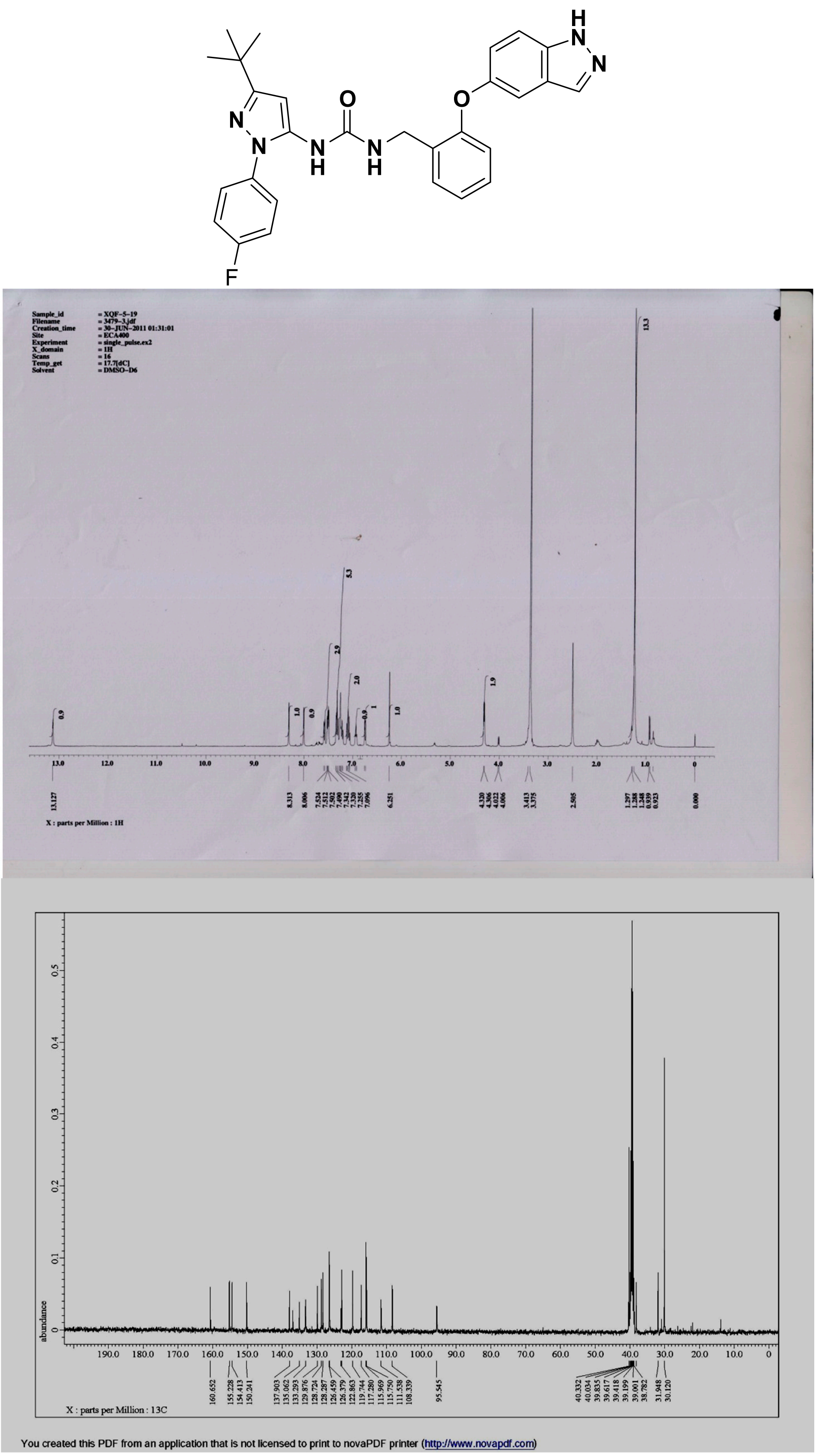


**Figure S7.** 1-(2-((1*H*-indazol-5-yl)oxy)benzyl)-3-(3-(tert-butyl)-1-(4-fluorophenyl)-1*H*-pyrazol-5-yl)urea (**25g**).

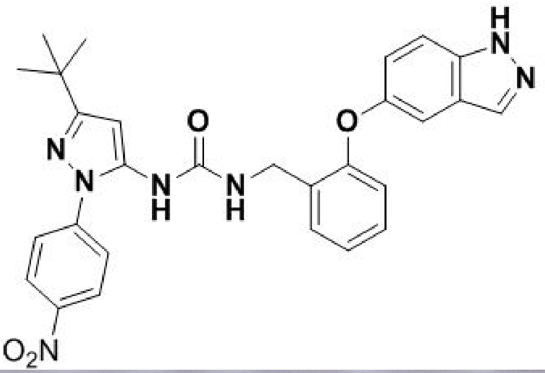

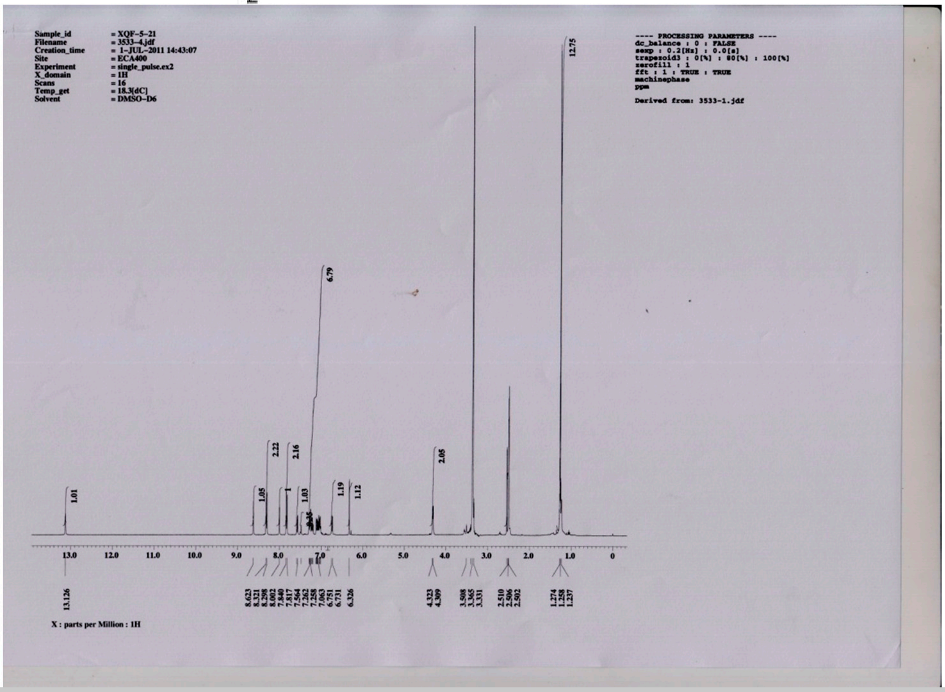

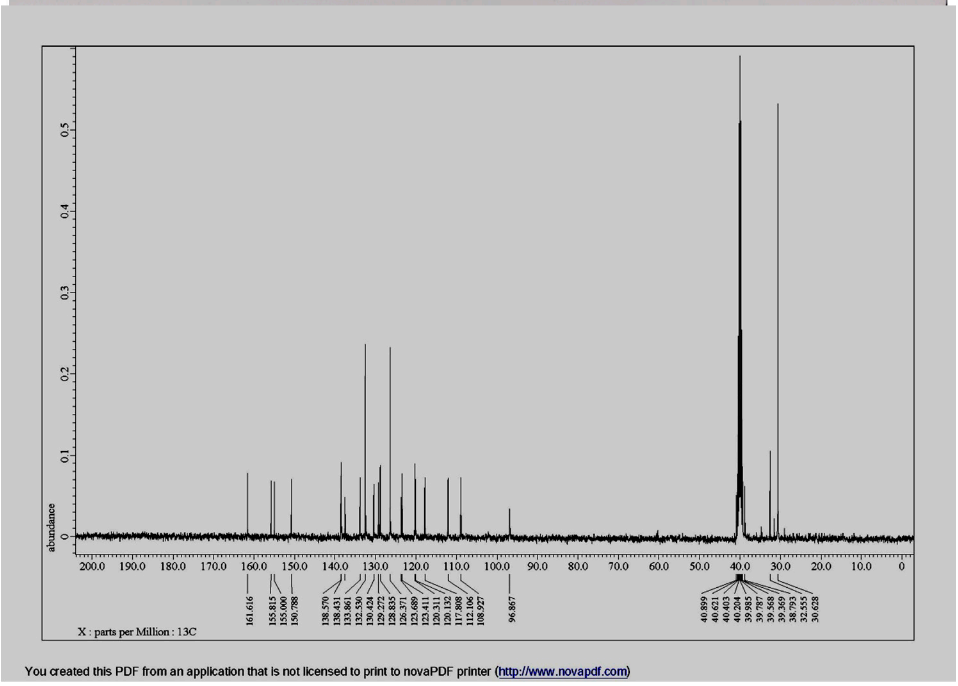


**Figure S8.** 1-(2-((1*H*-indazol-5-yl)oxy)benzyl)-3-(3-(tert-butyl)-1-(4-nitrophenyl)-1*H*-pyrazol-5-yl)urea (**25h**).

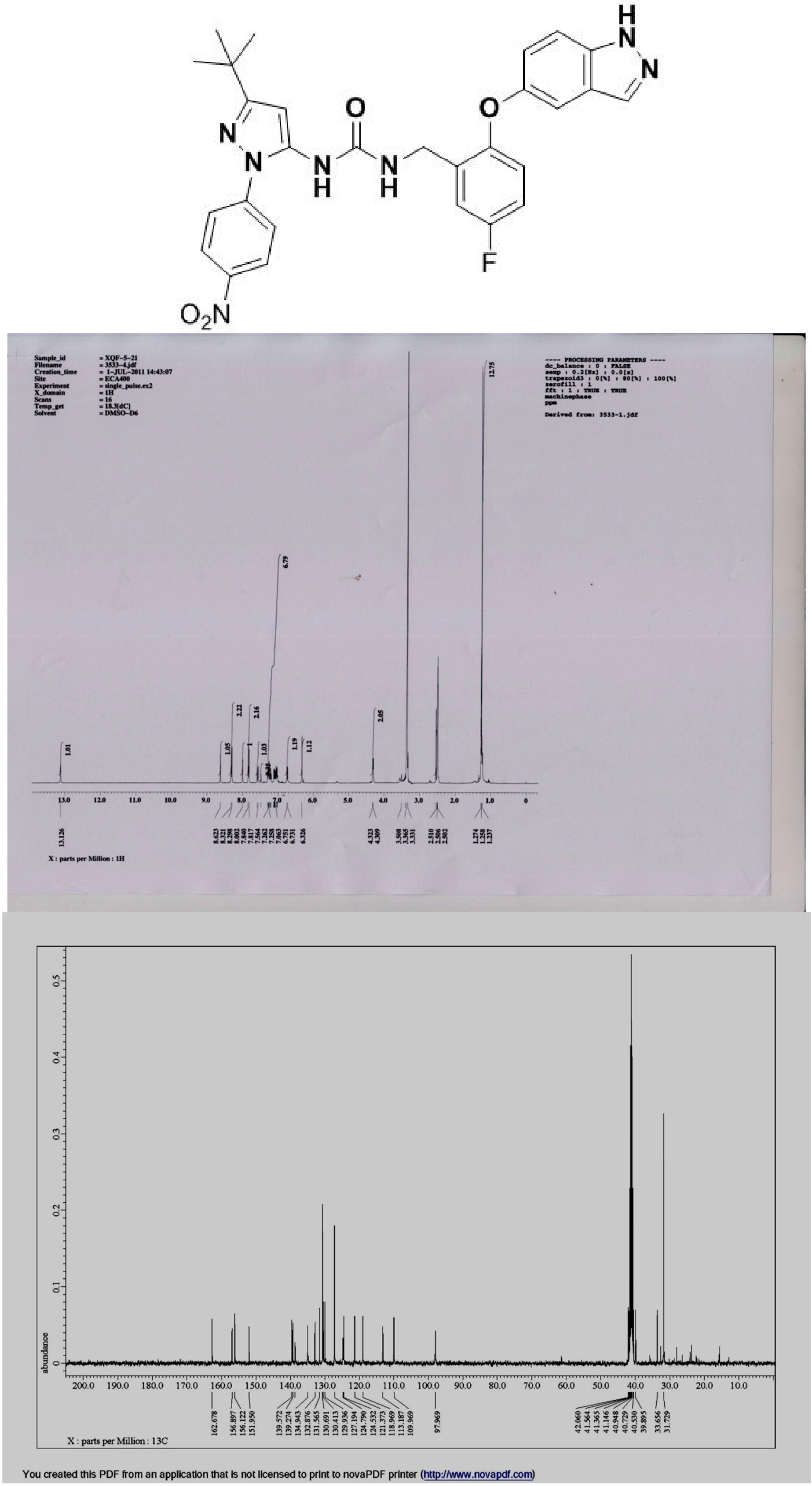


**Figure S9.** 1-(2-((1*H*-indazol-5-yl)oxy)-5-fluorobenzyl)-3-(3-(tert-butyl)-1-(4-nitrophenyl)-1*H*-pyrazol-5-yl)urea(**25i**).

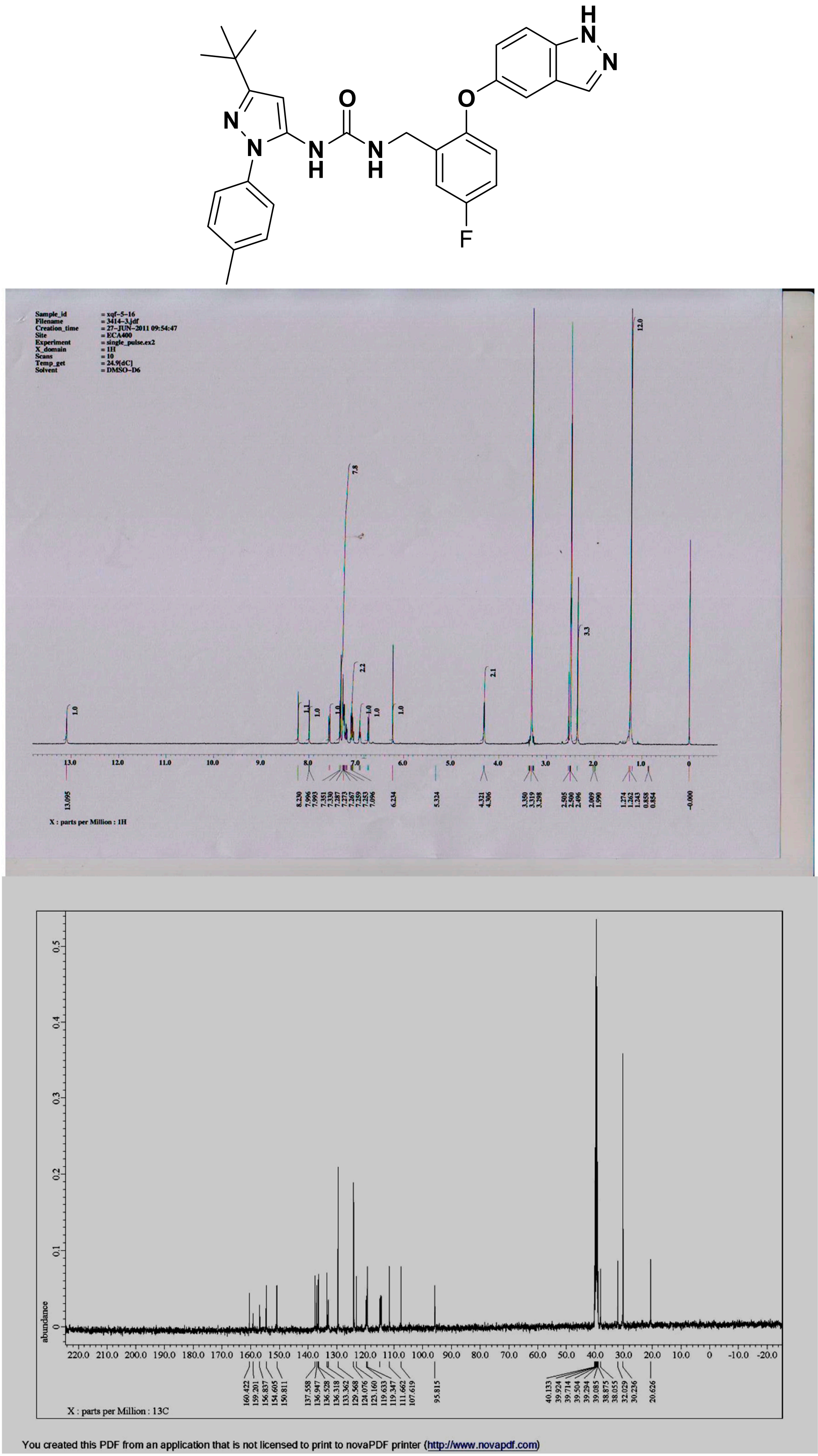


**Figure S10.** 1-(2-((1*H*-indazol-5-yl)oxy)-5-fluorobenzyl)-3-(3-(tert-butyl)-1-(p-tolyl)-1*H*-pyrazol-5-yl)urea (**25j**).

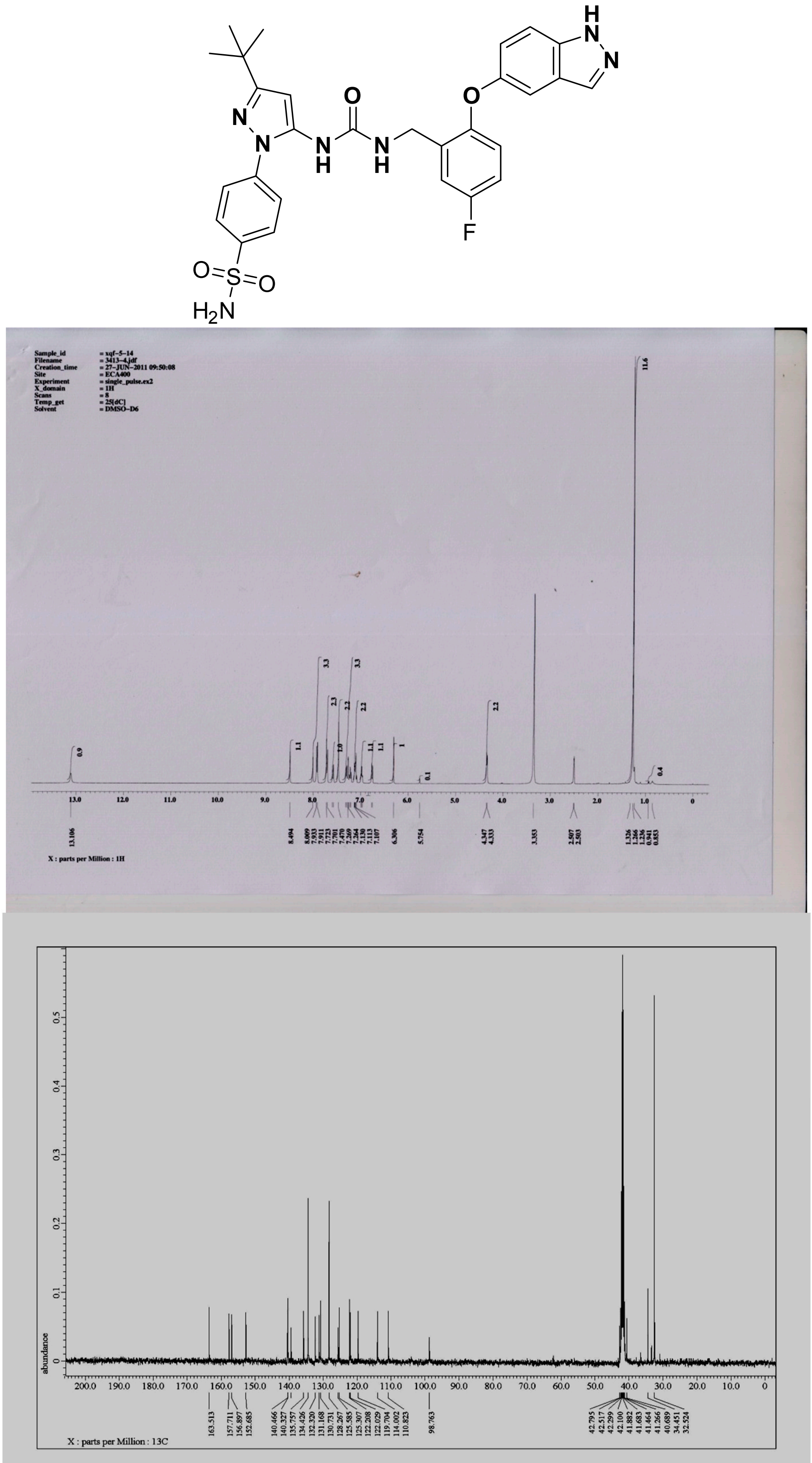

You created this PDF from an application that is not licensed to print to novaPDF printer (http://www.novapdf.com)

**Figure S11.** 4-(5-(3-(2-((1*H*-indazol-5-yl)oxy)benzyl)ureido)-3-(tert-butyl)-1*H*-pyrazol-1-yl)benzenesulfonamide (**25k**).

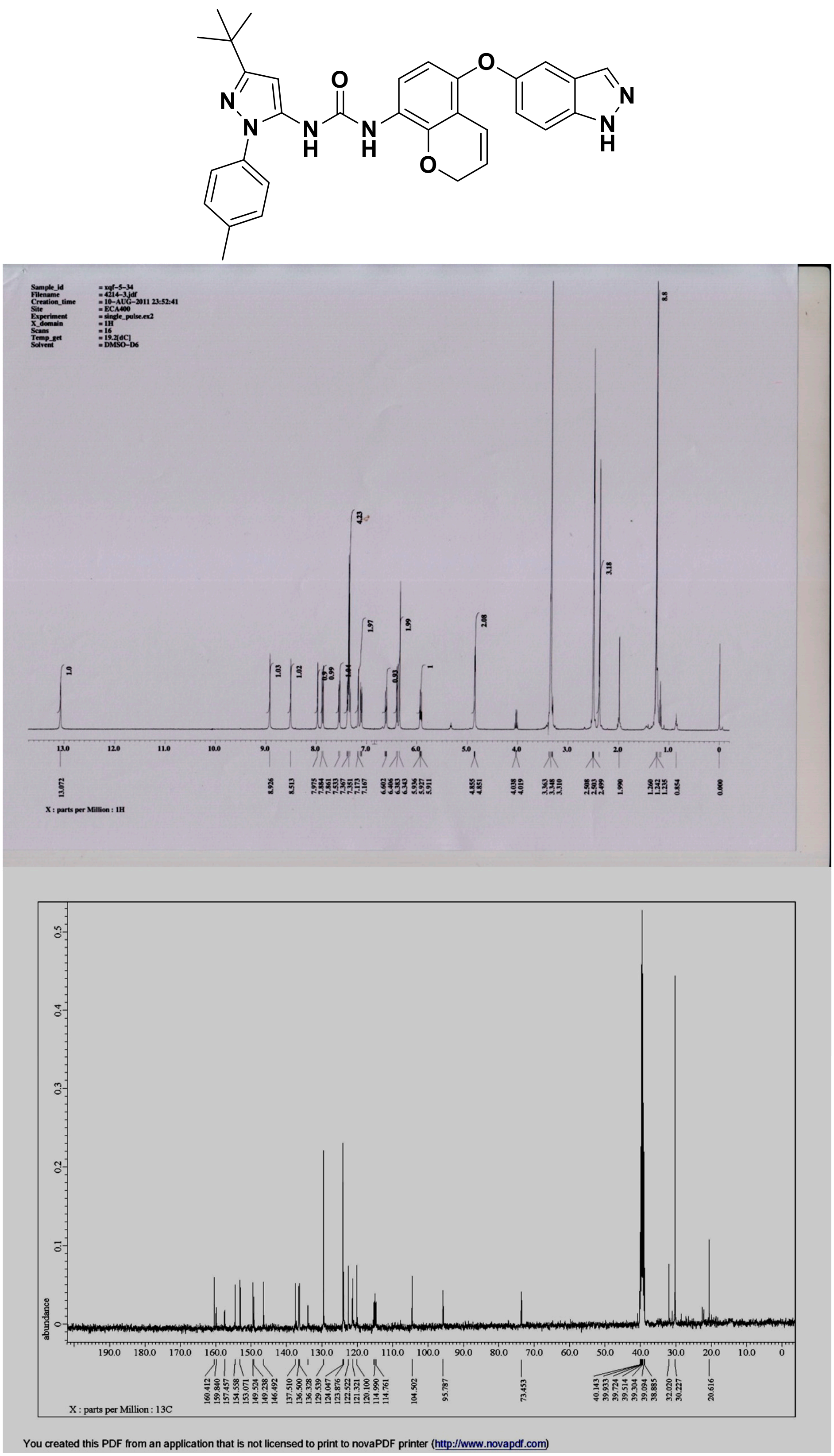


**Figure S12.** 1-(5-((1*H*-indazol-5-yl)oxy)-2*H*-chromen-8-yl)-3-(3-(tert-butyl)-1-(p-tolyl)-1*H*-pyrazol-5-yl)urea (**26a**).

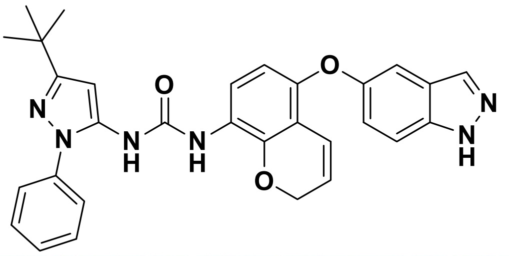


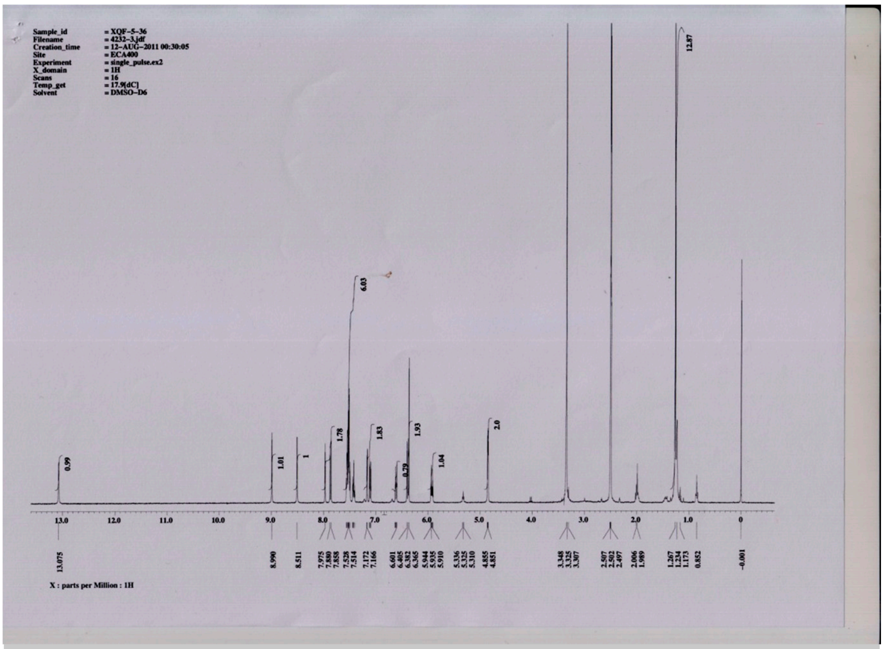


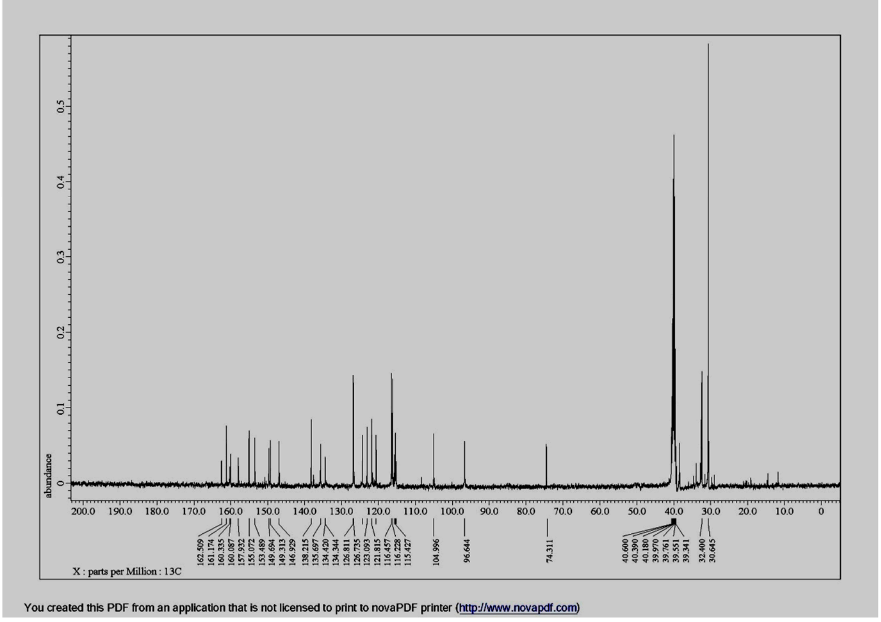


**Figure S13.** 1-(5-((1*H*-indazol-5-yl)oxy)-2*H*-chromen-8-yl)-3-(3-(tert-butyl) -1-phenyl-1*H*-pyrazol-5-yl)urea (**26b**).

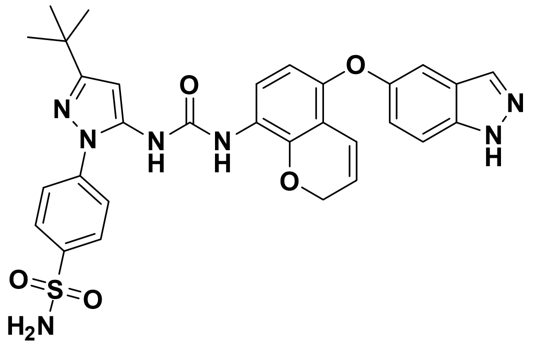

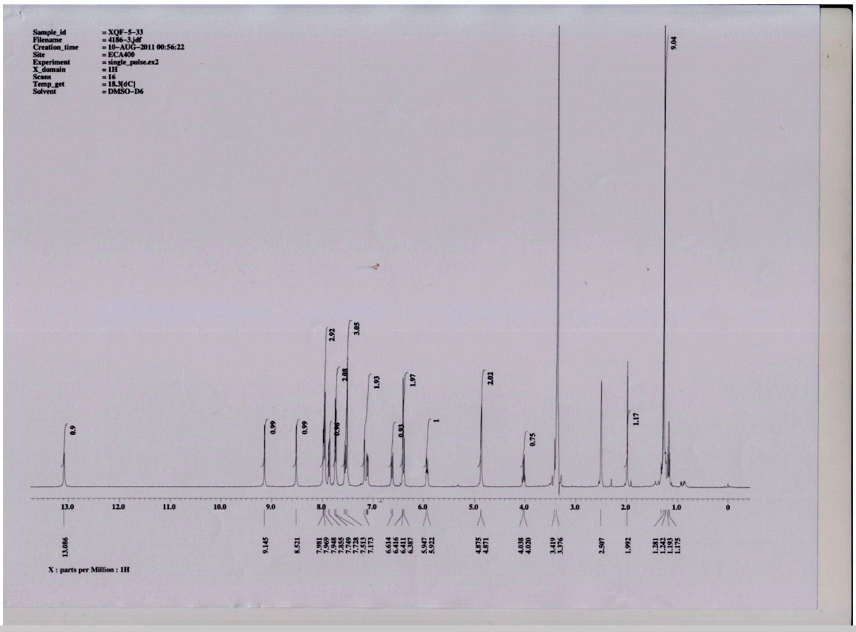

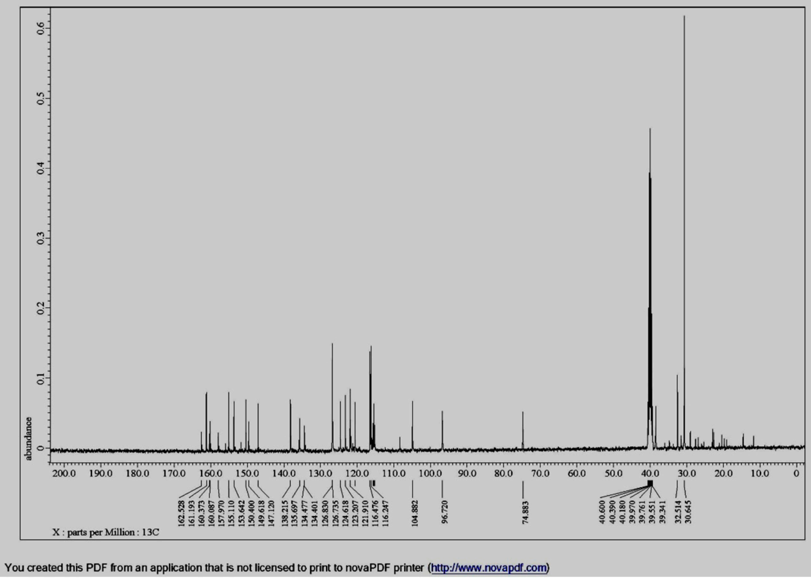


You created this PDF from an application that is not licensed to print to novaPDF printer (http://www.novapdf.com)

**Figure S14.** 4-(5-(3-(5-((1*H*-indazol-5-yl)oxy)-2*H*-chromen-8-yl)ureido)-3-(tert-butyl)-1*H*-pyrazol-1-yl)benzenesulfonamide (**26c**).

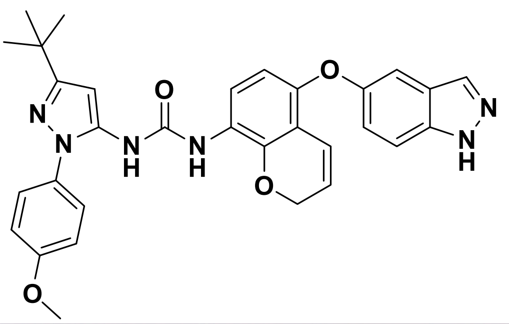

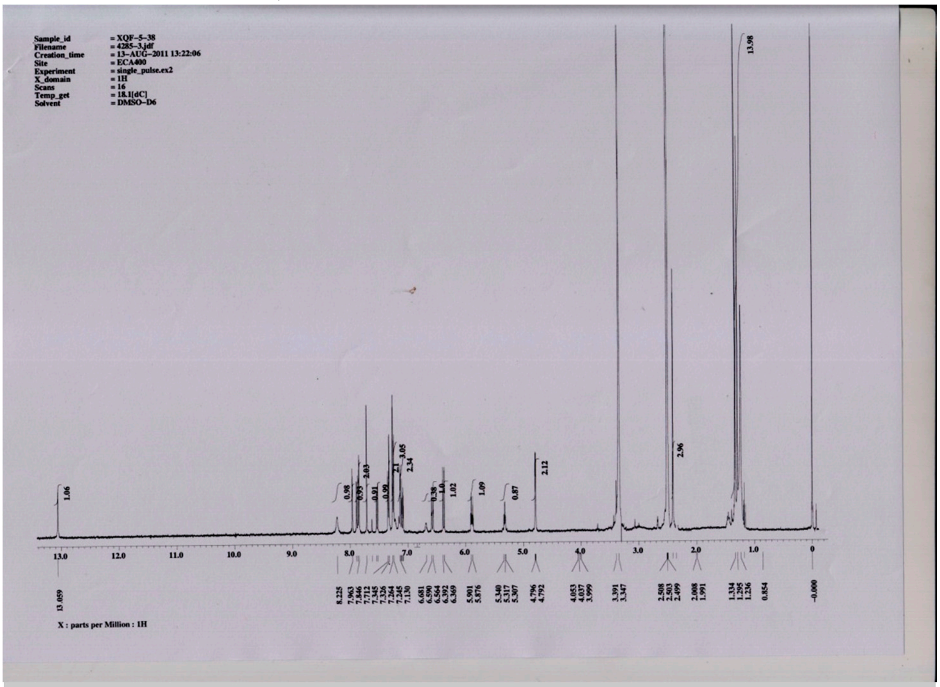


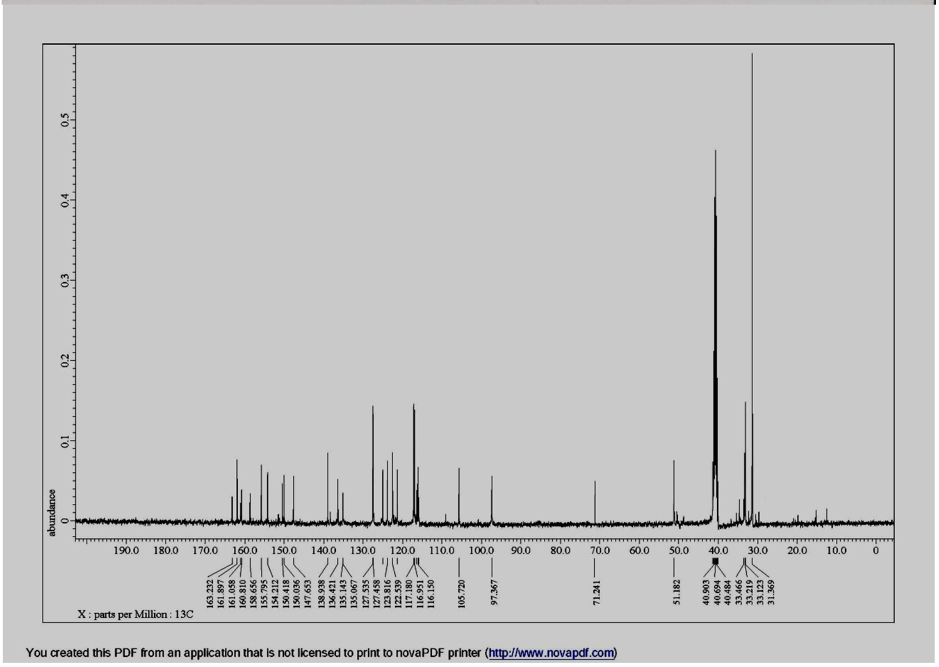


**Figure S15.** 1-(5-((1*H*-indazol-5-yl)oxy)-2*H*-chromen-8-yl)-3-(3-(tert-butyl) -1-(4-methoxyphenyl)-1*H*-pyrazol-5-yl)urea (**26d**).

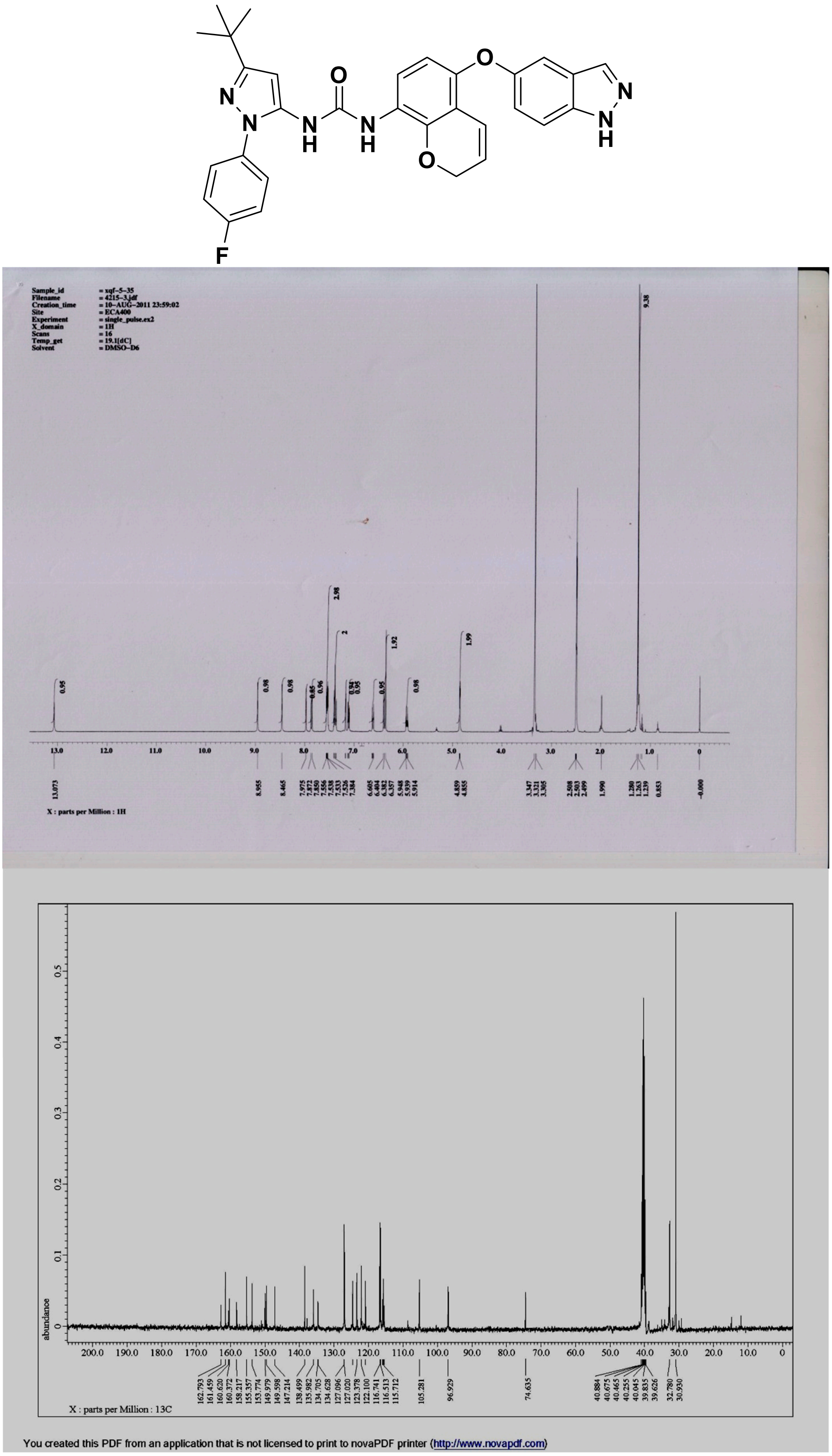


**Figure S16.** 1-(5-((1*H*-indazol-5-yl)oxy)-2*H*-chromen-8-yl)-3-(3-(tert-butyl)-1-(4-fluorophenyl)-1*H*-pyrazol-5-yl)urea (**26e**).

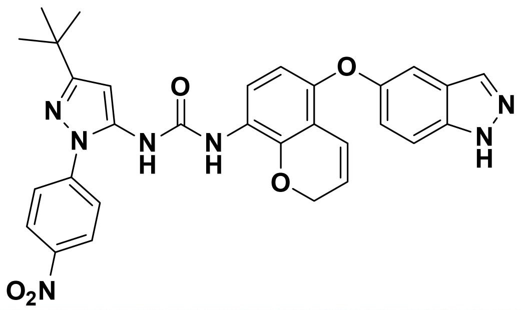

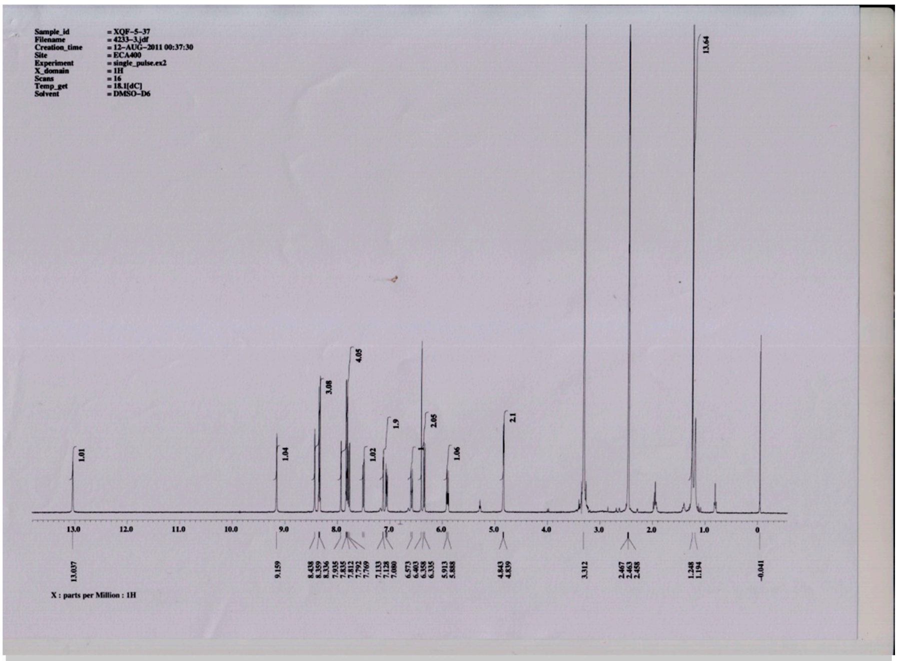


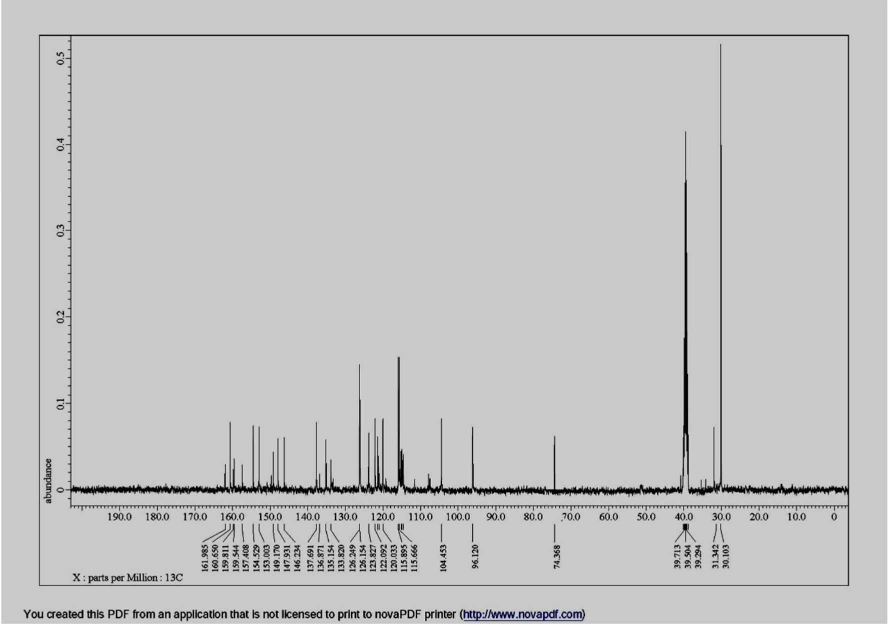


**Figure S17.** 1-(5-((1*H*-indazol-5-yl)oxy)-2*H*-chromen-8-yl)-3-(3-(tert-butyl)-1-(4-nitrophenyl)-1*H*-pyrazol-5-yl)urea (**26f**).

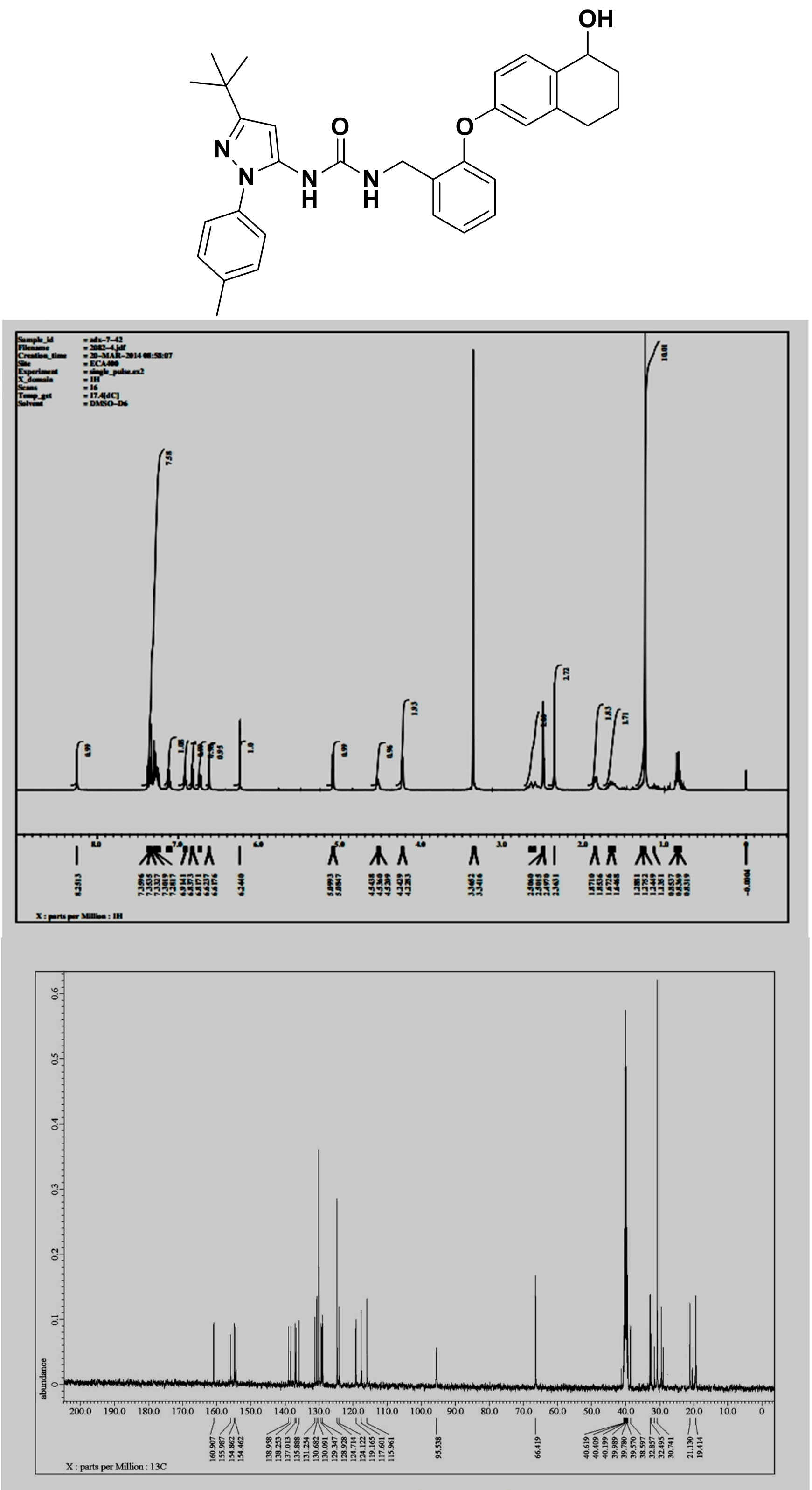


You created this PDF from an application that is not licensed to print to novaPDF printer (http://www.novapdf.com)

**Figure S18.** 1-(3-(tert-butyl)-1-(p-tolyl)-1*H*-pyrazol-5-yl)-3-(2-((5-hydroxy-5,6,7,8-tetrahydronaphthalen-2-yl)oxy)benzyl)urea (**27a**).

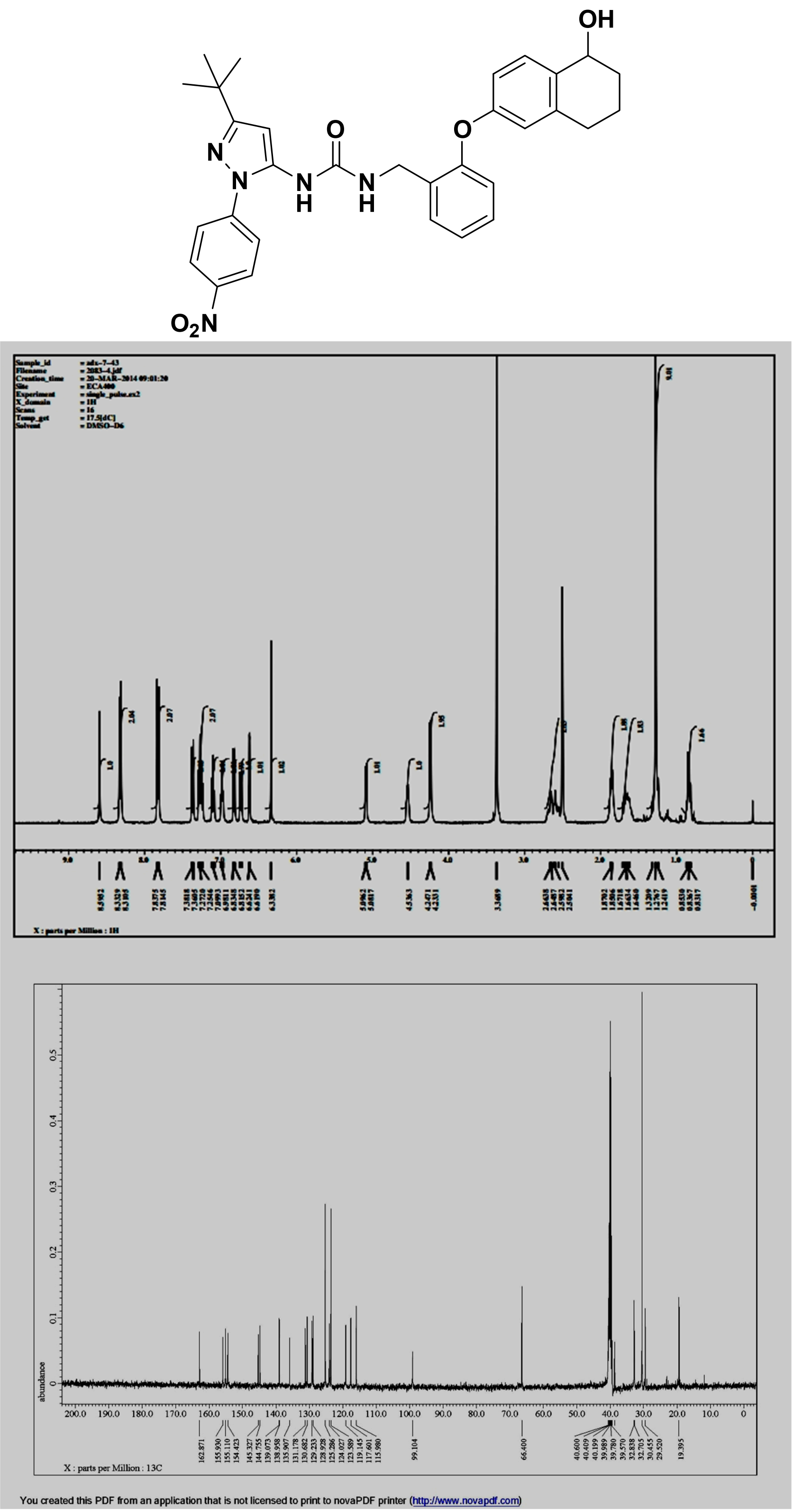


**Figure S19.** 1-(3-(tert-butyl)-1-(4-nitrophenyl)-1*H*-pyrazol-5-yl)-3-(2-((5-hydroxy-5,6,7,8-tetrahydronaphthalen-2-yl)oxy)benzyl)urea (**27b**).

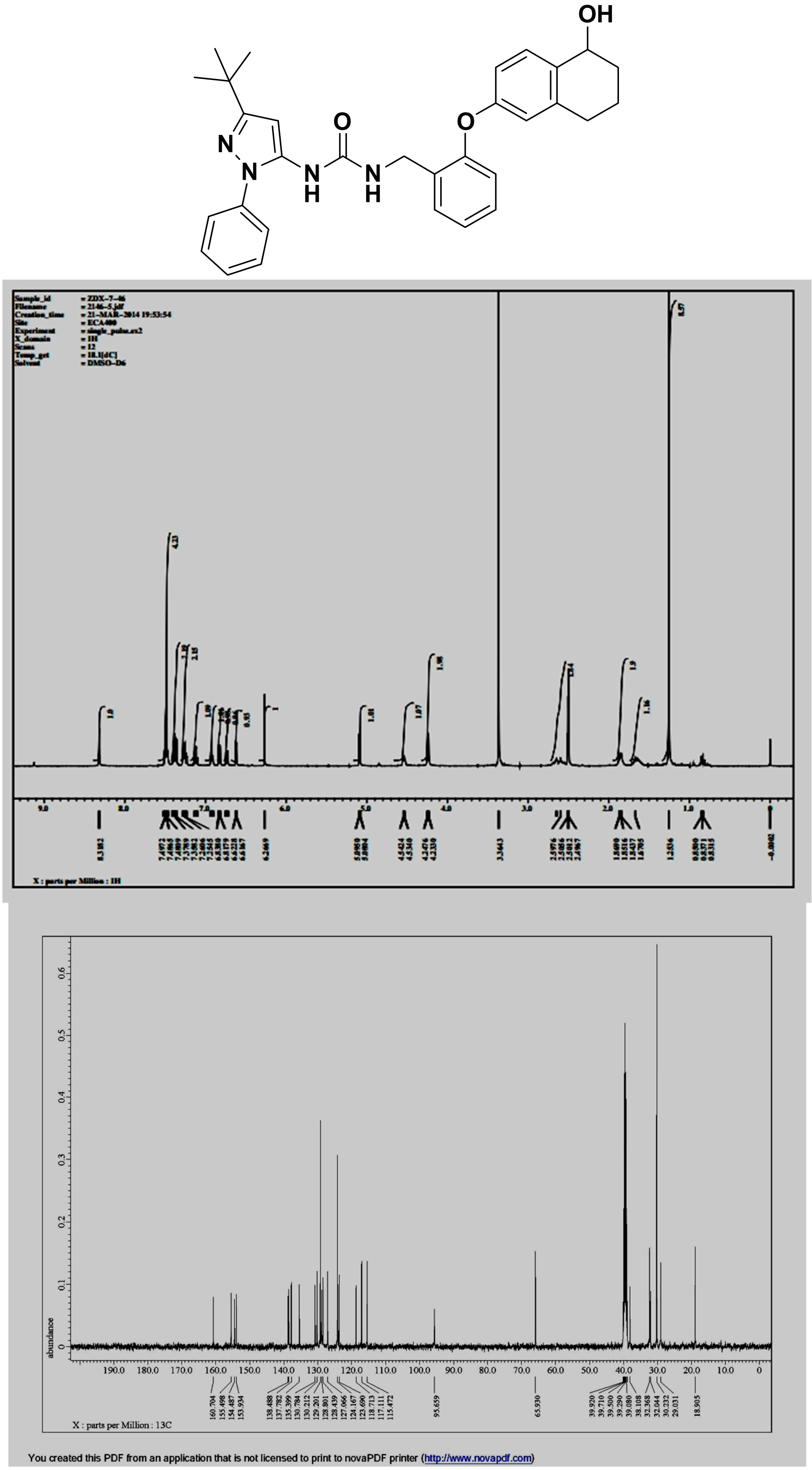


**Figure S20.** 1-(3-(tert-butyl)-1-phenyl-1*H*-pyrazol-5-yl)-3-(2-((5-hydroxy-5,6,7,8-tetrahydronaphthalen-2-yl)oxy)benzyl)urea (**27c**).

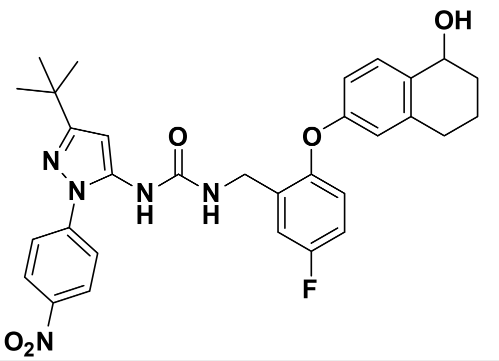


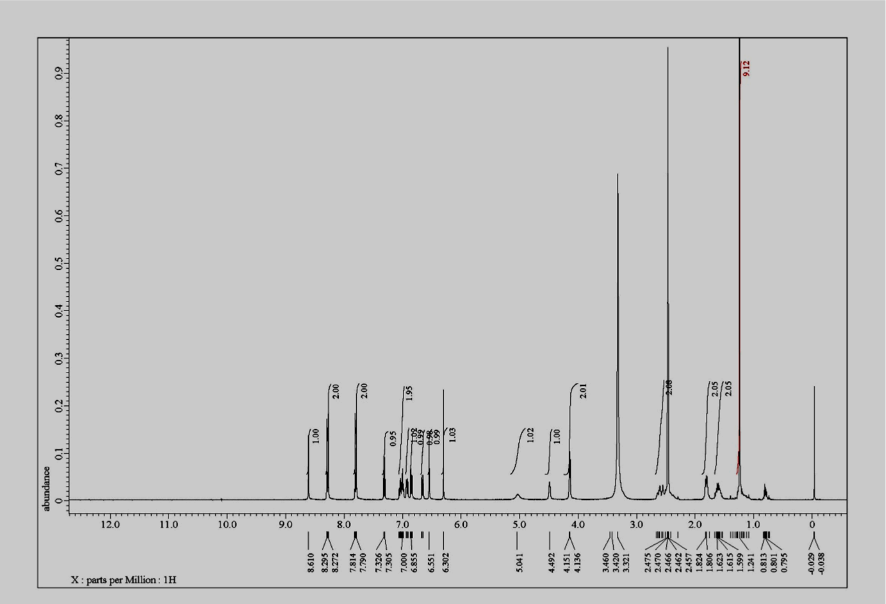


You created this PDF from an application that is not licensed to print to novaPDF printer (http://www.novapdf.com)

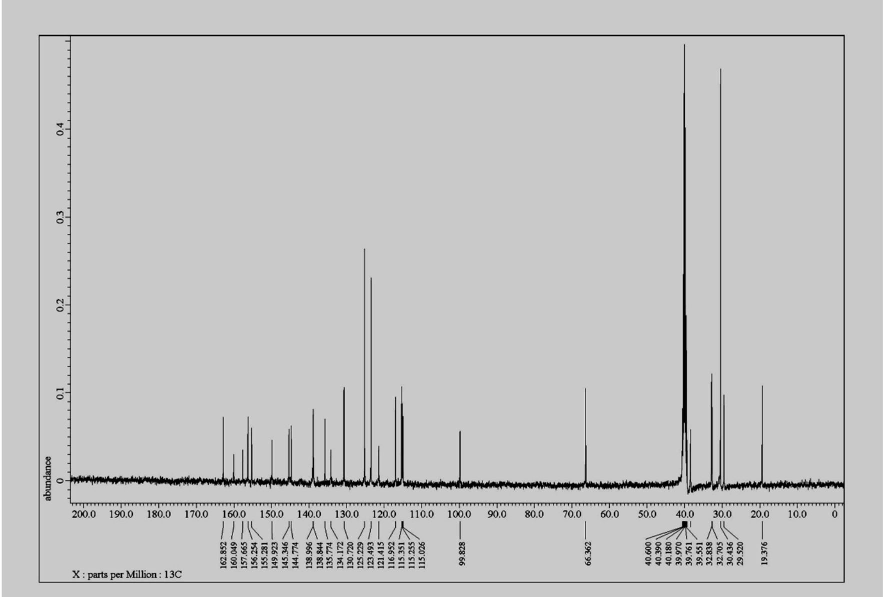


You created this PDF from an application that is not licensed to print to novaPDF printer (http://www.novapdf.com)

**Figure S21.** 1-(3-(tert-butyl)-1-(4-nitrophenyl)-1*H*-pyrazol-5-yl)-3-(5-fluoro-2-((5-hydroxy-5,6,7,8-tetrahydronaphthalen-2-yl)oxy)benzyl)urea (**27d**).

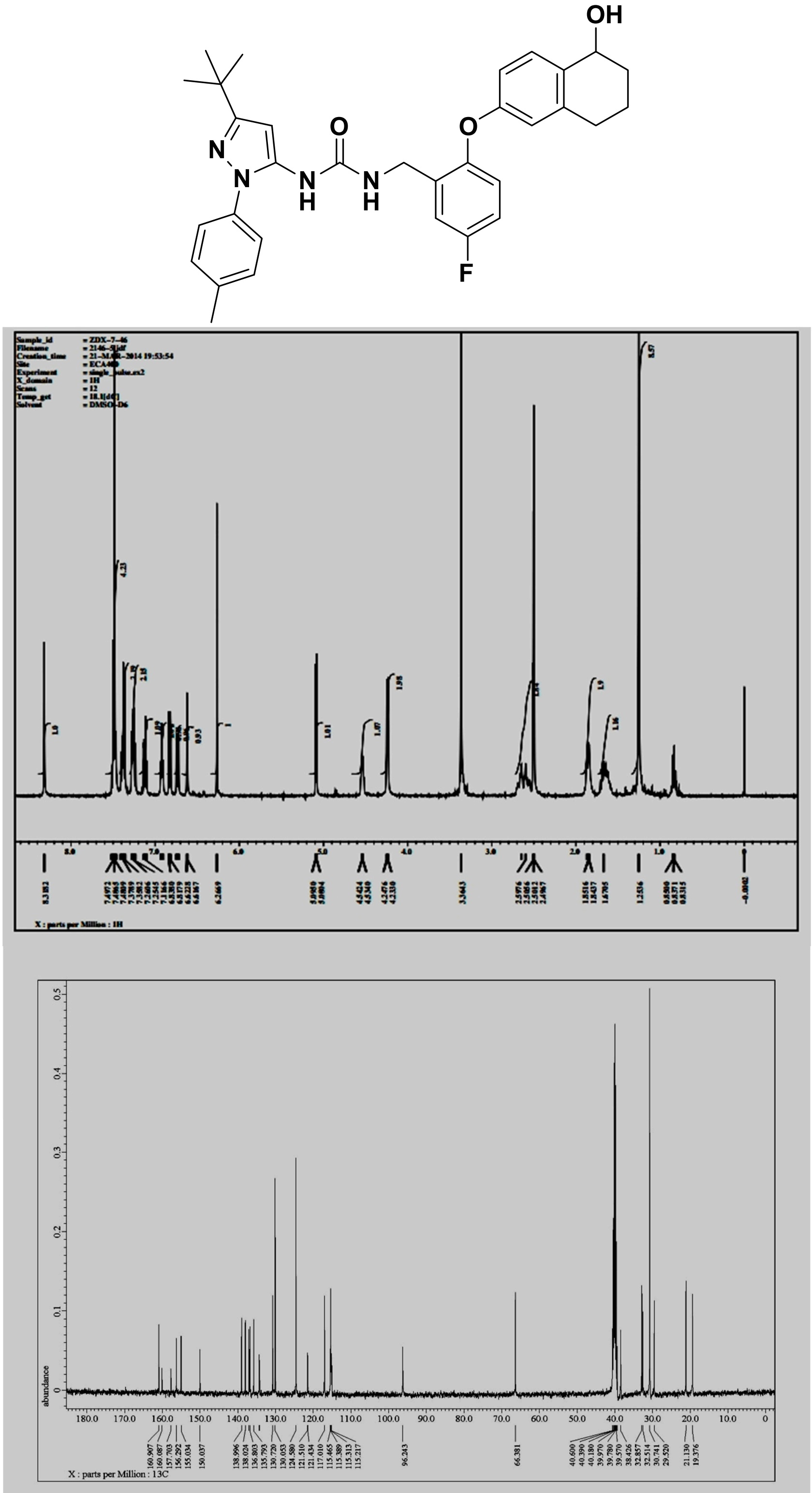


You created this PDF from an application that is not licensed to print to novaPDF printer (http://www.novapdf.com)

**Figure S22.** 1-(3-(tert-butyl)-1-(p-tolyl)-1*H*-pyrazol-5-yl)-3-(5-fluoro-2-((5-hydroxy-5,6,7,8-tetrahydronaphthalen-2-yl)oxy)benzyl)urea (**27e**).

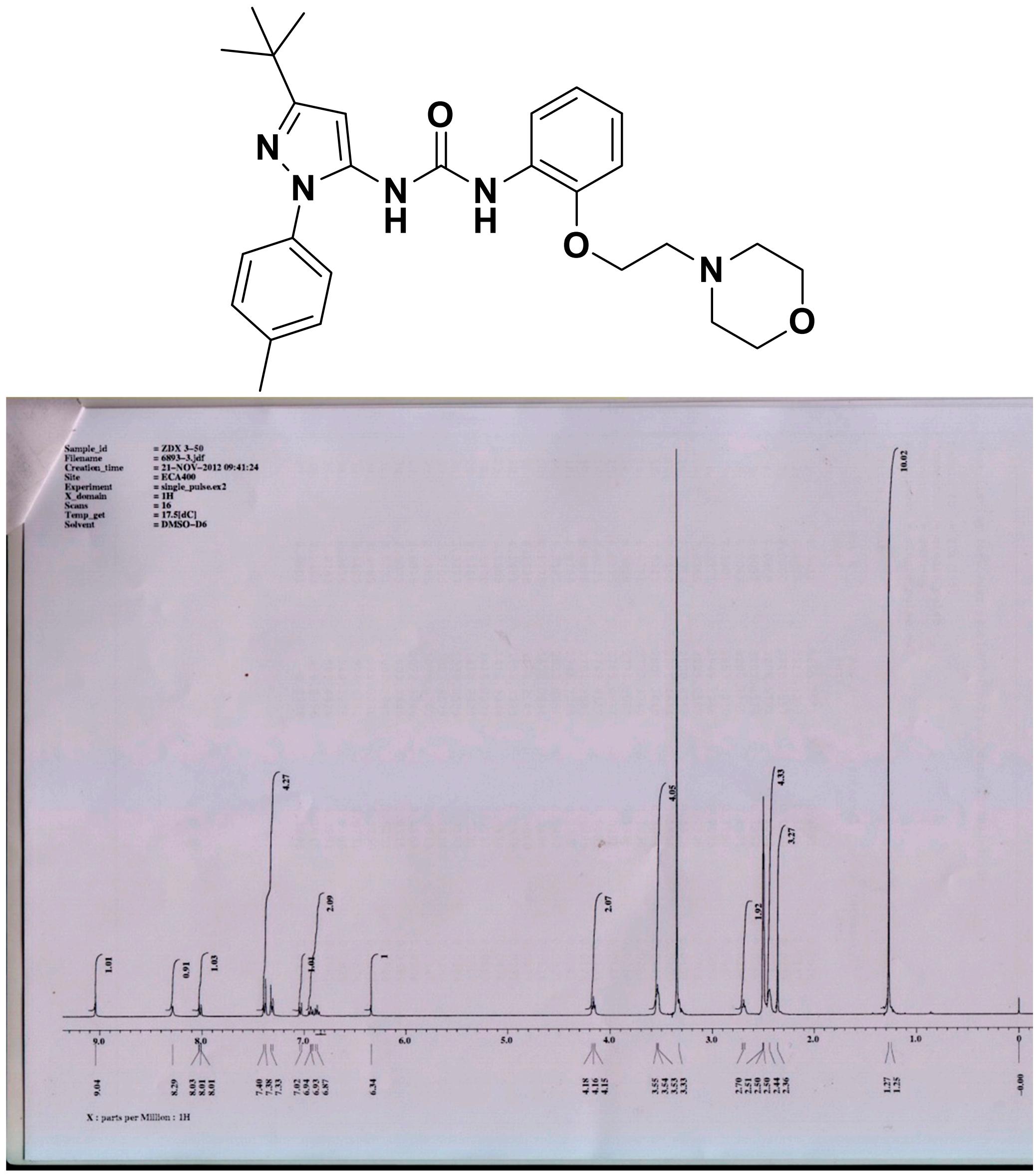


**Figure S23.** 1-(3-(tert-butyl)-1-(p-tolyl)-1*H*-pyrazol-5-yl)-3-(2-(2-morpholinoethoxy)phenyl)urea (**28a**).

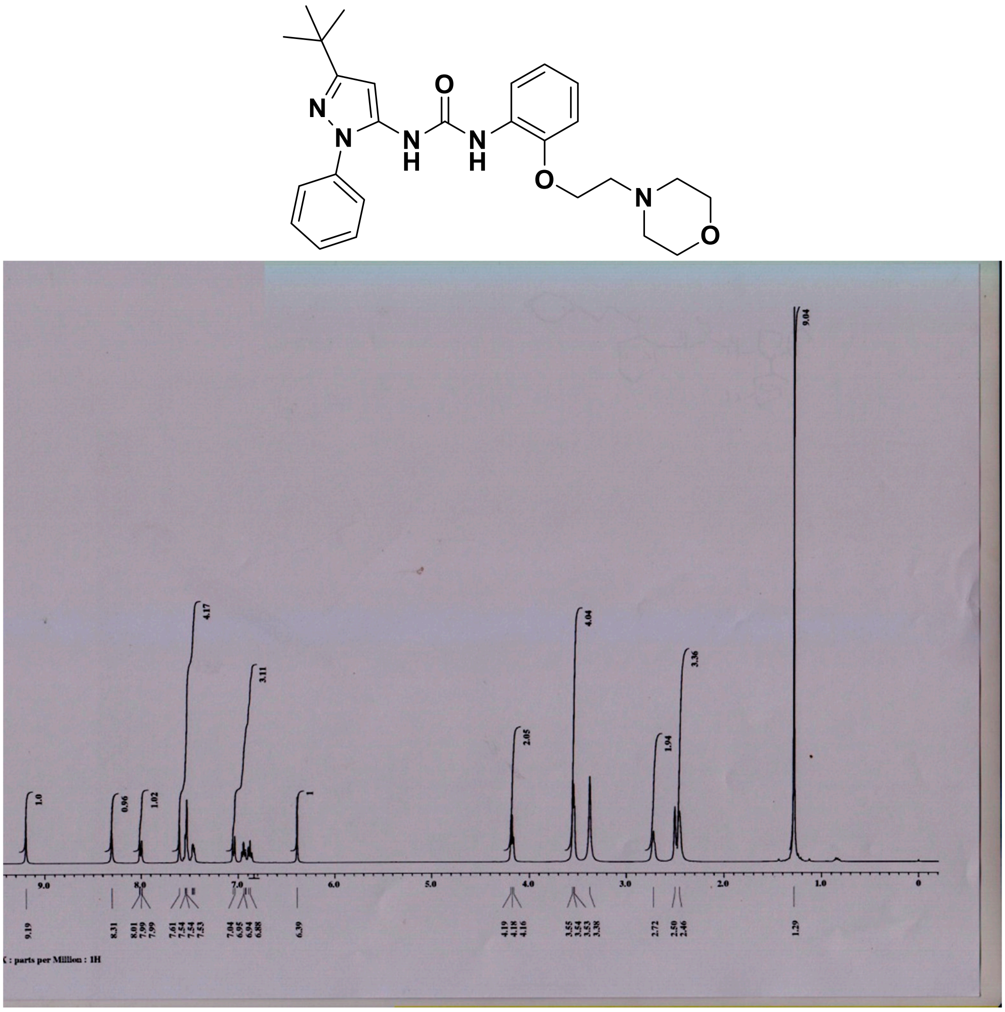


**Figure S24.** 1-(3-(tert-butyl)-1-phenyl-1*H*-pyrazol-5-yl)-3-(2-(2-morpholinoethoxy)phenyl)urea (**28b**).

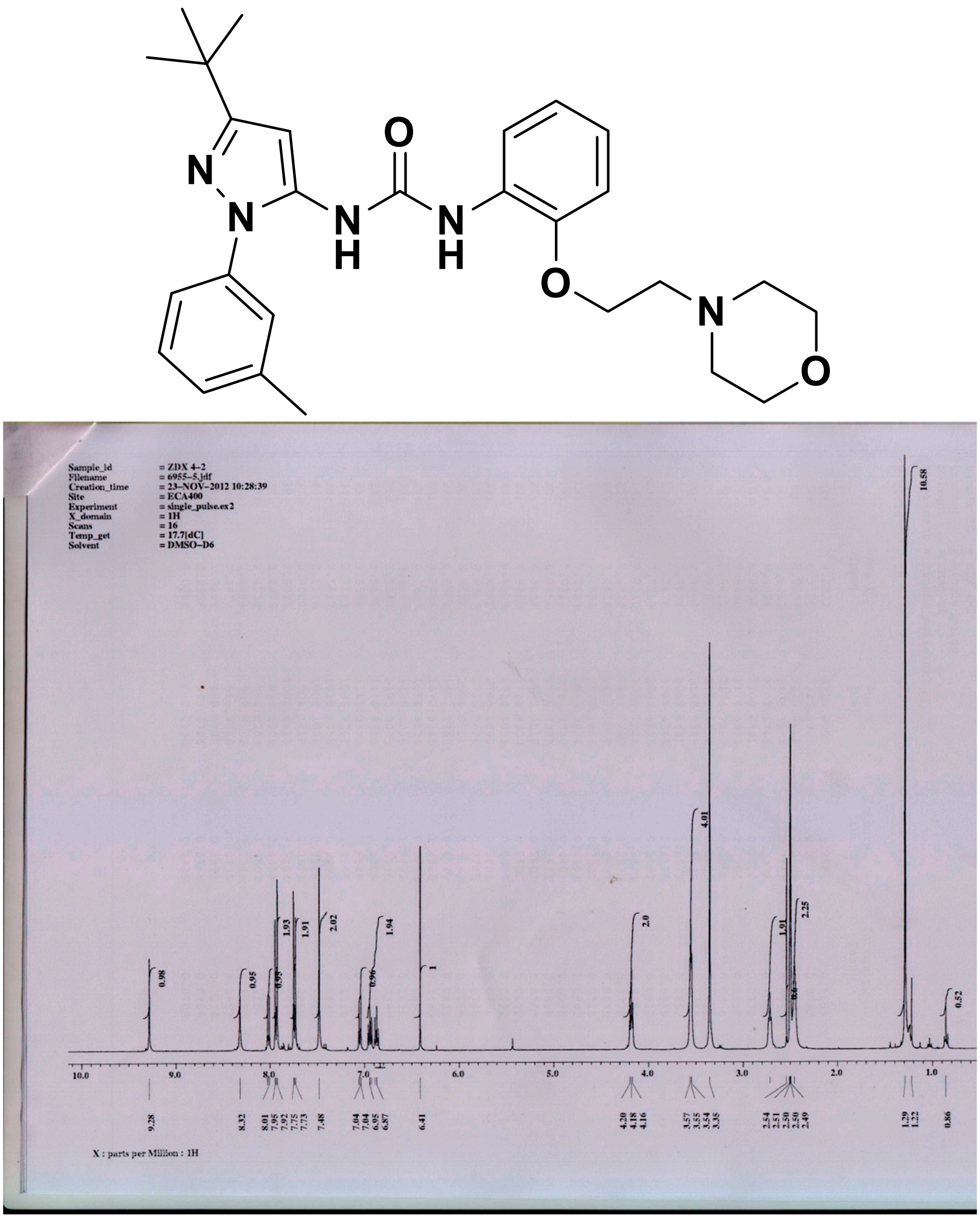


**Figure S25.** 1-(3-(tert-butyl)-1-(m-tolyl)-1*H*-pyrazol-5-yl)-3-(2-(2-morpholinoethoxy)phenyl)urea (**28c**).

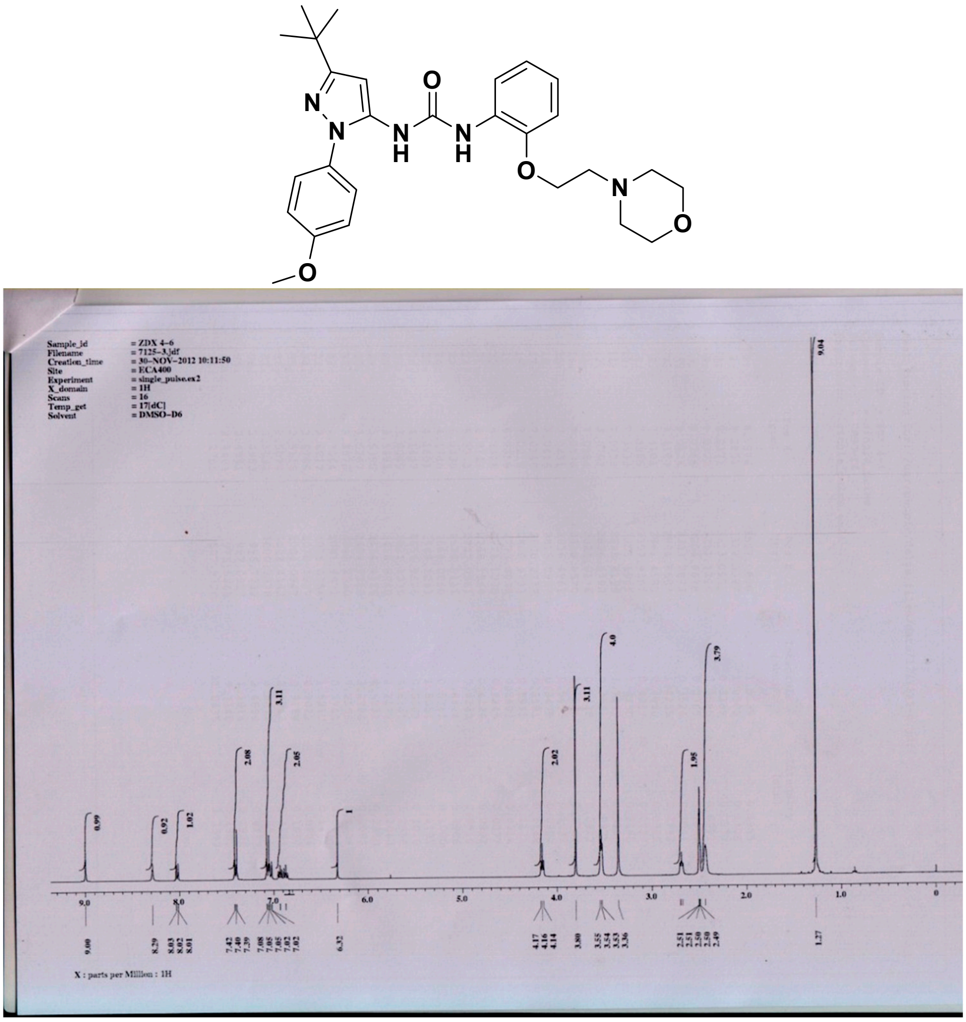

**Figure S26.** 1-(3-(tert-butyl)-1-(4-methoxyphenyl)-1*H*-pyrazol-5-yl)-3-(2-(2-morpholinoethoxy)phenyl)urea (**28d**).

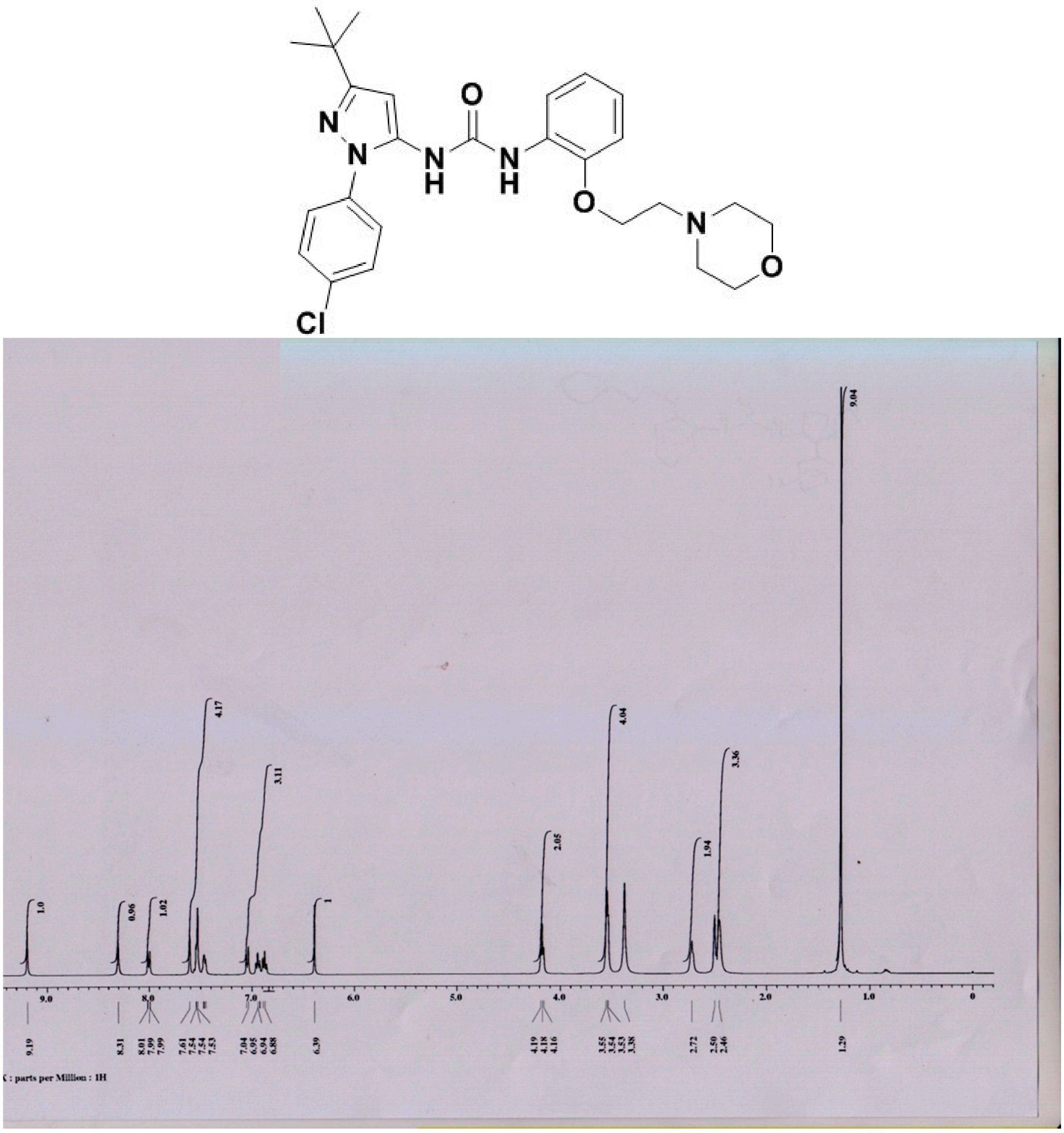

**Figure S27.** 1-(3-(tert-butyl)-1-(4-chlorophenyl)-1*H*-pyrazol-5-yl)-3-(2-(2-morpholinoethoxy)phenyl)urea (**28e**).

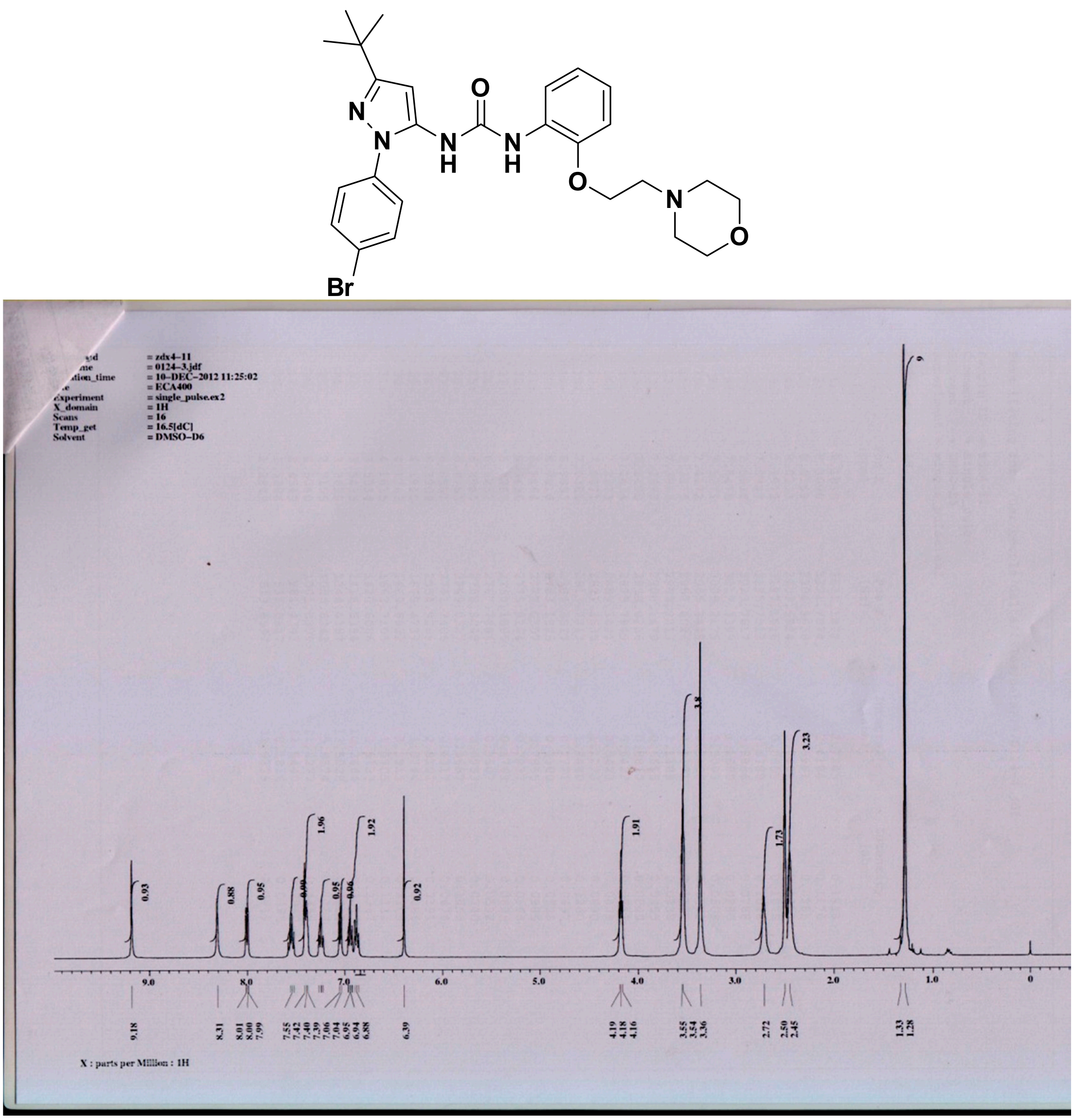


**Figure S28.** 1-(1-(4-bromophenyl)-3-(tert-butyl)-1H-pyrazol-5-yl)-3-(2-(2-morpholinoethoxy)phenyl)urea (**28f**).

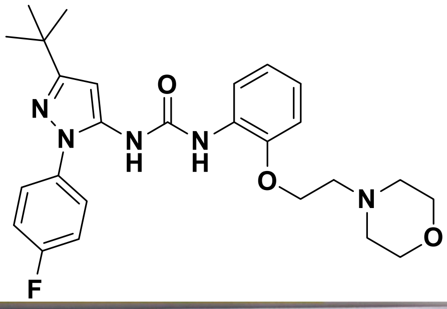

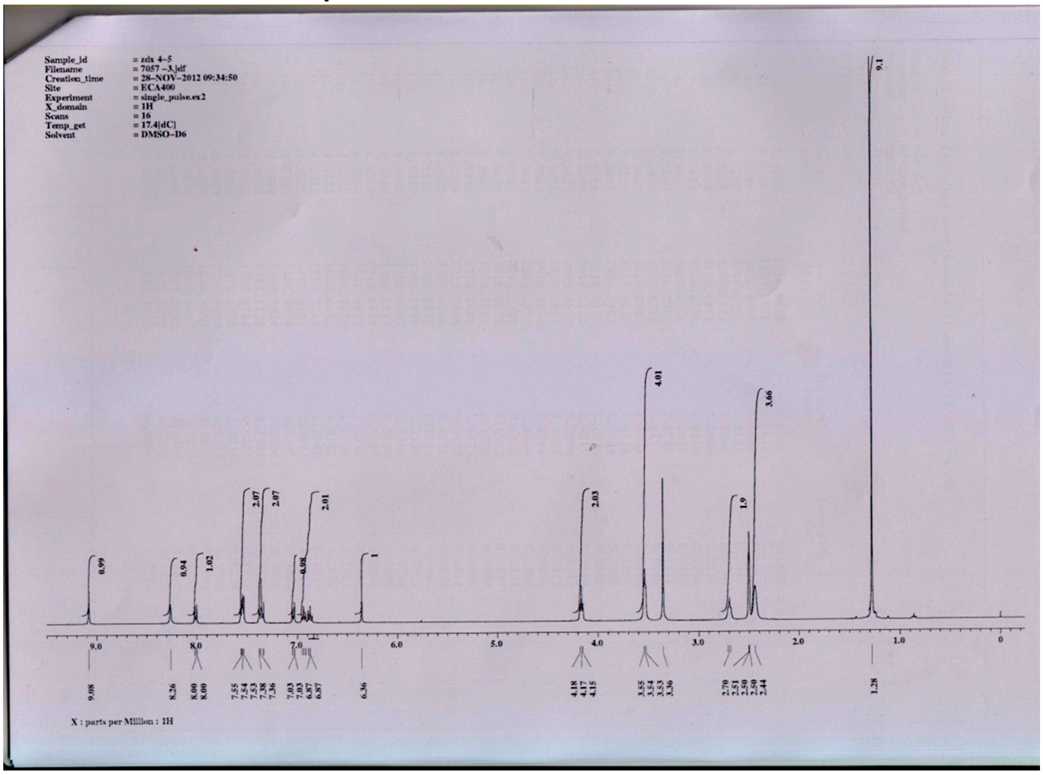

**Figure S29.** 1-(3-(tert-butyl)-1-(4-fluorophenyl)-1H-pyrazol-5-yl)-3-(2-(2-morpholinoethoxy)phenyl)urea (**28g**).

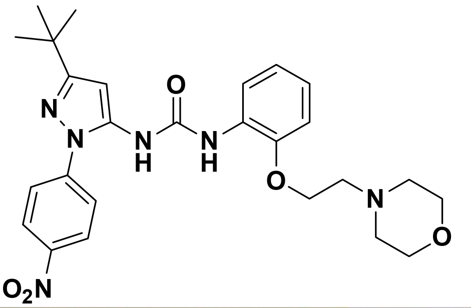

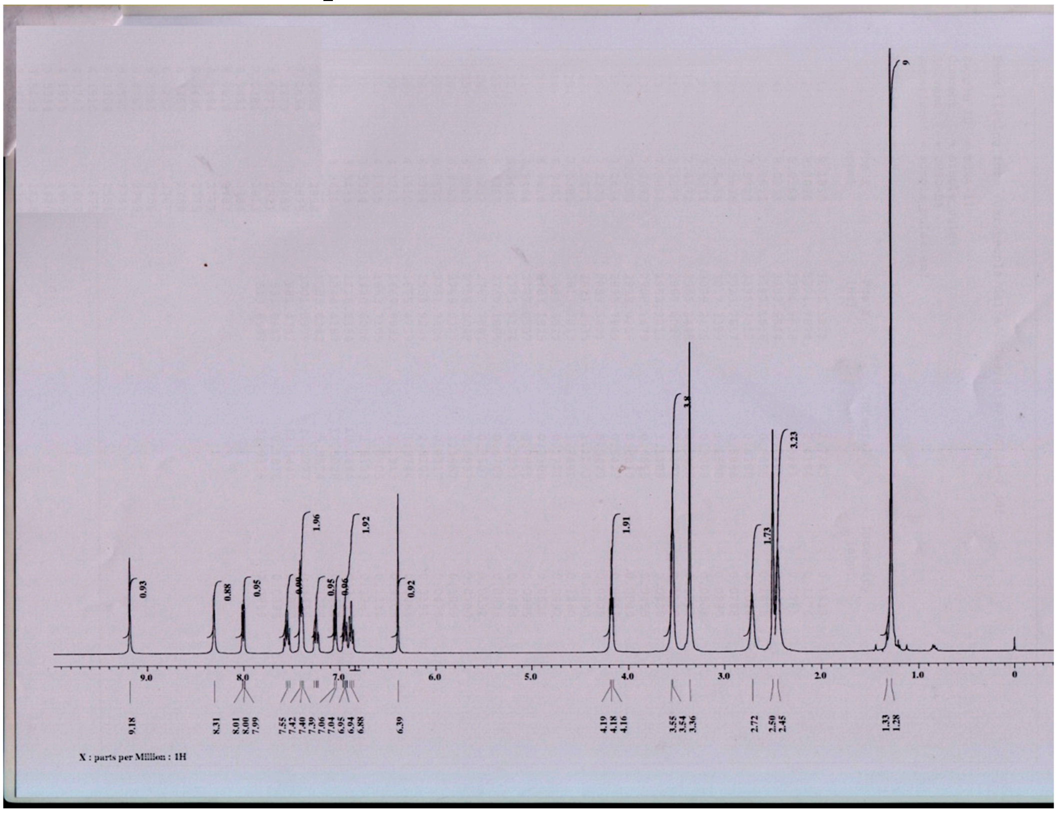

**Figure S30.** 1-(3-(tert-butyl)-1-(4-nitrophenyl)-1H-pyrazol-5-yl)-3-(2-(2-morpholinoethoxy)phenyl)urea (**28h**).

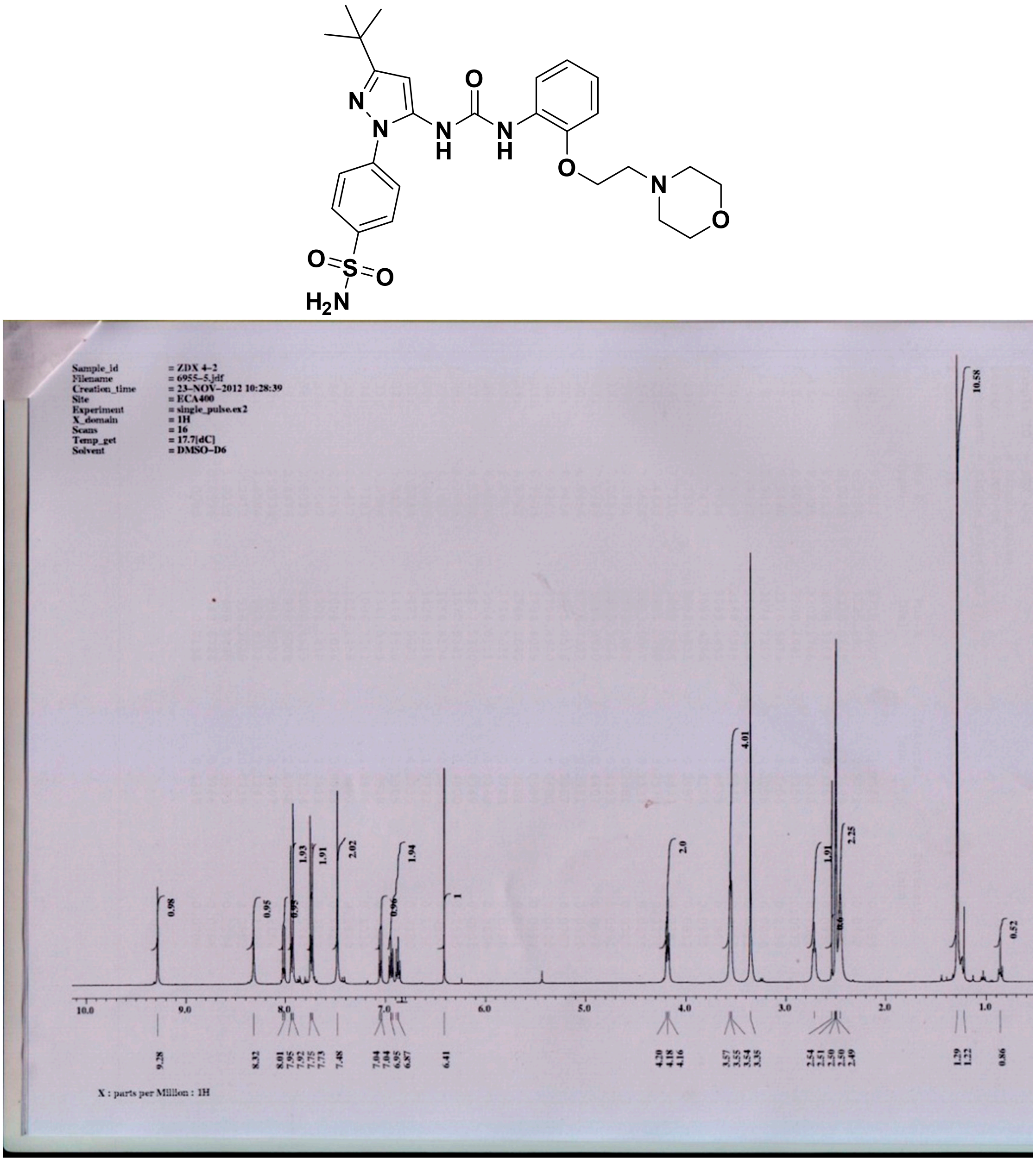


**Figure S31.** 4-(3-(tert-butyl)-5-(3-(2-(2-morpholinoethoxy)phenyl)ureido)-1H -pyrazol-1-yl)benzenesulfonamide (**28i**).

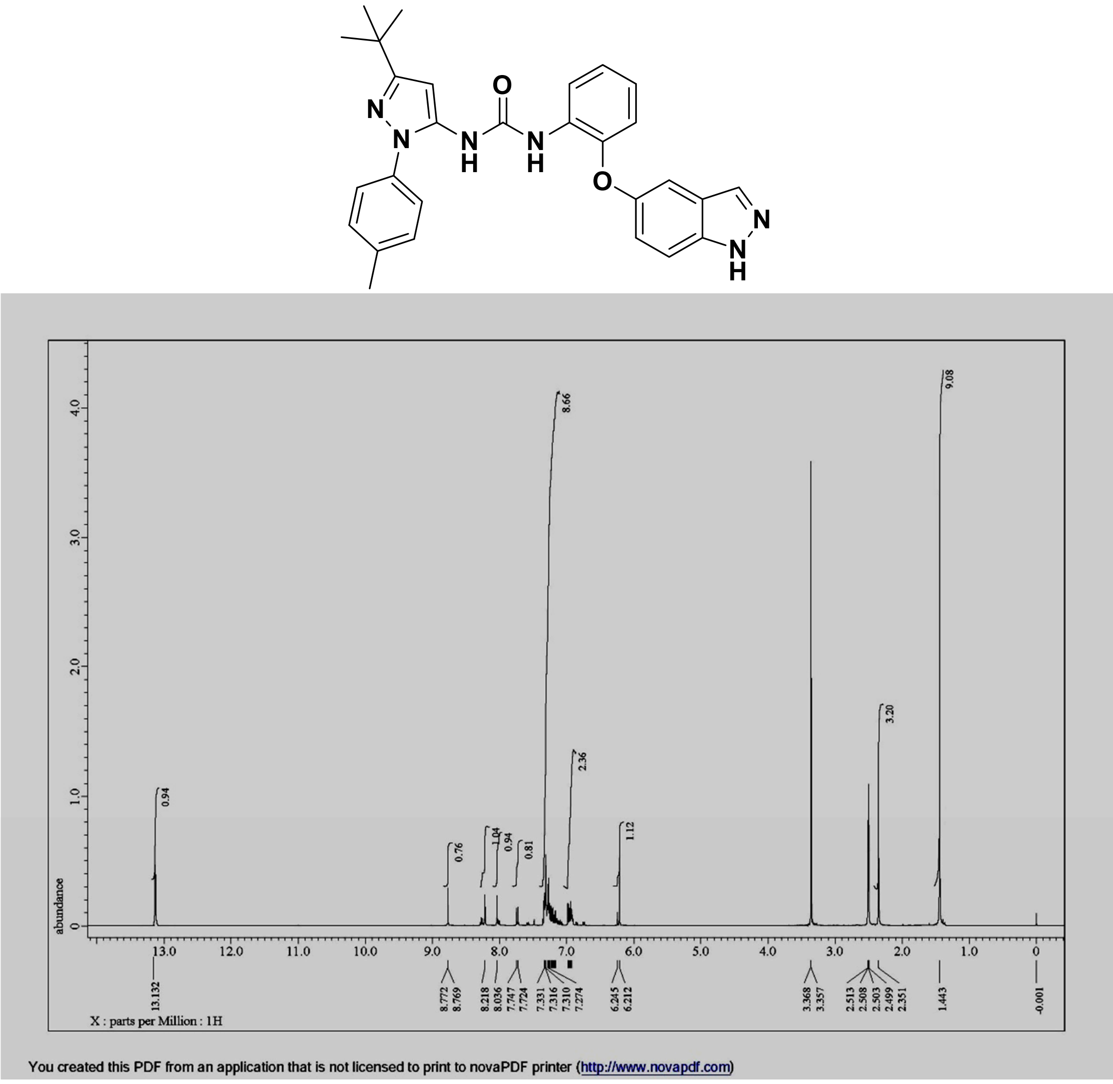


**Figure S32.** 1-(2-((1H-indazol-5-yl)oxy)phenyl)-3-(3-(tert-butyl)-1-(p-tolyl)-1H-pyrazol-5-yl)urea(**29a**).

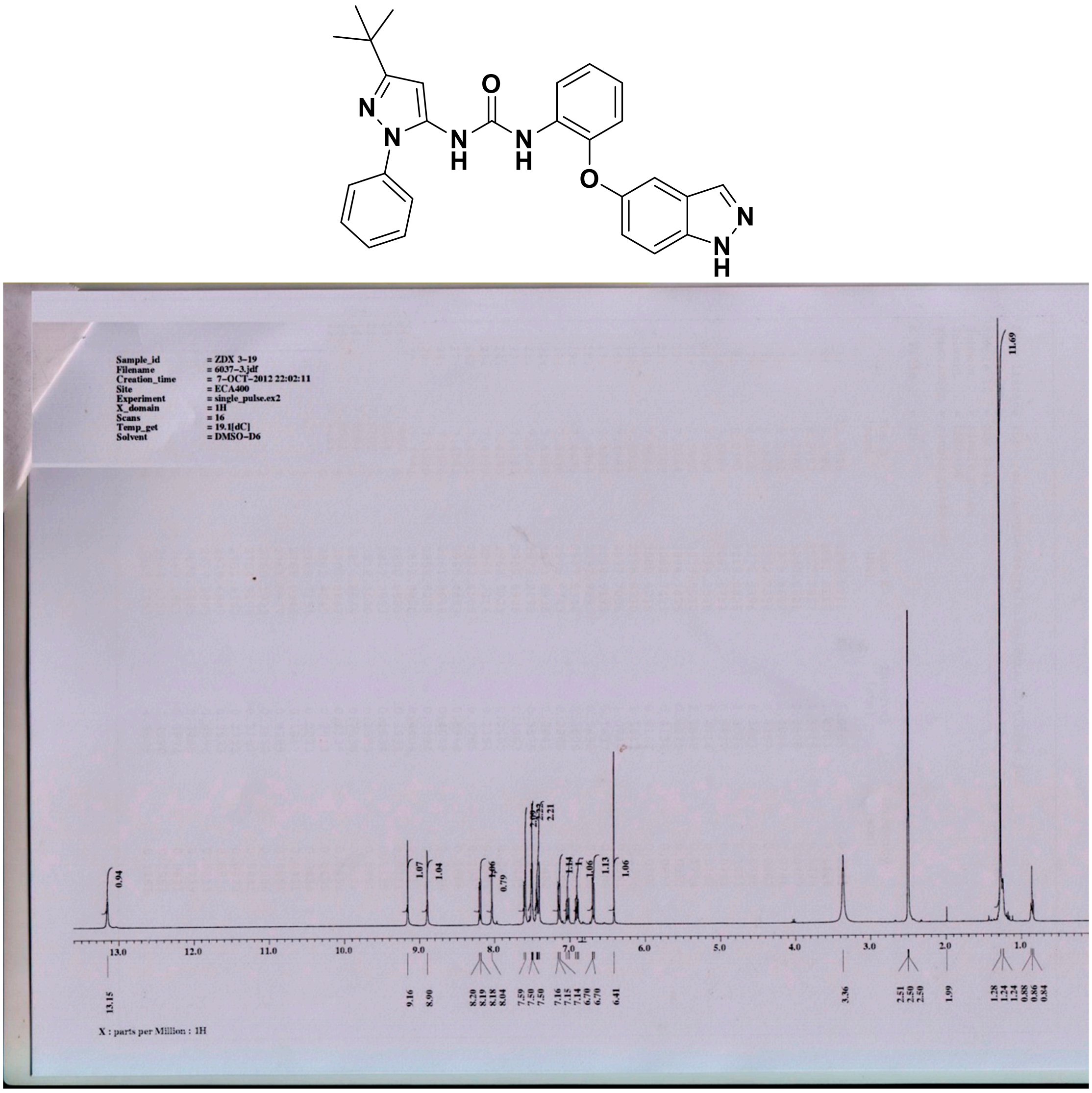


**Figure S33.** 1-(2-((1H-indazol-5-yl)oxy)phenyl)-3-(3-(tert-butyl)-1-phenyl-1H-pyrazol-5-yl)urea(**29b**).

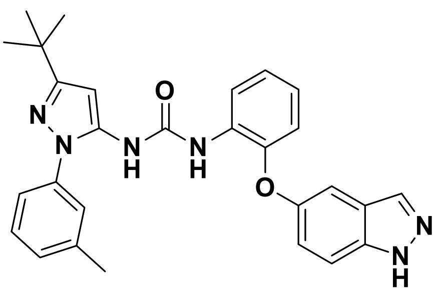


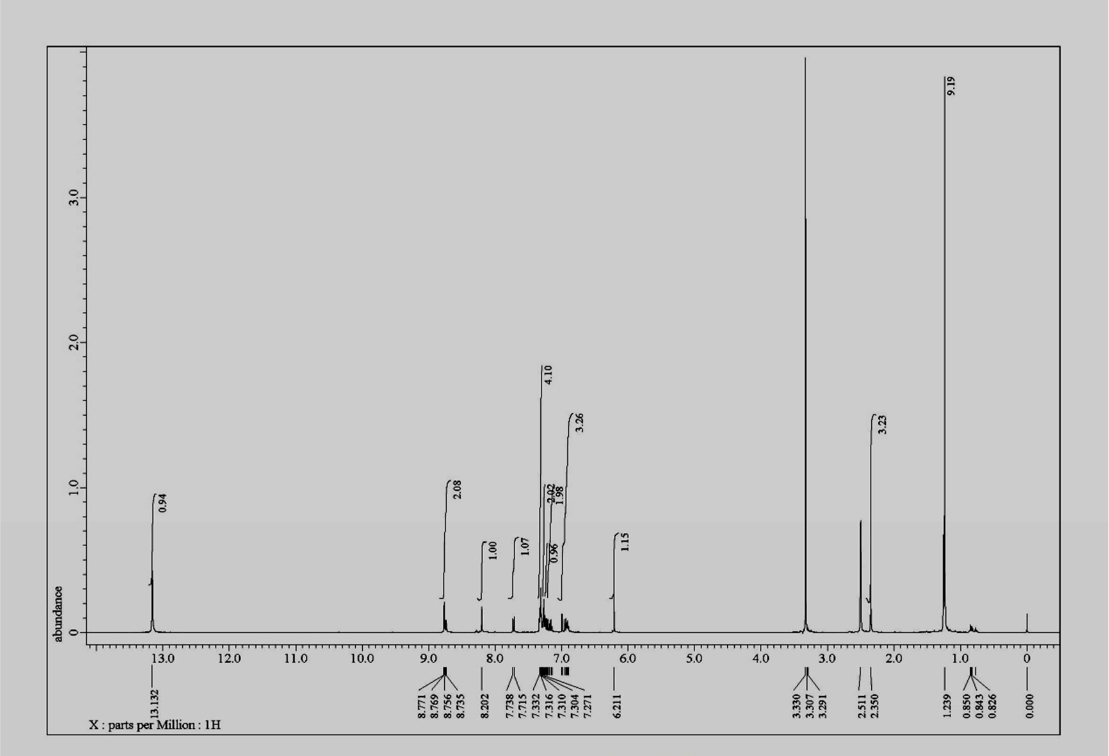


You created this PDF from an application that is not licensed to print to novaPDF printer (http://www.novapdf.com)

**Figure S34.** 1-(2-((1H-indazol-5-yl)oxy)phenyl)-3-(3-(tert-butyl)-1-(m-tolyl)-1H-pyrazol-5-yl)urea(**29c**).

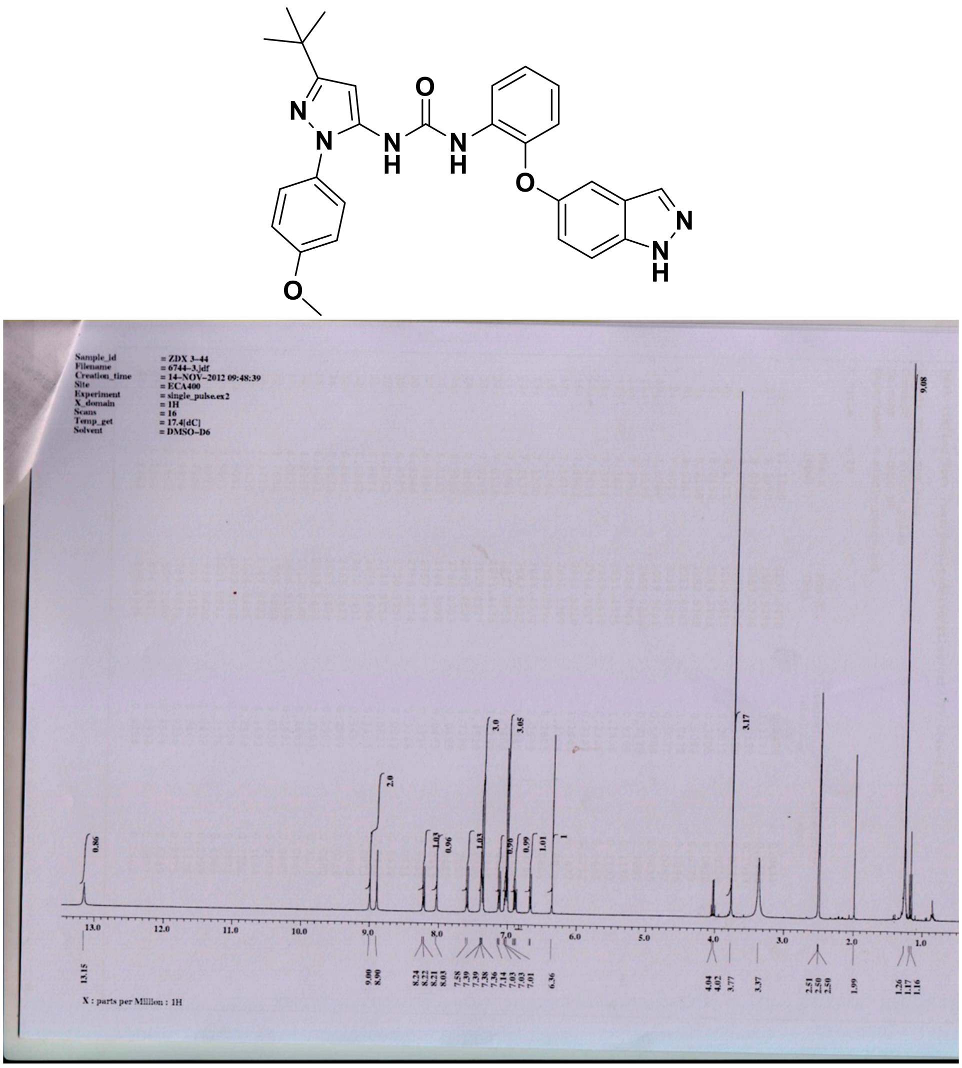

**Figure S35.** 1-(2-((1H-indazol-5-yl)oxy)phenyl)-3-(3-(tert-butyl)-1-(4-methoxyphenyl)-1H-pyrazol-5-yl)urea (**29d**).

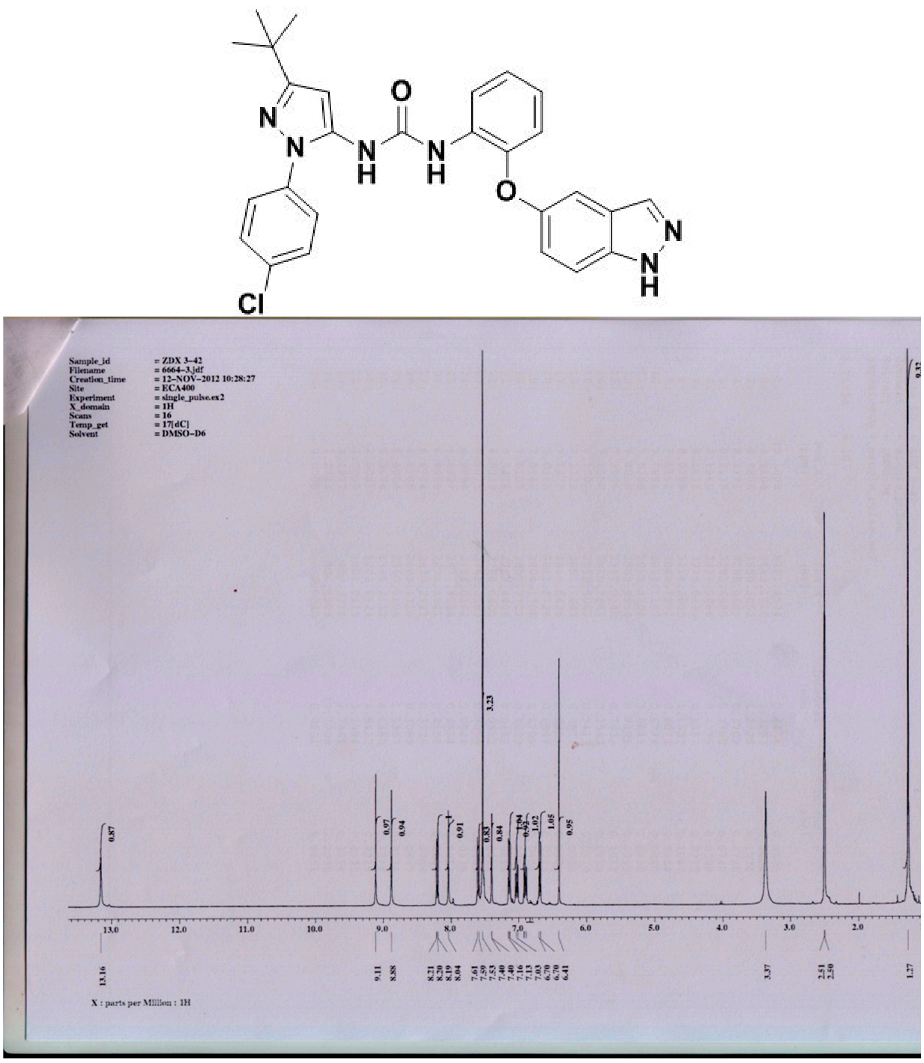


**Figure S36.** 1-(2-((1H-indazol-5-yl)oxy)phenyl)-3-(3-(tert-butyl)-1-(4-chlorophenyl)-1H-pyrazol-5-yl)urea (**29e**).

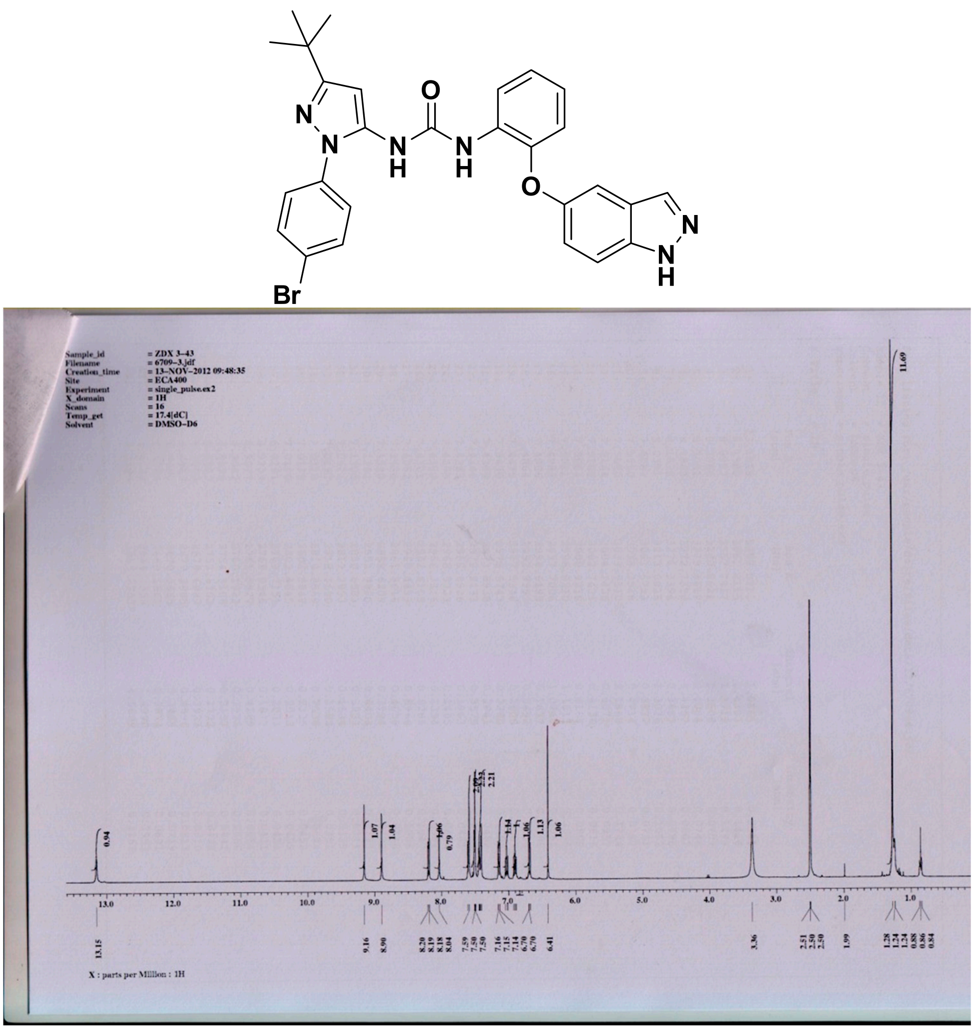

**Figure S37.** 1-(2-((1H-indazol-5-yl)oxy)phenyl)-3-(1-(4-bromophenyl)-3-(tert-butyl)-1H-pyrazol-5-yl)urea (**29f**).

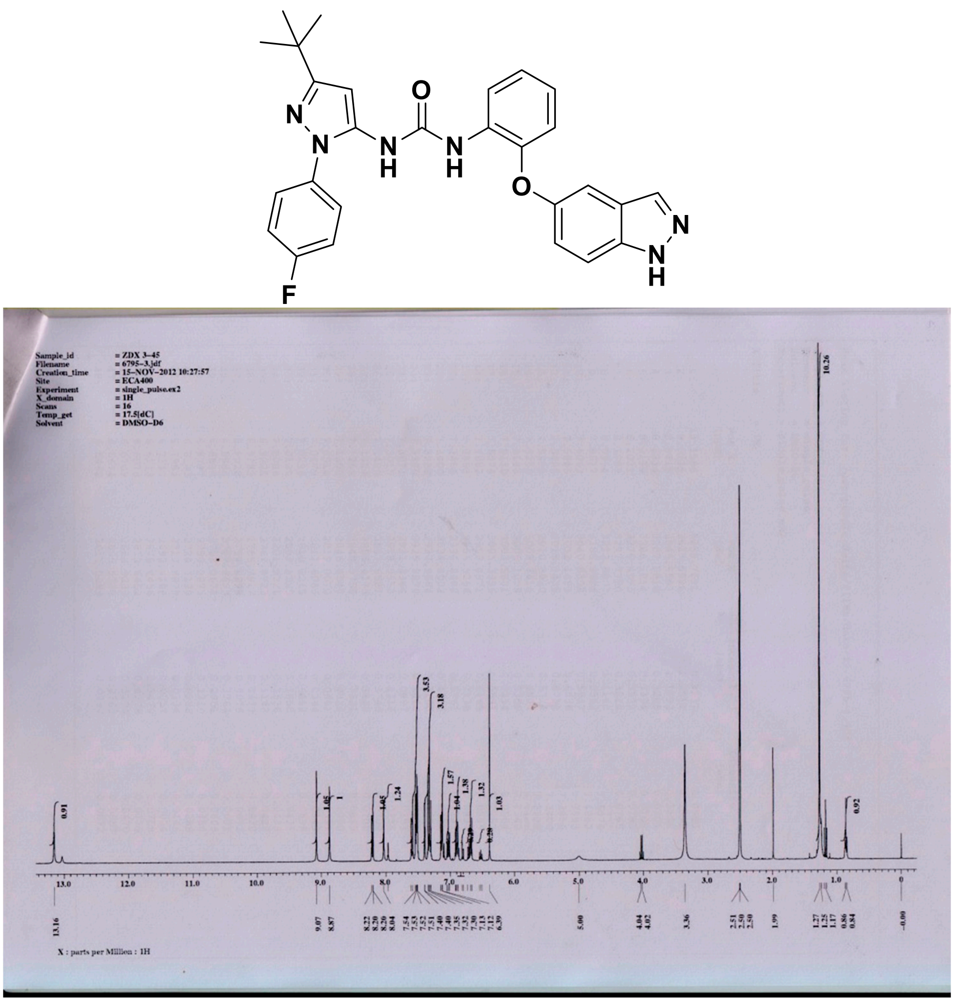

**Figure S38.** 1-(2-((1H-indazol-5-yl)oxy)phenyl)-3-(3-(tert-butyl)-1-(4-fluorophenyl)-1H-pyrazol-5-yl)urea (**29g**).

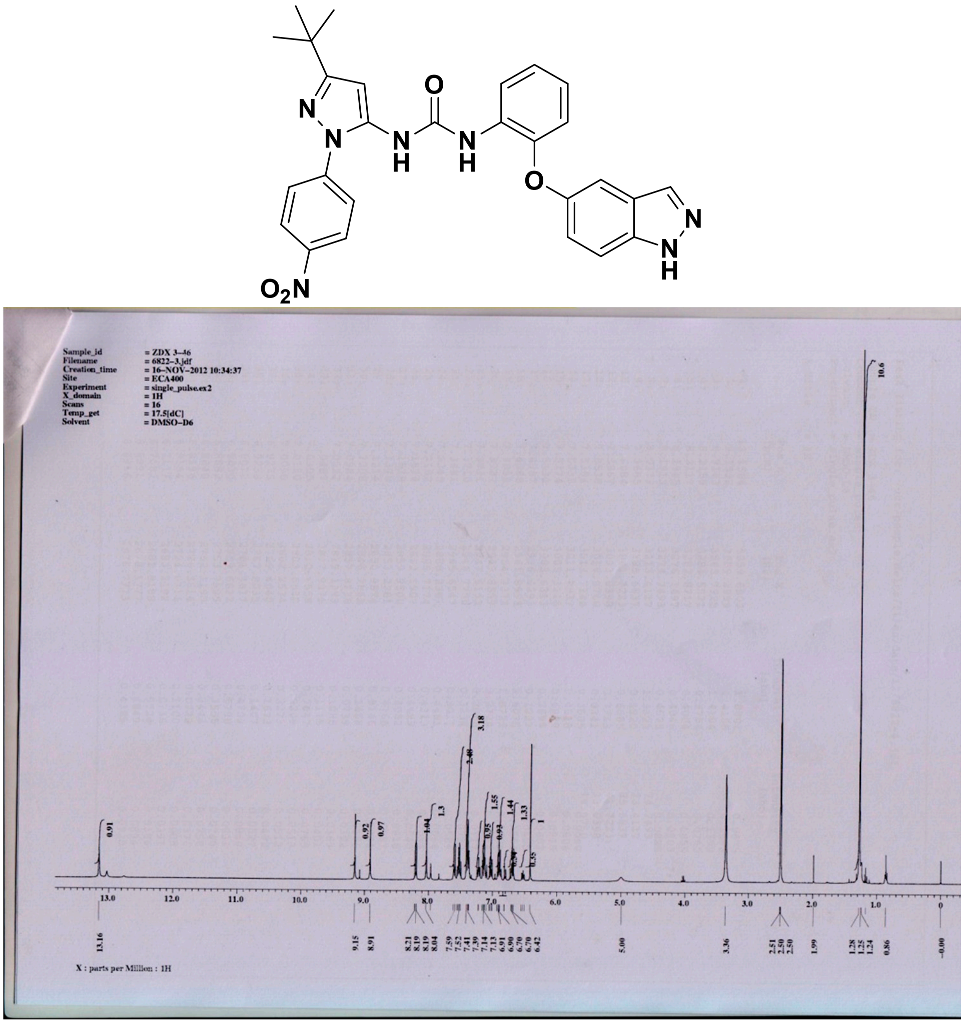

**Figure S39.** 1-(2-((1H-indazol-5-yl)oxy)phenyl)-3-(3-(tert-butyl)-1-(4-nitrophenyl)-1H-pyrazol-5-yl)urea (29h).

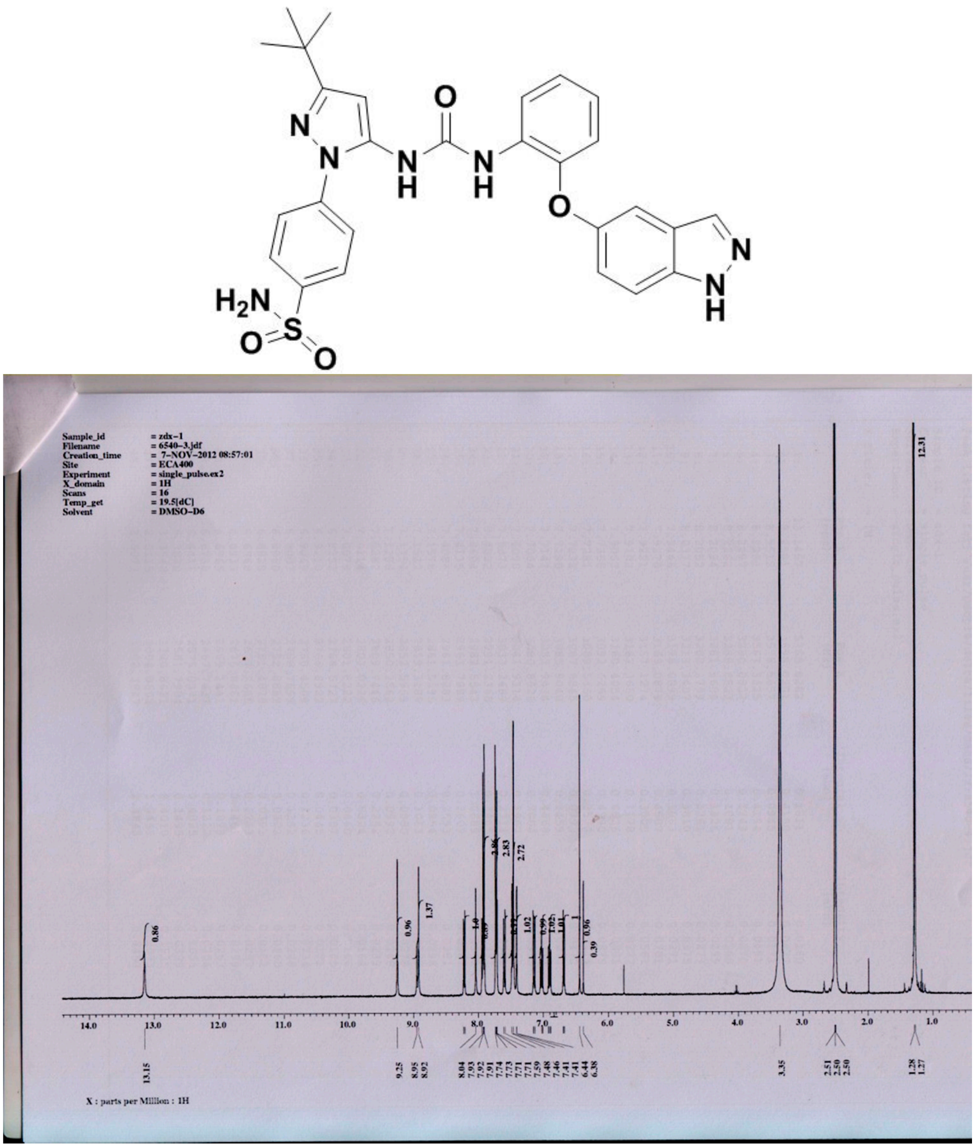


**Figure S40.** 4-(5-(3-(2-((1H-indazol-5-yl)oxy)phenyl)ureido)-3-(tert-butyl)-1H-pyrazol-1-yl)benzenesulfonamide (**29i**).

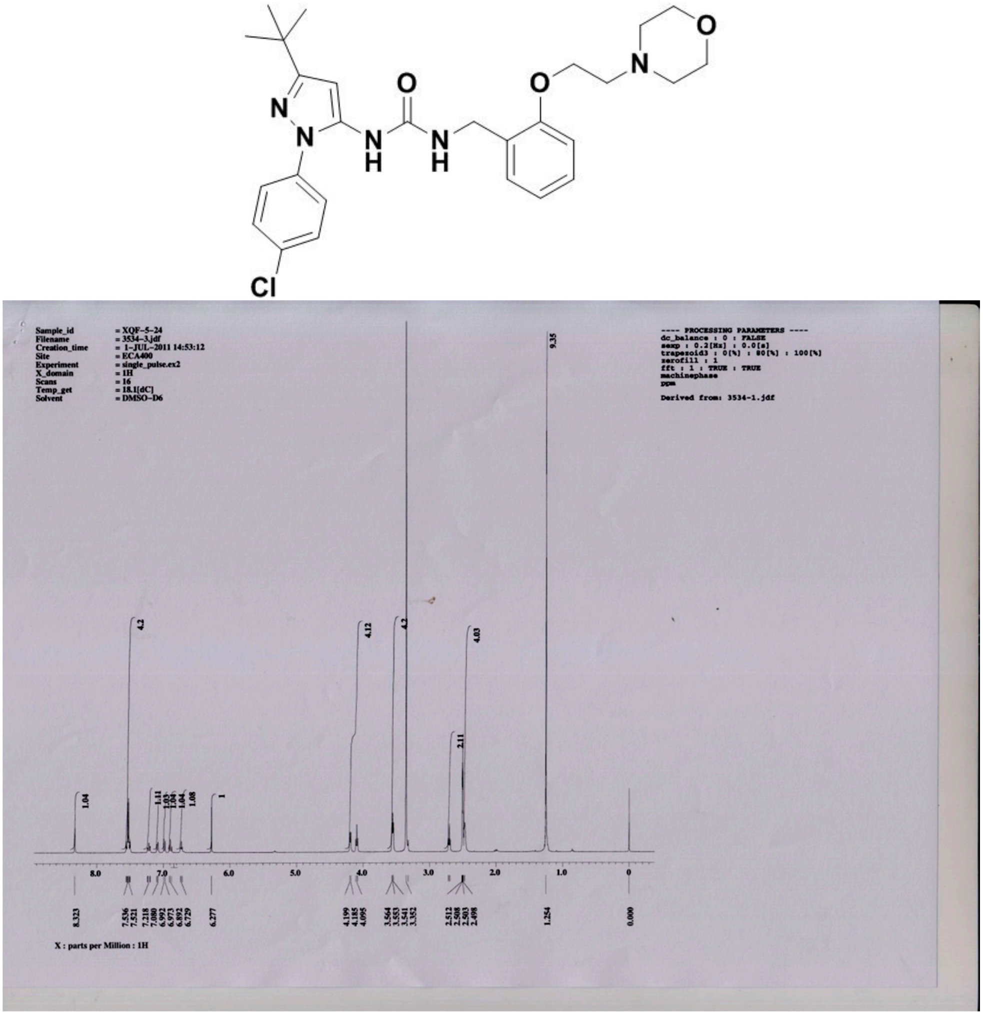


**Figure S41.** 1-(3-(tert-butyl)-1-(4-chlorophenyl)-1H-pyrazol-5-yl)-3-(2-(2-morpholinoethoxy)benzyl)urea (**30a**).

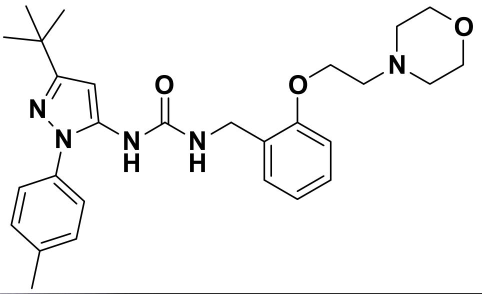

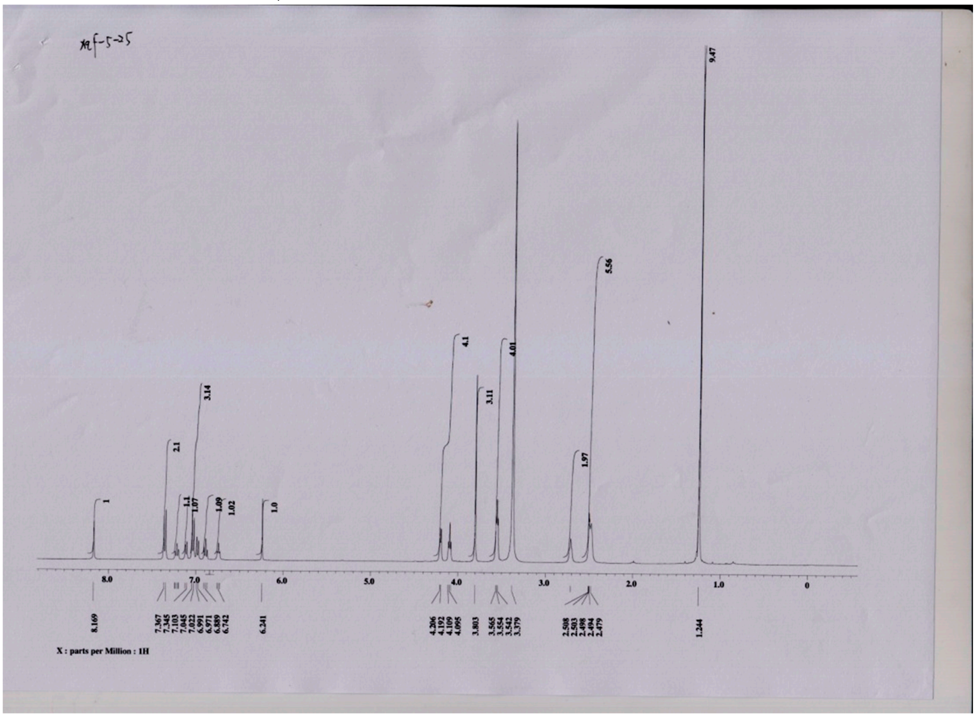

**Figure S42.** 1-(3-(tert-butyl)-1-(p-tolyl)-1H-pyrazol-5-yl)-3-(2-(2-morpholinoethoxy)benzyl)urea (**30b**).

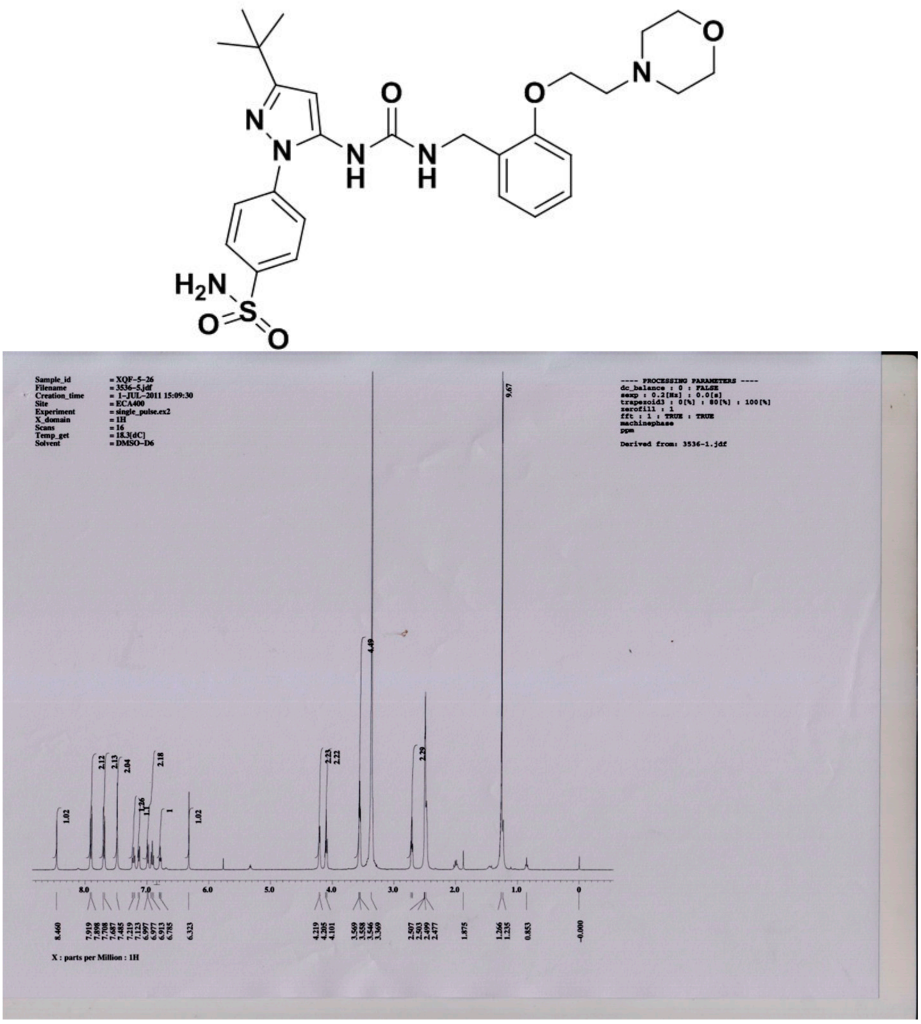


**Figure S43.** 4-(3-(tert-butyl)-5-(3-(2-(2-morpholinoethoxy)benzyl)ureido)-1H-pyrazol-1-yl)benzenesulfonamide (**30c**).

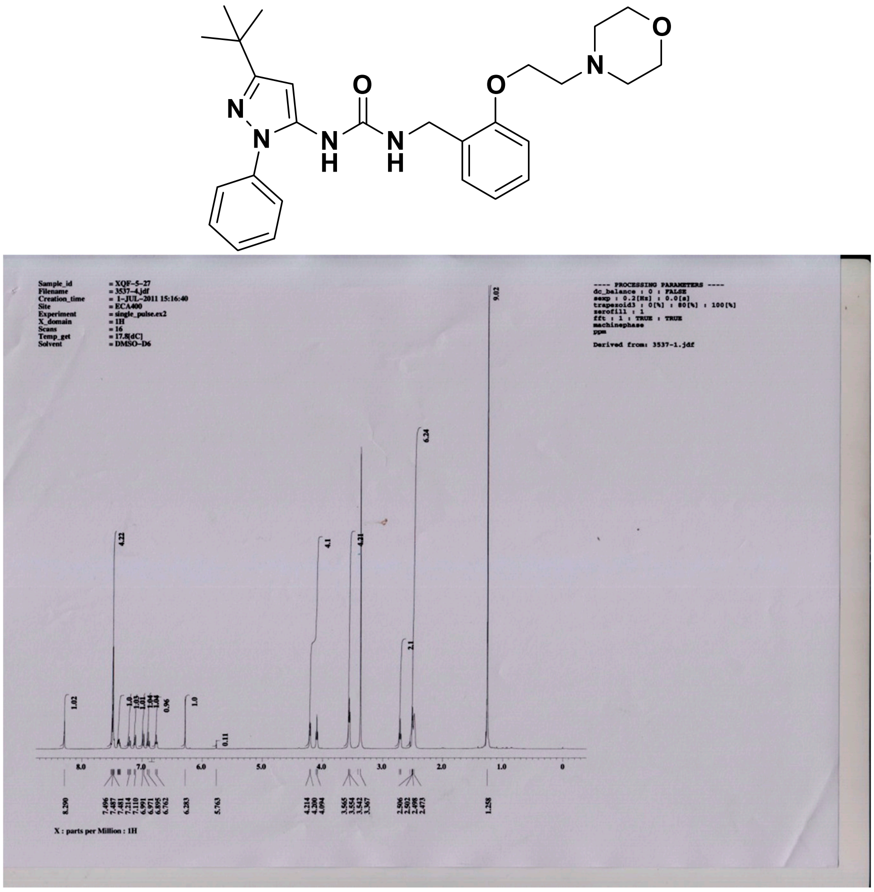

**Figure S44.** 1-(3-(tert-butyl)-1-phenyl-1*H*-pyrazol-5-yl)-3-(2-(2-morpholinoethoxy)benzyl)urea (**30d**).
